# Design and synthesis of benzopyran-based inhibitors of the hypoxia-inducible factor-1 pathway with improved water solubility

**DOI:** 10.1080/14756366.2017.1347784

**Published:** 2017-08-02

**Authors:** Jalisa H. Ferguson, Zeus De Los Santos, Saroja N. Devi, Stefan Kaluz, Erwin G. Van Meir, Sarah K. Zingales, Binghe Wang

**Affiliations:** a Department of Chemistry, Georgia State University, Atlanta, GA, USA;; b Departments of Neurosurgery and Hematology and Medical Oncology, School of Medicine and Winship Cancer Institute, Emory University, Atlanta, GA, USA;; c Department of Chemistry and Physics, Armstrong State University, Savannah, GA, USA

**Keywords:** Hypoxia, solubility, HIF-1

## Abstract

While progress has been made in treating cancer, cytotoxic chemotherapeutic agents are still the most widely used drugs and are associated with severe side-effects. Drugs that target unique molecular signalling pathways are needed for treating cancer with low or no intrinsic toxicity to normal cells. Our goal is to target hypoxic tumours and specifically the hypoxia inducible factor (HIF) pathway for the development of new cancer therapies. To this end, we have previously developed benzopyran-based HIF-1 inhibitors such as arylsulfonamide KCN1. However, KCN1 and its earlier analogs have poor water solubility, which hamper their applications. Herein, we describe a series of KCN1 analogs that incorporate a morpholine moiety at various positions. We found that replacing the benzopyran group of KCN1 with a phenyl group with a morpholinomethyl moiety at the para positions had minimal effect on potency and improved the water solubility of two new compounds by more than 10-fold compared to KCN1, the lead compound.

## Introduction

Cancer is one of the leading causes of death, second only to heart disease^1^. One of the hallmarks of cancer is the formation of hypoxic areas inside of solid tumours^2^. This hypoxic tumour microenvironment leads to many changes such as the upregulation of pro-angiogenic and pro-glycolytic pathways, as well as increases in cell proliferation, genetic instability, and metastatic potential^3^. A major mediator of the hypoxic response is the hypoxia inducible factor (HIF) pathway^4^. HIF is a heterodimeric transcription factor consisting of two subunits, HIF-α, the stability of which is regulated by oxygen, and HIF-1β, which is constitutively expressed^5^. There are three known isoforms of HIF-α, HIF-1α, HIF-2α, and HIF-3α, with HIF-1α being the most commonly expressed and most extensively studied. Under normoxic conditions, HIF-α subunits are hydroxylated by a prolyl hydroxylase (PHD2) using molecular oxygen and then degraded *via* a VHL-dependent ubiquitination pathway^6^. Under hypoxic conditions, however, HIF-α subunits are stabilised, heterodimerise with HIF-1β and recruit co-activators such as p300 and CBP, to form active transcription complexes that bind to 5′-HREs (hypoxia response elements) in promoter regions of hypoxia-inducible genes^7^. Increased levels of HIF-1α are linked to cancer progression and poor patient outcome. Therefore, HIF is an attractive target for developing anti-cancer therapeutics^8^.

**Figure 1.**
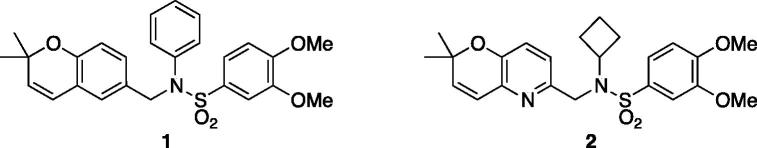
Lead compounds **1** (**KCN1**) and **2** (**64b**).

A library of 10,000 products containing the 2,2-dimethyl-2*H*-chromene moiety^9^ was screened for compounds with HIF inhibitory activity. This led to the identification of a compound designated KCN1 (Figure 1, **1**, *N*-((2,2-dimethyl-2*H*-chromen-6-yl)methyl)-3,4-dimethoxy-*N*-phenylbenzenesulfonamide) showing potent inhibition activity (IC_50_ of ∼0.6 µM) in a HIF-dependent bioassay^10^.

Further *in vivo* studies demonstrated **1**’s very pronounced inhibitory activity against brain, and pancreatic cancers^11^. In addition, **1** was well tolerated in mice; daily treatments with 60 mg/kg for up to 12 weeks had minimal side effects^11^. Neither did **1** nor its analogs demonstrate cytotoxicity, indicating the selective inhibitory effects being based on pathways unique to cancer^11^. Such results strongly suggest that this is a very promising class of compounds and warrant further studies. In fact, a previously synthesised and analysed class of analogs has been developed, which led to the discovery of 64b (Figure 1, **2**, *N*-cyclobutyl-*N*-((2,2-dimethyl-2*H*-pyrano[3,2-*b*]pyridin-6-yl)methyl)-3,4-dimethoxybenzenesulfonamide) with an IC_50_ value of ∼0.3 µM.^12^ However, **1** and its analogs possess poor solubility in water (0.009 µg/mL)^11^. Therefore, dissolution in DMSO is necessary for *in vitro* assays and cremophor:ethanol-based formulations are needed for *in vivo* models. Such a formulation introduces undesirable properties^12^. It is well known that the successful development of potential therapeutics relies on more parameters than potency alone. Other properties, including solubility, can play a critical role. Therefore, we are interested in designing water-soluble analogs of **1** and **2** to address this critical aspect of drug development.

**Figure 2.**
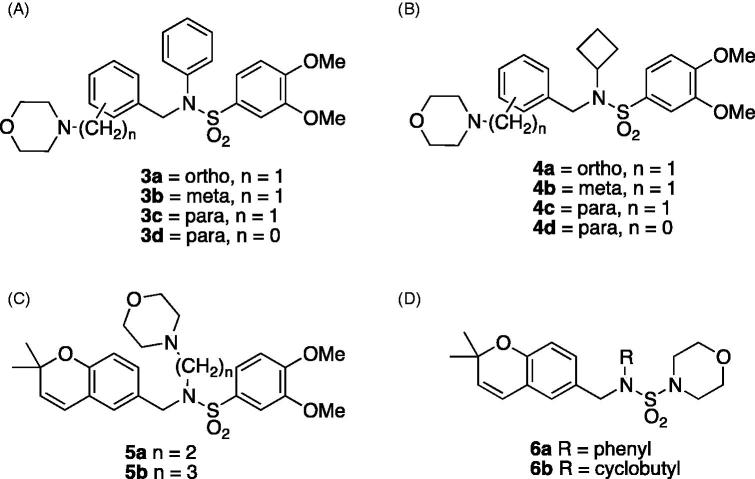
Classes of analogs. (A) Class A, morpholinomethylphenyl in ortho, meta, or para positions, or morpholinophenyl in para position; (B) Class B, morpholinomethylphenyl in ortho, meta, or para positions, or morpholinophenyl in para position; (C) Class C, *n* = 2 or 3; (D) Class D.

## Materials and methods

### Synthesis

#### General methods and materials

All commercial chemicals were of reagent grade from VWR (Radnor, PA), Aldrich (St. Louis, MO), or Oakwood Chemicals (Estill, SC), and were used without further purification unless otherwise indicated. ^1^H and 13C spectra were obtained on a Bruker 400 NMR spectrometer at 400 and 100 MHz, respectively, in deuterated solvent with TMS (*δ* = 0.00 ppm) or deuterated solvent as internal reference. For all reactions, analytical grade solvent was used. Anhydrous solvents were used for all moisture-sensitive reactions. The Mass Spectrometry Facilities at Georgia State University obtained high-resolution mass spectra on a Waters Micromass Q-TOF (ESI) instrument.

#### Typical procedure for morpholine substitution (8a–c)

Benzyl bromide (1 equivalent) was dissolved in acetonitrile. Morpholine (1.1 equivalents) and K_2_CO_3_ (2 equivalents) were added and the reaction was stirred overnight at room temperature. The reaction was filtered through Celite and concentrated to give the product in quantitative yield.

##### 4-(4-Bromobenzyl)morpholine (8a)

^1^H NMR (CDCl_3_): *δ* 7.41 (d, *J* = 8 Hz, 2H), 7.19 (d, *J* = 8 Hz, 2H), 3.67 (t, *J* = 4 Hz, 4H), 3.41 (s, 2H), 2.40 (s, 4H) ppm. 13C NMR (CDCl_3_): *δ* 137.0, 131.4, 130.8, 120.9, 66.9, 62.6, 53.6 ppm. HRMS (ESI) *m/z* calculated for C_11_H_15_NOBr [(M + H)^+^] 256.0337, found 256.0333.

##### 4-(3-Bromobenzyl)morpholine (8b)

^1^H NMR (CDCl_3_): *δ* 7.46 (s, 1H), 7.32 (d, *J* = 8 Hz, 1H), 7.20 (d, *J* = 7 Hz, 1H), 7.12 (t, *J* = 8 Hz, 1H), 3.64 (d, *J* = 4 Hz, 4H), 3.39 (s, 2H), 2.37 (s, 4H) ppm. 13C NMR (CDCl_3_): *δ* 140.4, 131.9, 130.2, 129.8, 127.6, 122.5, 66.9, 62.7, 53.6 ppm. HRMS (ESI) *m/z* calculated for C_11_H_15_NOBr [(M + H)^+^] 256.0337, found 256.0348.

##### 4-(2-Bromobenzyl)morpholine (8c)

^1^H NMR (CDCl_3_): *δ* 7.52 (d, *J* = 8 Hz, 1H), 7.46 (d, *J* = 7 Hz, 1H), 7.26 (t, *J* = 7 Hz, 1H), 7.08 (t, *J* = 7 Hz, 1H), 3.71–3.68 (m, 4H), 3.57 (s, 2H), 2.49–2.48 (m, 4H) ppm. 13C NMR (CDCl_3_): *δ* 137.2, 132.8, 130.8, 128.5, 127.2, 124.7, 67.0, 62.2, 53.6 ppm. HRMS (ESI) *m/z* calculated for C_11_H_15_NOBr [(M + H)^+^] 256.0337, found 256.0348.

#### Typical procedure for lithium halogen exchange to form aldehydes (9a–c)

Arylbromide (1 equivalent) was dissolved in anhydrous THF under N_2_ and cooled in a dry ice and acetone bath for 30 min before treatment with *n*-buLi (1.4 equivalents). After 30 additional minutes, anhydrous DMF (1.4 equivalents) was added and stirring continued 1 h. The reaction was quenched with saturated NH_4_Cl, taken up in ethyl acetate, washed with brine, dried over Mg_2_SO_4_, and concentrated *in vacuo*. Purification by column chromatography was performed in 4:1 hexanes/ethyl acetate.

##### 4-(Morpholinomethyl)benzaldehyde (9a)

Yield: 74%. ^1^H NMR (CDCl_3_): *δ* 9.96 (s, 1H), 7.81 (d, *J* = 8 Hz, 2H), 7.49 (d, *J* = 8 Hz, 2H), 3.68–3.68 (m, 4H), 3.54 (s, 2H), 2.43 (m, 4H) ppm. 13C NMR (CDCl_3_): *δ* 191.9, 145.3, 135.6, 129.8, 129.5, 66.9, 63.0, 53.6 ppm. HRMS *m/z* calculated for C_12_H_16_NO_2_ [(M + H)^+^] 206.1181, found 206.1182.

##### 3-(Morpholinomethyl)benzaldehyde (9b)

Yield: 88%. ^1^H NMR (CDCl_3_): *δ* 9.92 (s, 1H), 7.77 (s, 1H), 7.69 (d, *J* = 8 Hz, 1H), 7.54 (d, *J* = 8 Hz, 2H), 7.41 (t, *J* = 8 Hz, 1H), 3.63 (m, 4H), 3.50 (s, 2H), 2.39 (m, 4H) ppm. 13C NMR (CDCl_3_): *δ* 192.2, 138.8, 136.5, 135.2, 130.2, 129.0, 128.7, 66.7, 62.6, 53.4 ppm. HRMS *m/z* calculated for C_12_H_16_NO_2_ [(M + H)^+^] 206.1181, found 206.1183.

##### 2-(Morpholinomethyl)benzaldehyde (9c)

Yield: 85%. ^1^H NMR (CDCl_3_): *δ* 10.37 (s, 1H), 7.81 (d, *J* = 8 Hz, 1H), 7.44 (d, *J* = 8 Hz, 1H), 7.37–7.33 (m, 2H), 3.76 (s, 2H), 3.58–3.57 (m, 4H), 2.40–2.39 (m, 4H) ppm. 13C NMR (CDCl_3_): δ 192.0, 140.4, 135.0, 133.2, 130.6, 129.4, 127.9, 67.0, 66.9, 60.0, 53.5, 53.3 ppm. HRMS *m/z* calculated for C_12_H_16_NO_2_ [(M + H)^+^] 206.1181, found 206.1186.

#### Procedure for *2,2-dimethyl-2H-chromene-6-carbaldehyde (12)*

Synthesised and purified as described in previous examples^13^. Yield: 37% over two steps. ^1^H NMR (CDCl_3_): *δ* 9.83 (s, 1H), 7.64 (d, *J* = 8 Hz, 1H), 7.52 (s, 1H), 6.87 (d, *J* = 8 Hz, 2H), 6.37 (d, *J* = 10 Hz, 1H), 5.70 (d, *J* = 10 Hz, 1H), 1.47 (s, 6H) ppm.

#### Typical procedure for reductive amination with aniline (10a–d, 14a)

Aldehyde (1 equivalent), NaBH_4_ (1.5 equivalents), and InCl_3_ (0.15 equivalents) were dissolved in anhydrous ACN under inert gas. Aniline (1.5 equivalents) was added and the reaction was stirred until completion as monitored by TLC (typically ∼20 min). The reaction was quenched with saturated NH_4_Cl, taken up in ethyl acetate, washed with brine, dried over MgSO_4_, and concentrated. Column chromatography (1:1 hexane/ethyl acetate) was used to yield the final pure product.

##### *N*-(4-(Morpholinomethyl)benzyl)aniline (10a)

Yield: 60%. ^1^H NMR (CDCl_3_): *δ* 7.23–7.17 (m, 4H), 6.79–6.66 (m, 5H), 4.34 (s, 2H), 3.74 (m, 4H), 3.52 (s, 2H), 2.74 (m, 4H) ppm. 13C NMR (CDCl_3_): *δ* 148.2, 138.4, 136.8, 129.5, 129.3, 127.5, 118.6, 117.6, 115.1, 112.9, 67.0, 63.2, 53.6, 48.1 ppm. HRMS *m/z* (ESI) calculated for C_18_H_23_N_2_O [(M + H)^+^] 283.1810, found 283.1805.

##### *N*-(3-(Morpholinomethyl)benzyl)aniline (10b)

Yield: 60%. ^1^H NMR (CDCl_3_): *δ* 7.36–7.17 (m, 6H), 6.76–6.65 (m, 3H), 4.35 (s, 2H), 3.73–3.72 (m, 4H), 3.52 (s, 2H), 2.45 (m, 4H) ppm. 13C NMR (CDCl_3_): 148.1, 139.5, 138.1, 129.3, 128.6, 128.3, 128.1, 126.4, 117.6, 112.9, 67.0, 63.4, 53.6, 48.3 ppm. HRMS (ESI) *m/z* calculated for C_18_H_23_N_2_O [(M + H)^+^] 283.1810, found 283.1809.

##### *N*-(2-(Morpholinomethyl)benzyl)aniline (10c)

Yield: 54%. ^1^H NMR (CDCl_3_): *δ* 7.44 (d, *J* = 7 Hz, 1H), 7.32–7.22 (m, 5H), 6.75 (d, *J* = 7 Hz, 3H), 5.37 (bs, 1H), 4.39 (s, 2H), 3.75 (m, 4H), 3.57 (s, 2H), 2.51 (m, 4H) ppm. 13C NMR (CDCl_3_): 148.6, 138.9, 135.8, 131.5, 130.0, 129.3, 128.2, 127.2, 117.4, 113.1, 67.1, 61.7, 53.5, 46.9 ppm. HRMS (ESI) *m/z* calculated for C_18_H_23_N_2_O [(M + H)^+^] 283.1810, found 283.1805.

##### *N*-(4-Morpholinobenzyl)aniline (10d)

Yield: 25%. ^1^H NMR (CDCl_3_): *δ* 7.33 (d, *J* = 8 Hz, 2H), 7.22 (t, *J* = 8 Hz, 2H), 6.94 (d, *J* = 8 Hz, 2H), 6.76 (t, *J* = 7 Hz, 1H), 6.68 (d, *J* = 8 Hz, 2H), 4.28 (s, 2H), 4.00 (bs, 1H), 3.91–3.90 (m, 4H), 3.19–3.18 (m, 4H) ppm. 13C NMR (CDCl_3_): *δ* 150.6, 148.3, 130.8, 129.3, 128.7, 117.5, 115.9, 112.9, 67.0, 49.5, 47.8 ppm. HRMS (ESI) *m/z* calculated for C_17_H_21_N_2_O [(M + H)^+^] 269.1654, found 269.1659.

##### *N*-((2,2-Dimethyl-2*H*-chromen-6-yl)methyl)aniline (14a)

Yield: 80%. ^1^H NMR (CDCl_3_): *δ* 7.26–7.21 (m, 3H), 6.97 (d, *J* = 7 Hz, 1H), 6.87 (d, *J* = 8 Hz, 1H), 6.79–6.72 (m, 3H), 6.39 (d, *J* = 10 Hz, 1H), 5.68 (d, *J* = 10 Hz, 1H), 4.37 (s, 2H), 4.06 (bs, 1H) 1.52 (s, 6H) ppm. 13C NMR (CDCl_3_): *δ* 150.8, 148.5, 130.6, 129.2, 128.8, 126.5, 125.5, 122.5, 121.1, 120.5, 117.4, 113.2, 76.4, 43.1, 28.1 ppm. HRMS (ESI) *m/z* calculated for C_18_H_20_NO [(M + H)^+^] 266.1545, found 266.1548.

#### Typical procedure for reductive amination with cyclobutyl and alkylmorpholino amines (11a–d, 13a–b, 14b)

Aldehyde (1 equivalent) and amine (1 equivalent) were dissolved in anhydrous MeOH under inert gas and the reaction was stirred overnight at room temperature. NaBH_4_ (1.6 equiv.) was added and the reaction stirred for an additional hour. The reaction was quenched with NaOH (1 M), stirred for an hour, then taken up in ethyl acetate, washed with brine, dried over MgSO_4_, concentrated, and taken directly to the next step without further purification.

##### *N*-(4-(Morpholinomethyl)benzyl)cyclobutanamine (11a)

Crude yield: 89%. ^1^H NMR (CDCl_3_): *δ* 7.32–7.26 (m, 4H), 3.69–3.68 (m, 4H), 3.47 (s, 2H), 3.29 (quintet, *J* = 7 Hz, 1H), 2.42 (m, 4H), 2.22–2.21 (m, 2H), 1.63–1.62 (m, 4H) ppm. 13C NMR (CDCl_3_): *δ* 139.3, 136.3, 129.4, 128.1, 67.0, 63.2, 53.6, 50.8, 31.2, 31.1, 15.0, 14.8 ppm. HRMS (ESI) *m/z* calculated for C_16_H_25_N_2_O [(M + H)^+^] 261.1967, found 261.1961.

##### *N*-(3-(Morpholinomethyl)benzyl)cyclobutanamine (11b)

Crude yield: 88% unpurified. ^1^H NMR (CDCl_3_): 7.32–7.19 (m, 4H), 3.69 (m, 6H), 3.49–3.48 (m, 2H), 3.30 (quintet, *J* = 7 Hz, 1H), 2.43 (m, 4H), 2.24–2.20 (m, 2H), 1.74–1.63 (m, 4H) ppm. 13C NMR (CDCl_3_): 140.3, 137.9, 129.0, 128.6, 127.8, 127.1, 66.9, 63.1, 53.7, 53.6, 51.0, 31.1, 14.8 ppm. HRMS (ESI) *m/z* calculated for C_16_H_25_N_2_O [(M + H)^+^] 261.1967, found 261.1963.

##### *N*-(2-(Morpholinomethyl)benzyl)cyclobutanamine (11c)

Crude yield: 94%. ^1^H NMR (CDCl_3_): *δ* 7.30–7.16 (m, 4H), 3.67 (s, 2H), 3.64 (s, 4H), 3.49 (s, 2H), 3.28–3.27 (m, 1H), 2.53 (s, 1H), 2.43 (s, 4H), 2.19–2.17 (m, 2H), 1.70–1.63 (m, 4H) ppm. 13C NMR (CDCl_3_): *δ* 140.3, 135.7, 131.3, 130.6, 127.9, 126.7, 67.0, 61.7, 53.9, 53.4, 49.7, 31.0, 15.1 ppm. HRMS (ESI) *m/z* calculated for C_16_H_25_N_2_O [(M + H)^+^] 261.1967, found 261.1962.

##### *N*-(4-Morpholinobenzyl)cyclobutanamine (11d)

Crude yield: 90%. ^1^H NMR (CDCl_3_): *δ* 7.22 (d, *J* = 8 Hz, 2H), 6.87 (d, *J* = 8 Hz, 2H), 3.86–3.84 (m, 4H), 3.62 (s, 2H), 3.28 (quintet, *J* = 6.8 Hz, 1H), 3.14–3.11 (m, 4H), 2.22–2.19 (m, 2H), 1.70–1.62 (m, 4H) ppm. 13C NMR (CDCl_3_): *δ* 150.3, 131.9, 129.1, 115.7, 66.9, 53.5, 50.4, 49.5, 31.1, 14.8 ppm. HRMS (ESI) *m/z* calculated for C_15_H_23_N_2_O [(M + H)^+^] 247.1810, found 247.1819.

##### *N*-((2,2-dimethyl-2*H*-chromen-6-yl)methyl)-2-morpholinoethanamine (13a)

Crude yield: 90%. ^1^H NMR (CDCl_3_): *δ* 7.16 (dd, *J* = 8, 22 Hz, 1H), 7.00 (d, *J* = 8 Hz, 1H), 6.69 (d, *J* = 8 Hz, 1H), 6.27 (d, *J* = 10 Hz, 1H), 5.57 (d, *J* = 10 Hz, 1H), 3.72–3.64 (m, 6H), 3.09 (s, 1H), 2.66–2.64 (m, 2H), 2.47–2.44 (m, 2H), 2.35 (m, 4H), 1.39 (s, 6H) ppm. 13C NMR (CDCl_3_): *δ* 152.0, 130.9, 128.9, 128.7, 126.2, 122.2, 121.5, 116.1, 76.1, 66.9, 57.9, 53.6, 53.2, 44.9, 27.9 ppm. HRMS (ESI) *m/z* calculated for C_18_H_27_N_2_O_2_ [(M + H)^+^] 303.2073, found 303.2063.

##### *N*-((2,2-Dimethyl-2*H*-chromen-6-yl)methyl)-3-morpholinopropan-1-amine (13b)

Crude yield: 89%. ^1^H NMR (CDCl_3_): *δ* 7.18–7.10 (m, 1H), 6.98 (d, *J* = 8 Hz, 1H), 6.67 (d, *J* = 8 Hz, 1H), 6.24 (d, *J* = 10 Hz, 1H), 5.55 (d, *J* = 10 Hz, 1H), 3.68–3.62 (m, 6H), 2.63 (m, 2H), 2.37–2.35 (m, 4H), 1.67–1.58 (m, 2H), 1.58 (m, 2H), 1.37 (s, 6H) ppm. 13C NMR (CDCl_3_): δ 151.9, 132.3, 130.9, 128.8, 126.1, 122.3, 121.5, 116.1, 73.9, 66.9, 57.3, 53.7, 47.9, 29.6, 27.9, 26.4 ppm. HRMS (ESI) *m/z* calculated for C_19_H_29_N_2_O_2_ [(M + H)^+^] 317.2229, found 317.2237.

##### *N*-((2,2-Dimethyl-2*H*-chromen-6-yl)methyl)aniline (14a)

Crude yield: 90%. ^1^H NMR (CDCl_3_): *δ* 7.26–7.21 (m, 3H), 6.96 (d, *J* = 7 Hz, 1H), 6.87 (d, *J* = 8 Hz, 1H), 6.79–6.72 (m, 3H), 6.39 (d, *J* = 10 Hz, 1H), 5.68 (d, *J* = 10 Hz, 1H), 4.73 (s, 2H), 4.06 (bs, 1H), 1.52 (s, 6H) ppm. 13C NMR (CDCl_3_): *δ* 150.8, 148.5, 130.6, 129.2, 128.8, 126.5, 125.5, 122.5, 121.1, 120.5, 117.4, 113.2, 76.4, 43.1, 28.2 ppm. HRMS (ESI) *m/z* calculated for C_18_H_20_NO [(M + H)^+^] 266.1545, found 266.1548.

##### *N*-((2,2-Dimethyl-2*H*-chromen-6-yl)methyl)cyclobutanamine (14b)

Crude yield: 98%. ^1^H NMR (CDCl_3_): *δ* 7.03 (d, *J* = 8 Hz, 1H), 6.95 (s, 1H), 6.72 (d, *J* = 8 Hz, 1H), 6.31 (d, *J* = 10 Hz, 1H), 5.60 (d, *J* = 10 Hz, 1H), 3.56 (s, 2H), 3.32–3.26 (m, 1H), 2.22 (m, 2H), 1.72–1.69 (m, 4H), 1.42 (s, 6H) ppm. 13C NMR (CDCl_3_): *δ* 151.9, 132.5, 130.8, 128.9, 126.2, 122.3, 121.2, 116.1, 76.1, 53.5, 50.5, 31.1, 27.9, 14.8 ppm. HRMS (ESI) *m/z* calculated for C_16_H_22_NO [(M + H)^+^] 244.1701, found 244.1697.

#### Typical procedure for sulfonylation with 3,4-dimethoxybenzenesulfonyl chloride (3a–d, 4a–d, 5a–b)

Amine (1 equivalent) was dissolved in DCM. K_2_CO_3_ (2 equivalents) and 3,4-dimethoxybenzenesulfonyl chloride (2 equivalents) were added. The reaction was stirred overnight at room temperature, then washed with brine, dried over MgSO_4_, and concentrated. The product was purified by column chromatography in 4:1 or 1:1 hexane/ethyl acetate.

##### 3,4-Dimethoxy-*N*-(4-(morpholinomethyl)benzyl)-*N*-phenylbenzenesulfonamide (3a)

Yield: 11%. ^1^H NMR (CDCl_3_): *δ* 7.36 (d, *J* = 7 Hz, 1H), 7.35 (s, 1H), 7.22–7.20 (m, 6H), 7.04–7.02 (m, 2H), 6.97–6.94 (m, 2H), 4.72 (s, 1H), 3.98 (s, 3H), 3.77 (s, 3H), 3.70–3.69 (m, 4H), 3.44 (s, 2H), 2.40 (m, 4H) ppm. 13C NMR (CDCl_3_): *δ* 152.6, 148.7, 139.2, 135.0, 130.2, 129.2, 129.0, 128.8, 128.4, 127.8, 127.5, 121.4, 110.4, 110.4, 66.9, 63.0, 56.2, 56.01, 54.4, 53.5 ppm. HRMS (ESI) *m/z* calculated for C_26_H_31_N_2_O_5_S [(M + H)^+^] 483.1954, found 483.1956.

##### 3,4-Dimethoxy-*N*-(3-(morpholinomethyl)benzyl)-*N*-phenylbenzenesulfonamide (3b)

Yield: 64%. ^1^H NMR (CDCl_3_): *δ* 7.36–7.31 (m, 2H), 7.17–7.09 (m, 7H), 6.98–6.91 (m, 4H), 4.69 (s, 2H), 3.95 (s, 3H), 3.73 (s, 3H), 3.63 (t, *J* = 4 Hz, 4H), 3.39–3.37 (m, 2H), 2.27 (m, 4H) ppm. 13C NMR (CDCl_3_): *δ* 152.7, 148.8, 139.1, 137.8, 135.9, 130.1, 129.6, 129.1, 128.8, 128.6, 128.4, 127.8, 127.6, 121.5, 110.5, 67.0, 63.2, 56.3, 56.2, 54.5, 53.5, 48.5 ppm. HRMS (ESI) *m/z* calculated for C_26_H_31_N_2_O_5_S [(M + H)^+^] 483.1948, found 483.1928.

##### 3,4-Dimethoxy-*N*-(2-(morpholinomethyl)benzyl)-*N*-phenylbenzenesulfonamide (3c)

Yield: 47%. ^1^H NMR (CDCl_3_): *δ* 7.36 (d, *J* = 8 Hz, 1H), 7.21–7.18 (m, 4H), 7.10–7.09 (m, 3H), 7.03–7.01 (m, 2H), 6.95–6.93 (m, 2H), 4.97 (s, 2H), 3.97 (s, 3H), 3.75 (s, 3H), 3.62 (m, 4H), 3.48 (s, 2H), 2.35 (m, 4H) ppm. 13C NMR (CDCl_3_): *δ* 152.7, 148.8, 139.5, 136.1, 135.2, 130.8, 130.2, 129.7, 128.9, 128.8, 127.8, 127.4, 127.3, 121.7, 110.7, 110.5, 67.2, 61.1, 56.3, 56.2, 53.6, 51.3, 31.0, 30.8, 13.6 ppm. HRMS (ESI) *m/z* calculated for C_26_H_31_N_2_O_5_S [(M + H)^+^] 483.1948, found 483.1941.

##### 3,4-Dimethoxy-*N*-(4-morpholinobenzyl)-*N*-phenylbenzenesulfonamide (3d)

Yield: 40%. ^1^H NMR (CDCl_3_): *δ* 7.35 (d, *J* = 8 Hz, 1H), 7.21 (m, 3H), 7.12 (d, *J* = 7 Hz, 2H), 7.01–6.93 (m, 4H), 6.76 (d, *J* = 7 Hz, 2H), 4.65 (s, 2H), 3.97 (s, 3H), 3.84–3.83 (m, 4H), 3.77 (s, 3H), 3.11–3.10 (m, 4H) ppm. ^13^C NMR (CDCl_3_): *δ* 152.5, 150.5, 148.7, 139.2, 130.3, 129.6, 129.1, 128.7, 127.7, 127.1, 121.4, 115.3, 110.4, 66.8, 56.2, 56.1, 54.1, 49.1 ppm. HRMS (ESI) *m/z* calculated for C_25_H_29_N_2_O_5_S [(M + H)^+^] 469.1797, found 469.1796.

##### *N*-Cyclobutyl-3,4-dimethoxy-*N*-(4-(morpholinomethyl)benzyl)benzenesulfonamide (4a)

Yield: 46%. ^1^H NMR (CDCl_3_): *δ* 7.43 (dd, *J* = 8, 2 Hz, 1H), 7.34–7.25 (m, 5H), 6.94 (d, *J* = 9 Hz, 1H), 4.39 (s, 2H), 4.27 (quintet, *J* = 9 Hz, 1H), 3.96 (s, 3H), 3.92 (s, 3H), 3.75–3.72 (m, 4H), 3.51 (s, 2H), 2.46 (s, 4H), 1.99–1.94 (m, 4H), 1.57–1.52 (m, 2H) ppm. ^13^C NMR (CDCl_3_): *δ* 152.4, 149.0, 137.8, 132.0, 129.4, 127.0, 120.9, 110.6, 109.8, 66.9, 63.0, 56.2, 56.2, 53.5, 52.9, 48.2, 29.2, 15.0 ppm. HRMS (ESI) *m/z* calculated for C_24_H_33_N_2_O_5_S [(M + H)^+^] 461.2110, found 461.2102.

##### *N*-Cyclobutyl-3,4-dimethoxy-*N*-(3-(morpholinomethyl)benzyl)benzenesulfonamide (4 b)

Yield: 81%. ^1^H NMR (CDCl_3_): *δ* 7.43 (dd, *J* = 8, 2 Hz, 1H), 7.30–7.21 (m, 5H), 6.95–6.93 (d, *J* = 8 Hz, 1H), 4.39 (s, 2H), 4.28 (quintet, *J* = 8 Hz, 1H), 3.95 (s, 3H), 3.91 (s, 3H), 3.71 (t, *J* = 4 Hz, 4H), 3.49 (s, 2H), 2.43 (s, 4H), 1.99–1.92 (m, 4H), 1.55–1.48 (m, 2H) ppm. ^13^C NMR (CDCl_3_): *δ* 152.4, 149.0, 138.7, 137.9, 131.9, 128.4, 128.1, 127.8, 126.0, 120.9, 110.5, 109.7, 67.0, 63.3, 56.2, 56.2, 53.6, 52.9, 48.3, 29.2, 15.0 ppm. HRMS (ESI) *m/z* calculated for C_24_H_33_N_2_O_5_S [(M + H)^+^] 461.2110, found 461.2112.

##### *N*-Cyclobutyl-3,4-dimethoxy-*N*-(2-(morpholinomethyl)benzyl)benzenesulfonamide (4c)

Yield: 63%. ^1^H NMR (CDCl_3_): *δ* 7.56 (d, *J* = 8 Hz, 1H), 7.47 (dd, *J* = 8, 2 Hz, 1H), 7.31–7.27 (m, 2H), 7.17 (d, *J* = 7 Hz, 1H), 6.95 (d, *J* = 8 Hz, 1H), 4.68 (s, 2H), 4.45 (quintet, *J* = 8 Hz, 1H), 3.96 (s, 3H), 3.92 (s, 3H), 3.65 (m, 4H), 3.50 (bs, 2H), 2.42 (bs, 4H), 1.93–1.90 (m, 4H), 1.56–1.50 (m, 2H) ppm. ^13^C NMR (CDCl_3_): *δ* 152.4, 149.0, 138.4, 133.4, 132.1, 130.6, 128.0, 127.4, 126.4, 121.0, 110.5, 109.7, 67.1, 61.6, 56.3, 56.2, 53.5, 52.7, 44.5, 29.0, 15.1 ppm. HRMS (ESI) *m/z* calculated for C_24_H_33_N_2_O_5_S [(M + H)^+^] 461.2110, found 461.2095.

##### *N*-Cyclobutyl-3,4-dimethoxy-*N*-(4-morpholinobenzyl)benzenesulfonamide (4d)

Yield: 70%. ^1^H NMR (CDCl_3_): *δ* 7.41 (dd, *J* = 8, 2 Hz, 1H), 7.25–7.21 (m, 3H), 6.92 (d, *J* = 9 Hz, 1H), 6.86 (d, *J* = 9, 2H), 4.32 (s, 2H), 4.20 (quintet, *J* = 8 Hz, 1H), 3.93 (s, 3H), 3.88 (s, 3H), 3.86–3.46 (m, 4H), 3.15–3.12 (m, 4H), 2.02–1.90 (m, 4H), 1.54–1.45 (m, 2H) ppm. ^13^C NMR (CDCl_3_): *δ* 152.4, 150.4, 149.0 132.2, 129.8, 128.2, 120.9, 115.6, 110.6, 109.7, 66.9, 56.2, 56.1, 52.9, 49.4, 48.0, 29.7, 29.3, 15.1 ppm. HRMS (ESI) *m/z* calculated for C_23_H_31_O_5_N_2_S [(M + H)^+^]: 446.1948, found 447.1949.

##### *N*-((2,2-Dimethyl-2*H*-chromen-6-yl)methyl)-3,4-dimethoxy-*N*-(2-morpholinoethyl)benzenesulfonamide (5a)

Yield: 49%. ^1^H NMR (CDCl_3_): *δ* 7.45 (d, *J* = 8 Hz, 1H), 6.93 (t, *J* = 9 Hz, 2H), 6.88–6.85 (m, 1H), 6.67 (d, *J* = 8 Hz, 1H), 6.22 (d, *J* = 10 Hz, 1H), 5.59 (d, *J* = 7 Hz, 1H), 4.23 (s, 2H), 3.93 (s, 3H), 3.89 (s, 3H), 3.59–3.56 (m, 4H), 3.19 (t, *J* = 7 Hz, 2H), 2.30 (t, *J* = 7 Hz, 2H), 2.25 (s, 4H), 1.23 (s, 6H) ppm. ^13^C NMR (CDCl_3_): *δ* 152.7, 152.5, 149.1, 131.8, 131.3, 129.1, 128.3, 126.5, 122.0, 121.4, 121.0, 116.3, 110.6, 109.8, 66.8, 57.3, 57.3, 56.3, 56.2, 53.6, 52.2, 44.4, 27.9 ppm. HRMS (ESI) *m/z* calculated for C_26_H_35_N_2_O_6_S [(M + H)^+^] 503.2210, found 503.2204.

##### *N*-((2,2-Dimethyl-2*H*-chromen-6-yl)methyl)-3,4-dimethoxy-*N*-(3-morpholinopropyl)benzenesulfonamide (5b)

Yield: 15%. ^1^H NMR (CDCl_3_): *δ* 7.43 (d, *J* = 8 Hz, 1H) 6.97 (d, *J* = 8 Hz, 1H), 6.94 (d, *J* = 8 Hz, 1H), 6.87 (s, 1H), 6.68 (d, *J* = 8 Hz, 1H), 6.23 (d, *J* = 10 Hz, 1H), 4.19 (s, 2H), 3.94 (s, 3H), 3.90 (s, 3H), 3.60 (s, 4H), 3.12 (t, *J* = 8, 2H), 2.22 (s, 4H), 2.18 (t, *J* = 7, 2H), 1.59–1.52 (m, 2H), 1.40 (s, 6H) ppm. ^13^C NMR (CDCl_3_): *δ* 152.7, 152.4, 149.1, 131.6, 131.3, 129.3, 128.4, 126.5, 121.9, 121.3, 121.0, 116.3, 110.6, 109.8, 66.9, 56.3, 56.2, 55.9, 53.4, 52.0, 46.2, 28.0, 25.4 ppm. HRMS (ESI) *m/z* calculated for C_27_H_37_N_2_O_6_S [(M + H)^+^] 517. 2367, found 517.2366.

#### Typical procedure for sulfonylation with 4-morpholinosulfonyl chloride (6a–b)

Amine (1 equivalent) was dissolved in dichloroethane. Pyridine (3 equivalents) and 4-morpholinosulfonyl chloride (1.3 equivalents) were added. The reaction was refluxed for 2 days, then concentrated, taken up in ethyl acetate, washed with saturated NH_4_Cl and brine, then dried over MgSO_4_, and concentrated. The residue was then purified by column chromatography in 4:1 hexane/ethyl acetate.

##### *N*-((2,2-Dimethyl-2*H*-chromen-6-yl)methyl)-*N*-phenylmorpholine-4-sulfonamide (6a)

Yield: 17%. ^1^H NMR (CDCl_3_): *δ* 7.32–7.25 (m, 5H), 6.90 (d, *J* = 8 Hz, 1H), 6.83 (s, 1H), 6.65 (d, *J* =8 Hz, 1H), 6.25 (d, *J* = 10 Hz, 1H), 5.60 (d, *J* = 10 Hz, 1H), 4.70 (s, 2H), 3.63–3.62 (m, 4H), 3.17 (m, 4H), 1.42 (s, 6H) ppm. ^13^C NMR (CDCl_3_): *δ* 131.0, 129.6, 129.2, 129.1, 127.9, 126.9, 122.1, 116.2, 66.3, 56.3, 46.5, 28.0 ppm. HRMS (ESI) *m/z* calculated for C_22_H_27_N_2_O_4_S [(M + H)^+^] 415.1692, found 415.1695.

##### *N*-Cyclobutyl-*N*-((2,2-dimethyl-2*H*-chromen-6-yl)methyl)morpholine-4-sulfonamide (6b)

Yield: 16%. ^1^H NMR (CDCl_3_): *δ* 7.04 (dd, *J* = 8, 2 Hz, 1H), 6.95 (s, 1H), 6.72 (d, *J* = 8 Hz, 1H), 6.30 (d, *J* = 10 Hz, 1H), 5.61 (d, *J* = 10 Hz, 1H), 4.35 (s, 2H), 4.19 (quintet, *J* = 8 Hz, 1H), 3.63 (t, *J* = 5 Hz, 4H), 3.09 (t, *J* = 5 Hz, 4H), 2.13 – 2.06 (m, 4H), 1.60 (m, 2H), 1.41 (s, 6H) ppm. ^13^C NMR (CDCl_3_): *δ* 152.4, 131.2, 130.6, 128.2, 125.5, 122.4, 121.4, 116.4, 76.4, 66.4, 53.0, 48.8, 46.2, 29.6, 28.1, 14.8 ppm. HRMS (ESI) *m/z* calculated for C_20_H_29_N_2_O_4_S [(M + H)^+^] 393.1843, found 393.1834.

### Lipophilicity and solubility prediction

The *in silico* log *D* and log *S* values of all analogs were predicted using Calculator Plugins from MarvinSketch 4.3.0, 2017, ChemAxon (http://www.chemaxon.com), with results detailed in Table 1. Graphical representations of the log *D* and log *S* from pH 0 to 14 are provided in the Supplemental Information.

**Table 1.** Structures, HRE-luciferase reporter inhibitory activity, cLog *D*, and cLog *S* of analogs.

Compound	Structure	IC_50_ (μM)	cLog *D*	cLog *S*
**1**		∼0.6	4.99	−6.37
**2**		∼0.3	3.34	−4.53
**3a**		0.9	3.69	−4.39
**3b**		>5	3.71	−4.41
**3c**		>5	3.80	−4.50
**3d**		3.8	3.98	−5.19
**4a**		1.0	2.94	−3.36
**4b**		>5	2.96	−3.38
**4c**		>5	3.05	−3.47
**4d**		2.6	3.24	−4.15
**5a**		>5	3.13	−4.23
**5b**		>5	3.17	−4.37
**6a**		>5	2.97	−5.17
**6b**		>5	2.22	−4.12

### Luciferase assay

These analogs were first evaluated for their ability to inhibit hypoxia-induced HIF transcriptional activity in LN229-HRE-luciferase glioma cells as described previously^10–12^. Their inhibitory activities are presented as IC_50_ in Table 1.

### Solubility studies using dynamic light scattering

To further investigate the true enhancement of solubility, particle aggregation was examined using dynamic light scattering (DLS). Selected compounds were treated according to the following procedure:

1. All centrifuge tubes and cuvettes were rinsed with either DCM or water and then vacuum dried before use to remove dust and any particulates.
2. Stock solutions (10 mM) of each compound of interest were prepared in filtered DMSO.
3. Six dilutions (0, 10, 20, 30, 50, and 100 µM) were prepared in filtered de-ionised water with 1% DMSO and allowed to rest at room temperature for 24 h after vortex.
4. DLS analysis was performed for each concentration on the Brookhaven Instrument Corporation, NanoBrook 90Plus Particle Size Analyzer, Version 5.20 (Holtsville, NY).
5. Additional experiments were performed at specific concentrations for each compound as follows: 0, 1, 3, 10, and 20 µM concentrations of **1**; 0, 10, 12, and 20 µM of **2**; 0, 5, 7, and 10 µM of **3a**; and 0, 10, 20, 30, and 50 µM concentrations of **4a**.
6. Additional experiments were repeated in filtered PBS* with 1% DMSO as follows: 0, 0.5, 1, 2, 3, and 5 µM concentrations of **1**; 0, 5, 7, 10, 12, and 15 µM of **2**; 0, 10, 12, 15, and 20 µM of **3a**; and 0, 10, 20, 30, and 50 µM concentrations of **4a**.

*Experiments in PBS were carried out the same way as the experiments in water except DMSO stock solutions were made at 5 mM and the PBS diluted samples rested for 1 h before particle analysis.

## Results and discussion

### Design

In considering ways to improve water solubility without compromising potency, we thought about introducing a commonly used morpholino moiety, which is known to help improve water solubility. In doing so, we were interested in searching for the optimal position, which would not negatively affect potency. Therefore, we devised four classes of compounds (Figure 2): Class A incorporates a morpholinomethylphenyl or morpholinophenyl moiety instead of the 2,2-dimethyl-2*H*-chromene moiety and maintains the *N*-phenyl group; Class B incorporates either a morpholinomethylphenyl or morpholinophenyl moiety instead of the 2,2-dimethyl-2*H*-chromene moiety and substitutes the *N*-phenyl group for an *N*-cyclobutyl group; Class C has either a 2,2-dimethyl-2*H*-chromene or *N*-(2,2-dimethyl-2*H*-pyrano[3,2-*b*]pyridin-6-yl) moiety and either an *N*-ethylmorpholino or *N*-propylmorpholino group instead of the *N*-phenyl; and Class D has the 2,2-dimethyl-2*H*-chromene moiety with a *N*-phenyl*-*morpholine-4-sulfonamide.

### Chemistry

**Scheme 1.**
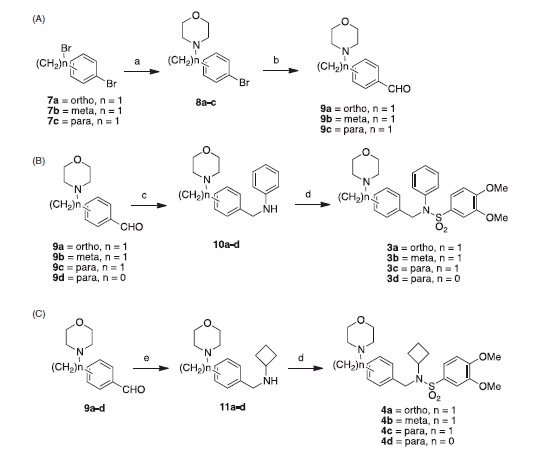
Synthesis of Class A & B compounds. (A) Synthesis of precursors. (B) Synthesis of Class A. (C) Synthesis of Class B. Reagents and conditions: (a) morpholine, K_2_CO_3_, ACN, room temperature, overnight; (b) BuLi, DMF, THF, −78 °C, 1 h; (c) aniline, InCl_3_, NaBH_4_, ACN, 20 min; (d) 3,4-dimethoxybenzenesulfonyl chloride, K_2_CO_3_, DCM, overnight; (e) cyclobutylamine, NaBH_4_, MeOH, overnight.

Synthesis of Class A compounds (Scheme 1) was accomplished in four steps from 2-, 3-, or 4-bromomethylbenzylbromide **7a–c** or in two steps from 4-morpholinobenzaldehyde **9d**. Intermediates **7a–c** were substituted with morpholine to yield morpholinomethylbenzylbromides **8a–c** in quantitative yield. Next, the phenyl bromides **8a–c** were converted to benzaldehydes **9a–c***via* lithium-halogen exchange at −78 °C under inert gas. The aryllithium intermediate was treated with DMF as the electrophile *in situ* to generate the final benzaldehydes **9a–c**. The aldehydes **9a–d** underwent reductive amination with aniline to afford the secondary amines **10a–d**. Finally, **10a–d** were reacted with 3,4-dimethoxybenzenesulfonyl chloride to afford sulfonamides **2a–d**. Class B compounds (Scheme 1(C)) were synthesised in almost the same fashion as Class A, except that reductive amination of **9a–d** was with cyclobutylamine and was not catalysed by any Lewis acid.

**Scheme 2.**
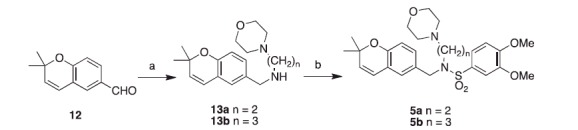
Synthesis of Class C compounds. Reagents and conditions: (a) amine, NaBH_4_, MeOH, overnight; (b) 3,4-dimethoxybenzenesulfonyl chloride, K_2_CO_3_, DCM, overnight.

Class C compounds were synthesised (Scheme 2) from 2,2-dimethyl-2*H*-chromene-6-carbaldehyde **12**, which was readily synthesised from published procedures^13^. The aldehyde **12** underwent reductive amination with either ethylaminomorpholine or propylaminomorpholine to give secondary amines **13a–b**, which were then reacted with 3,4-dimethoxybenzenesulfonyl chloride to afford sulfonamides **5a–b**.

**Scheme 3.**
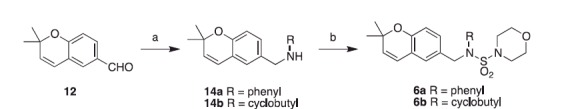
Synthesis of Class D compounds. Reagents and conditions: (a) aniline, InCl_3_, NaBH_4_, ACN, 20 min or cyclobutylamine, NaBH_4_, MeOH, overnight; (b) 4-morpholinosulfonyl chloride, pyridine, DCE, reflux 2 days.

Class D compounds were synthesised (Scheme 3) from **12** in two steps. First, **12** underwent reductive amination with either aniline or cyclobutylamine to give secondary amines **14a–b**. Next, the amines **14a–b** were reacted with 4-morpholinosulfonyl chloride to afford sulfonamides **6a–b**.

### Biology

All the analogs were assessed for their ability to inhibit the HIF-1 pathway using a luciferase reporter assay described previously^10^. This assay reports the ability for a compound to inhibit HIF transcriptional activity. However, it does not specifically reveal the mode of action at the biochemical level. As can be seen from Table 1, introduction of a morpholino unit on the sulfonamide nitrogen led to compounds (**5**) with substantially diminished activity. The same is true if the morpholino unit is directly attached to the sulfonyl group (**6**). In the two series of compounds (**3**, **4**) with a substituted phenyl group replacing the benzopyran ring in **1**, only introduction of the morpholino moiety at the para positions (**3**) allowed for the preservation of HIF inhibition activity. Indeed, compounds **3a** and **3d**, which have exchanged the benzopyran ring for a *para*-morpholinomethylphenyl and *para*-morpholinophenyl, respectively, exhibit IC_50_ values of 0.9 and 3.8 µM. Similarly, analogs **4a** and **4d**, which replace the *N*-phenyl with a *N*-cyclobutyl, but are otherwise structurally the same as **3a** and **3d**, have IC_50_ values of 1.0 and ∼2.6 µM, respectively. No other analogs synthesised in this work exhibited HIF inhibitory activity with IC_50_ lower than 5 µM, suggesting the importance of conserving electronic and/or steric effects para to the phenyl ring. In particular, compounds **3a** and **4a** are active within the same order of magnitude as **1**, and are about threefold less active than the previously discovered **2** (IC_50_ = ∼0.3 µM)^12^. The improved potency of **3a** and **4a** over **3d** and **4d** suggests a possible role for flexibility of the ligand in the binding site.

To gain some initial understanding of lipophilicity and solubility, the predicted log *D* and log *S* values were calculated for **1**, **2**, and their analogs. Log *P* refers to a molecule’s partition coefficient, or the log of the ratio between its solubility in octanol versus water^14^. This is commonly used to indicate a candidate drug’s lipophilicity, and a log *P* or cLog *P* (calculated log *P*) less than 5 is generally considered “drug-like”^15^. For ionizable small molecules, log *D* is the distribution constant, which describes the partition coefficient at different pH levels^16^. A molecule’s water solubility is typically measured at room temperature (20–25 °C) in mol/L and represented as log *S*, or clog *S* when calculated computationally. Drugs on the market with a variety of structures typically possess a log *S* between −5 and −2^17^.

Though several of the morpholine analogs have very drug-like properties, most are not active in the luciferase assay. Only **3a**, **3d**, **4a**, and **4d** are active toward the HIF pathway and only **3a** and **4a** show comparable IC_50_ values as **1**. Therefore, we examined their solubility in water and phosphate buffered saline (PBS).

### Solubility studies

To investigate the true enhancement of aqueous solubility, particle aggregation was examined using DLS. DLS can detect particle sizes in solution by measuring changes in scattered light in relation to the Brownian motion of particles^18^. It is commonly used to detect the particle sizes of various chemical and biological molecules, including small molecule inhibitors^19^. Though there are several methods for detecting solubility, we chose the DLS method due to its ease, reproducibility, minimal sample requirement, and relative sensitivity to small particles.

**Figure 3.**
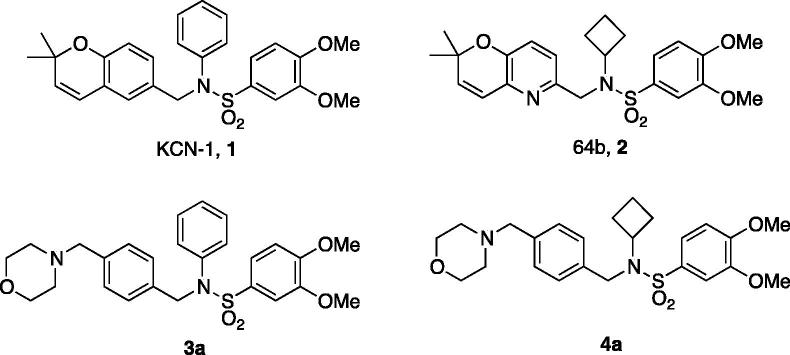
Structures of compounds used for dynamic light scattering.

The active compounds **3a** and **4a** were compared to their non-morpholine containing counterparts, **1** and **2**, respectively (Figure 3). Solutions of varying concentrations of each compound were made in either water or PBS with 1% DMSO. Each solution was measured in the particle size analyser to identify which samples showed formation of aggregates in solution. DLS measurements, summarised in Table 2, reveal that **3a** forms aggregates at approximately 10 µM, an order of magnitude higher than **1**, which is insoluble at just 1 µM in water. The *N*-cyclobutyl analog **4a** forms aggregates in excess of 100 µM, significantly higher than its counterpart **2**, which forms particles at a mere 10 µM. In PBS, the solubilities parallel those seen in the water solution, where **1** and **3a** exhibit comparable particle formation at 1 µM and 15 µM, respectively. **2** shows particle formation at 10 µM, while **4a** shows none at this concentration, as expected. Indeed, with a log *D* of 2.94 log *S* of −3.36 (Table 1), **4a** is predicted to be quite soluble in aqueous solutions.

**Table 2.** Measured solubility of selected compounds.

Name	Concentration of particle appearance in water (μM)	Concentration of particle appearance in PBS (μM)
**1**	1	1
**2**	10	10
**3a**	10	15
**4a**	>100	>50

The described results clearly indicate that (1) the benzopyran ring can be modified with minimal loss of activity and (2) the para position of the phenyl ring can tolerate substantial changes and can be used for improvement of water solubility. Such results will help future optimisation work.

## Conclusion

Of the 12 new morpholine-containing analogs developed in this work, four demonstrate HIF inhibition in the low or sub-micromolar range. In particular, **3a** and **4a** both exhibit inhibition of HIF transcriptional activity with IC_50_ values of 0.9 and 1.0 µM, respectively. As expected, the *in silico* log *P* and log *S* values of these analogs are considered more favourable than lead compound **1** or its more potent analog **2**, and are therefore likely to be more bioavailable. Following these indications, solubility as measured by particle detection with DLS reveal the exceptional solubility of analogs **3a** and **4a** over their non-morpholine containing predecessors **1** and **2**. Particle formation of **4a** is undetected in excess of 100 µM in water and 50 µM in PBS, while still displaying HIF inhibition in the same order of magnitude as lead **1**. These results encourage exploration and use of more soluble moieties to further probe the SAR (structure–activity relationship) and SSR (structure–solubility relationship) of potential analogs.

## Supplementary Material

IENZ_1347784_Supplementary_Material.pdf
