# Supplementary material for: Design and synthesis of benzopyran-based inhibitors of the hypoxia-inducible factor-1 pathway with improved water solubility
Source: J Enzyme Inhib Med Chem. 2017 Aug 2;32(1):992–1001. doi: 10.1080/14756366.2017.1347784 (PMC6009948; doi:10.1080/14756366.2017.1347784)

Supplemental Material For

Design and Synthesis of Benzopyran-based Inhibitors of Hypoxia-Inducible Factor-1 Pathway with Improved Water Solubility

Jalisa H. Ferguson, Zeus De Los Santos, Saroja N. Devi, Stefan Kaluz, Erwin Van Meir, Sarah K. Zingales, Binghe Wang.

Contents

Structural Characterization of Compounds **3a-14b** Pp. 2-58

Graphical Representations of calculated log*D* and log*S* values for **3a-6b** Pp. 59-70

3,4-Dimethoxy-*N*-(4-(morpholinomethyl)benzyl)-*N*-phenylbenzenesulfonamide (3a)

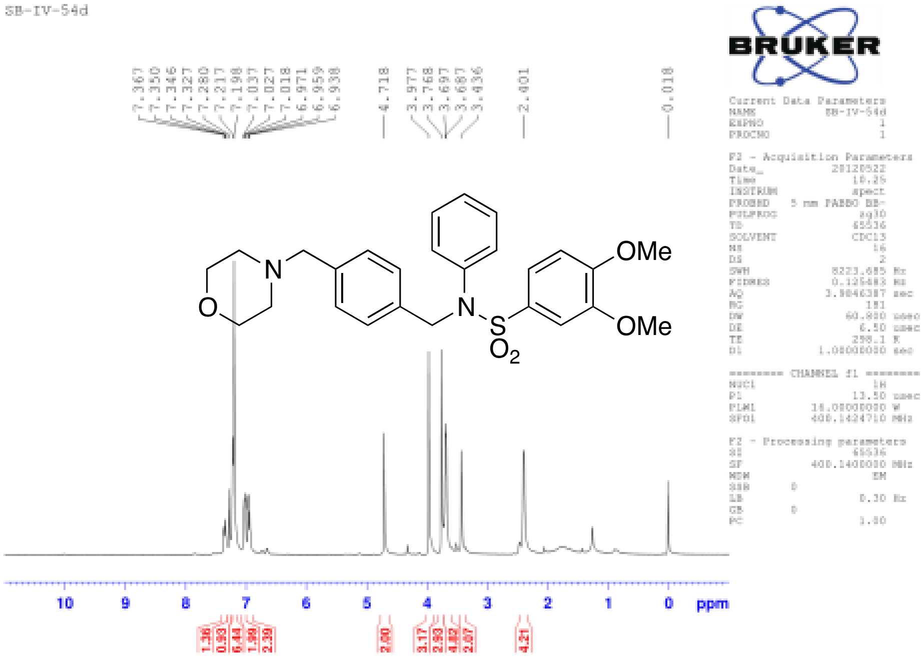

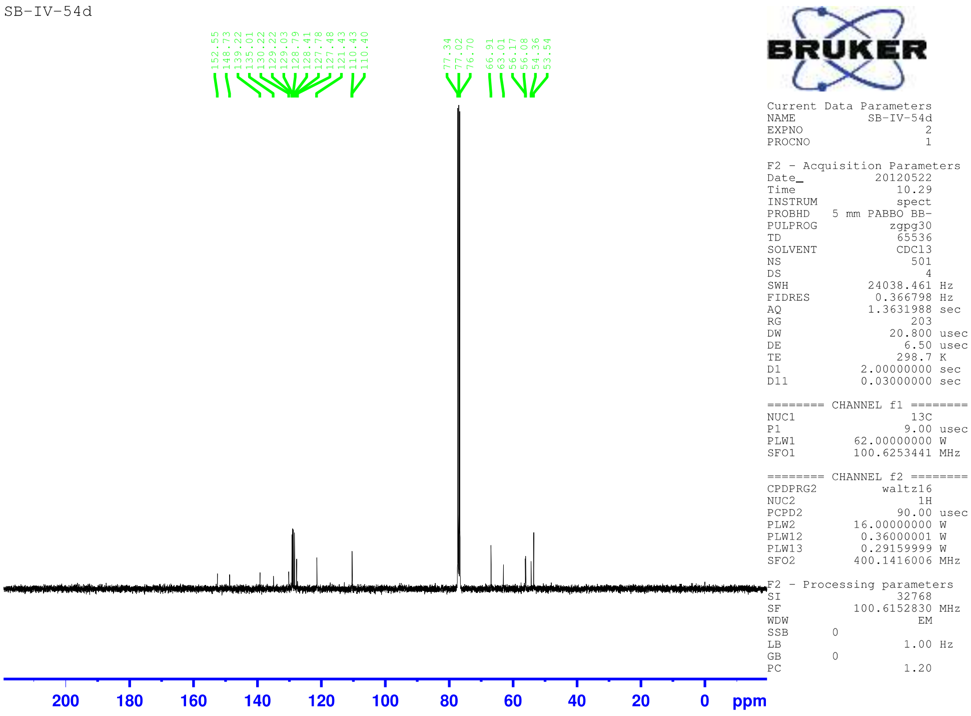

100%MeOH **11:09:27 23-May-2012**

SARAH_SB-IV-54d_BWANG-ACCU_05-23-2012_ESI-POS02 37 (0.686) AM (Cen,4, 80.00, Ar,5000.0,556.28,0.70); Cm

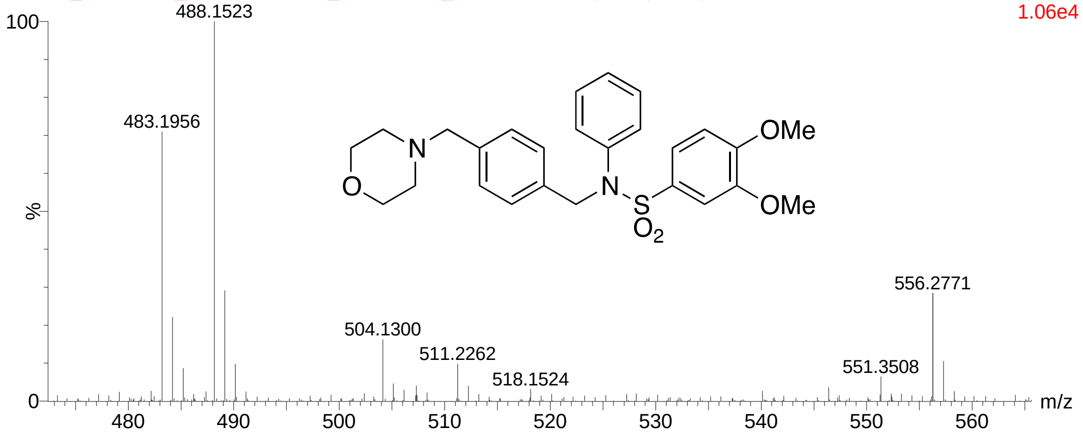

Elemental Composition Report

Single Mass Analysis
Tolerance = 20.0 PPM / DBE: min = -1.5, max = 50.0
Element prediction: Off
Number of isotope peaks used for i-FIT = 3

Monoisotopic Mass, Even Electron Ions
6053 formula(e) evaluated with 64 results within limits (all results (up to 1000) for each mass)
Elements Used:
C: 1-200 H: 1-200 N: 1-15 O: 1-100 S: 0-50

Minimum:				-1.5			
Maximum:		5.0	20.0	50.0			
Mass	Calc. Mass	mDa		PPM	DBE	i-FIT	Formula
483.1956	483.1954	0.2	0.4	12.5	12.4		C26 H31 N2 O5 S

3,4-Dimethoxy-*N*-(3-(morpholinomethyl)benzyl)-N-phenylbenzenesulfonamide (3b)

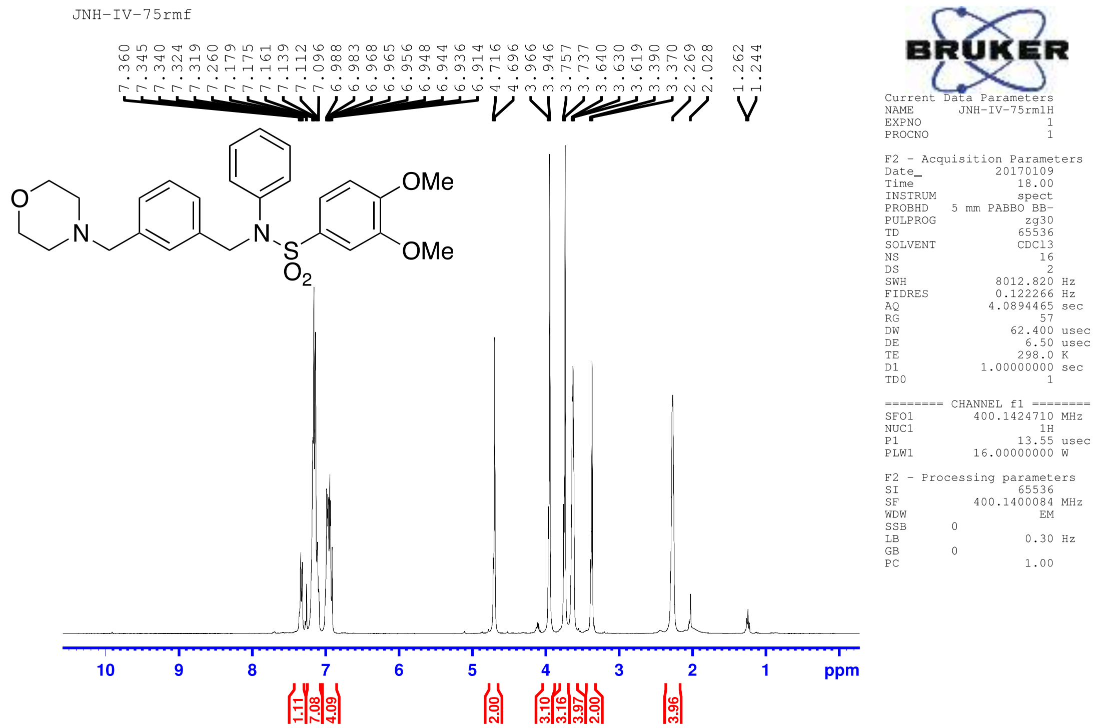

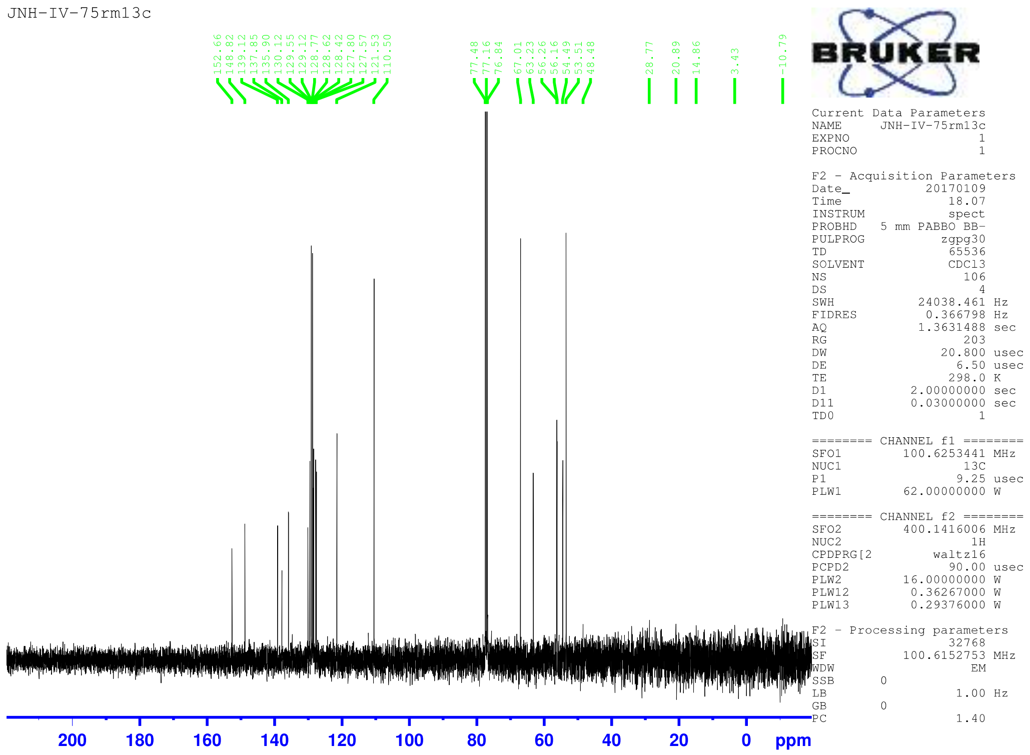

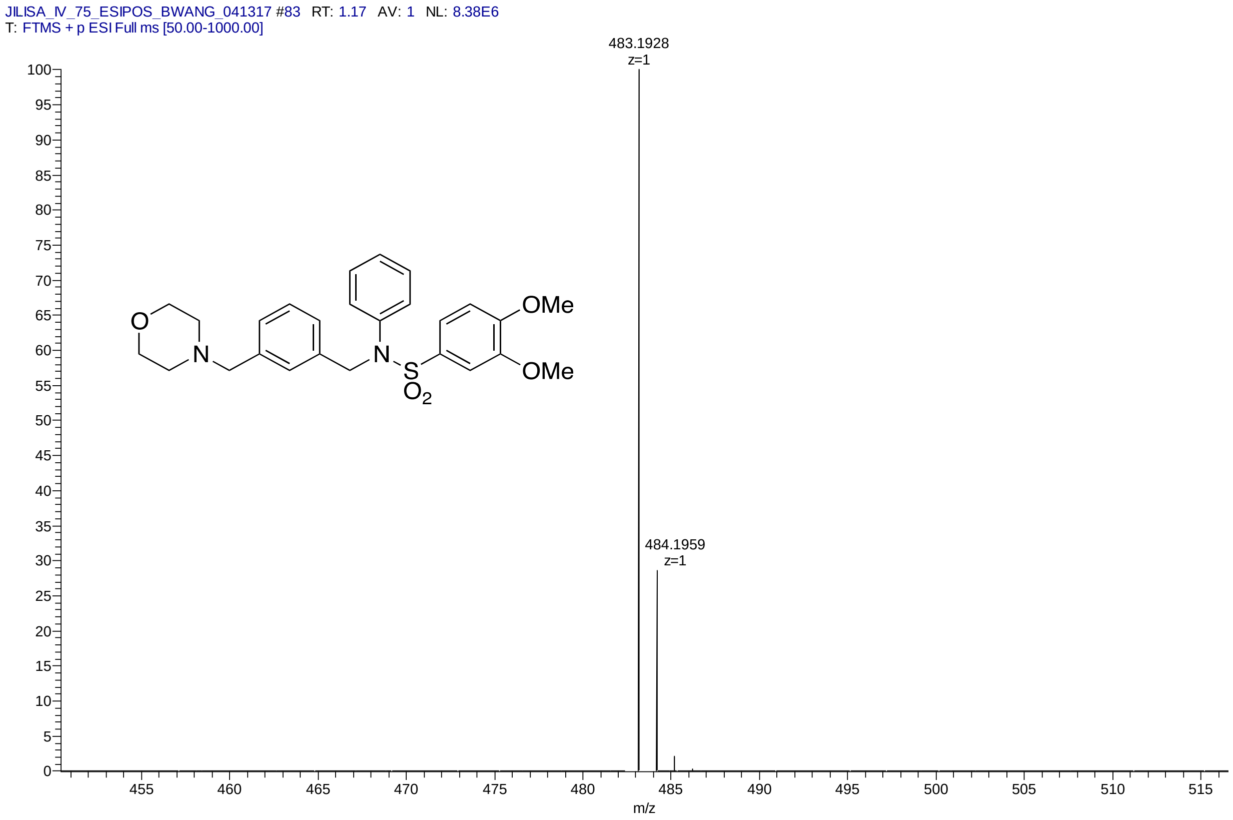
JILISA_IV_75_ESIPOS_BWANG_041317 #83 RT: 1.17 AV: 1 NL: 8.38E6
T: FTMS + p ESI Full ms [50.00-1000.00]
483.1928
z=1
484.1959
z=1
OMe
OMe
O
N
N
S
O2
m/z

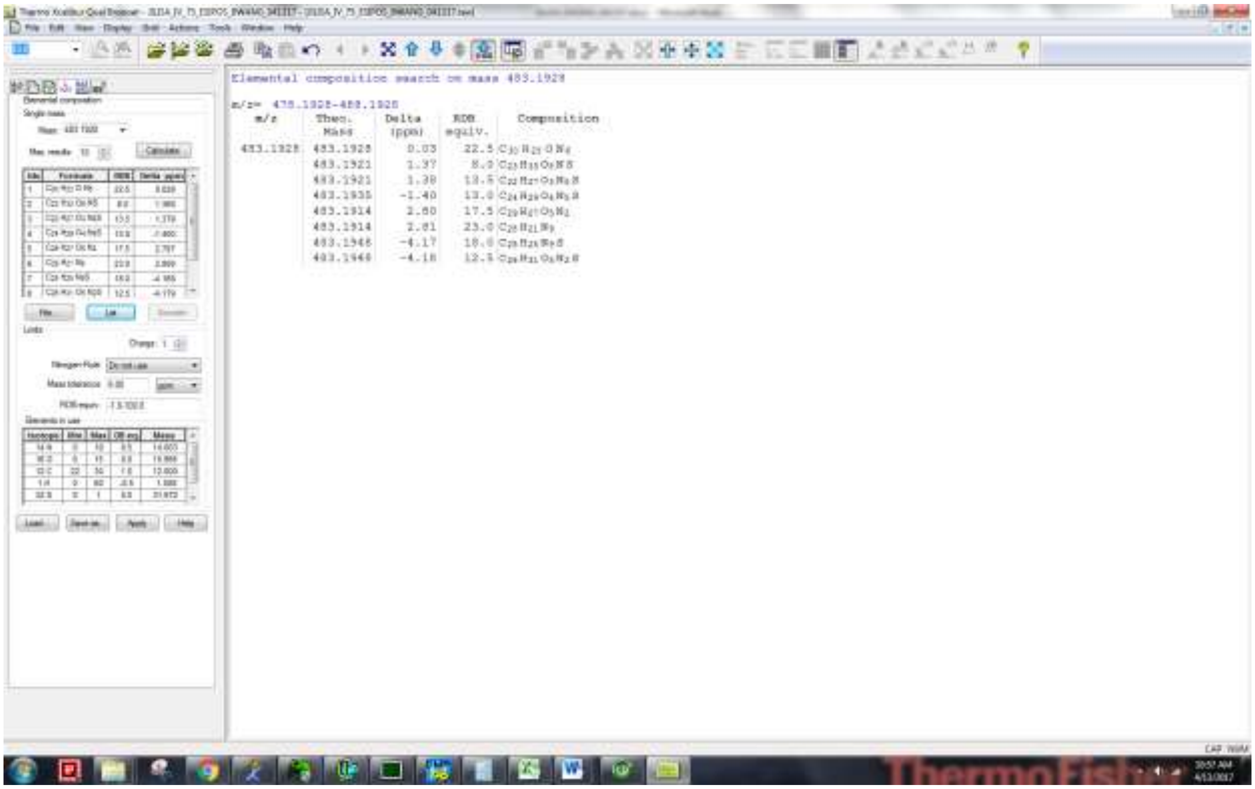
Elemental composition search on mass 483.1928
m/z= 478.1928-488.1928
m/z
Theo. Mass
Delta (ppm)
RDB equiv.
Composition
483.1928
483.1928 0.03 22.5
483.1921 1.37 8.0
483.1921 1.38 13.5
483.1935 -1.40 13.0
483.1914 2.80 17.5
483.1914 2.81 23.0
483.1948 -4.17 18.0
483.1948 -4.18 12.5

3,4-Dimethoxy-*N*-(2-(morpholinomethyl)benzyl)-*N*-phenylbenzenesulfonamide (3c)

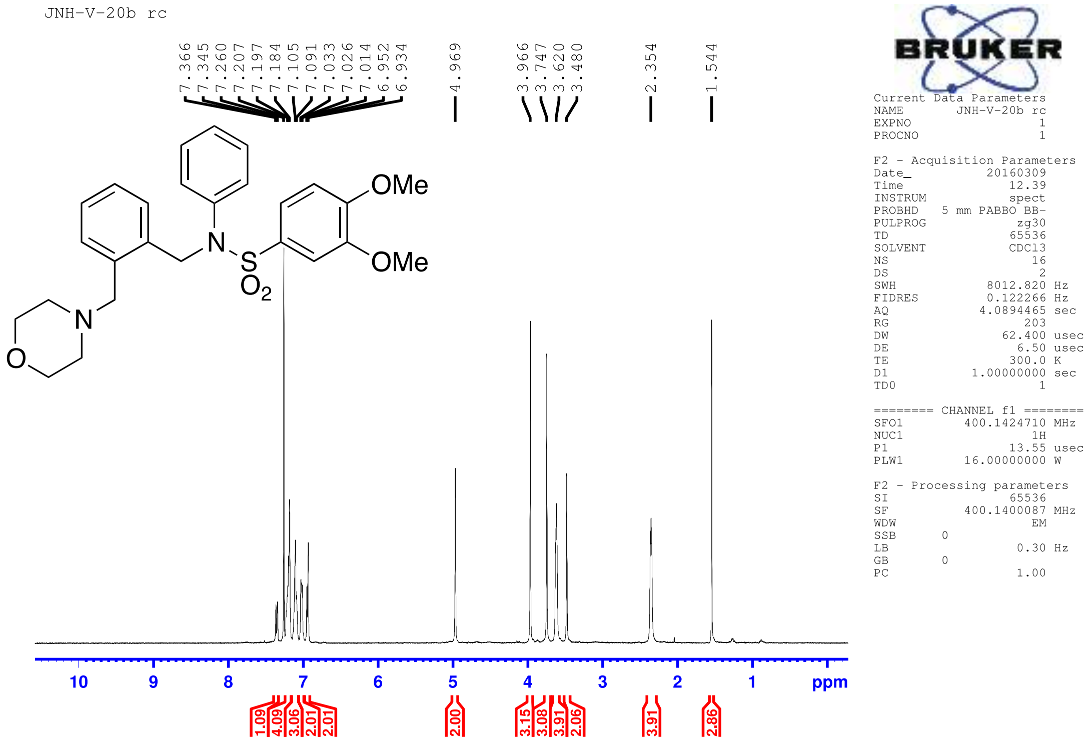

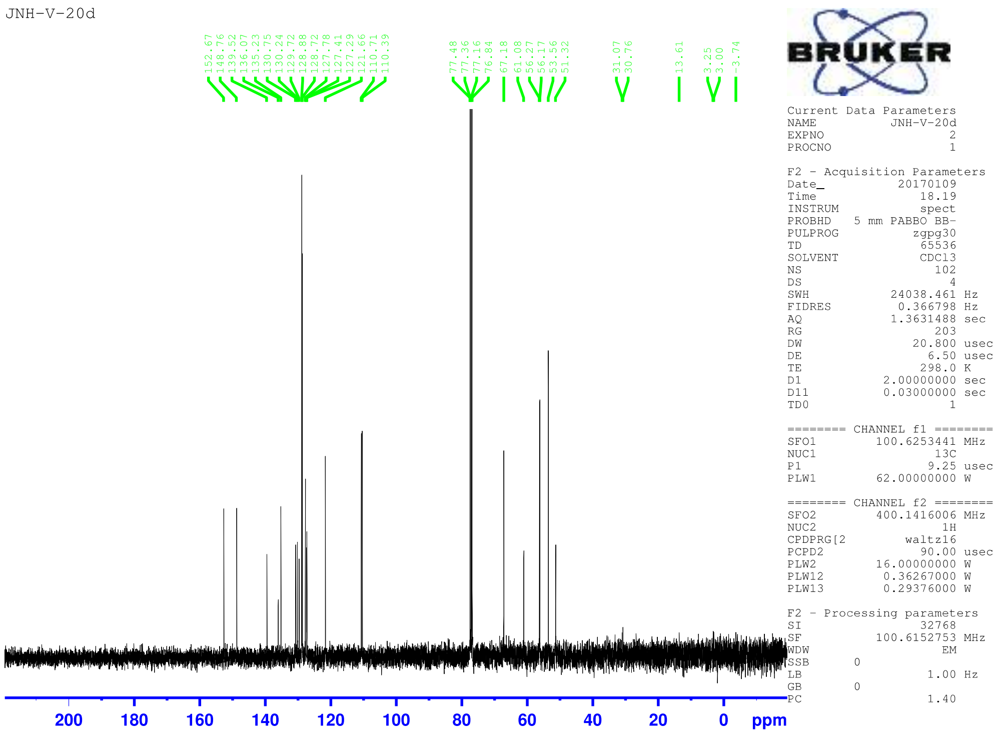

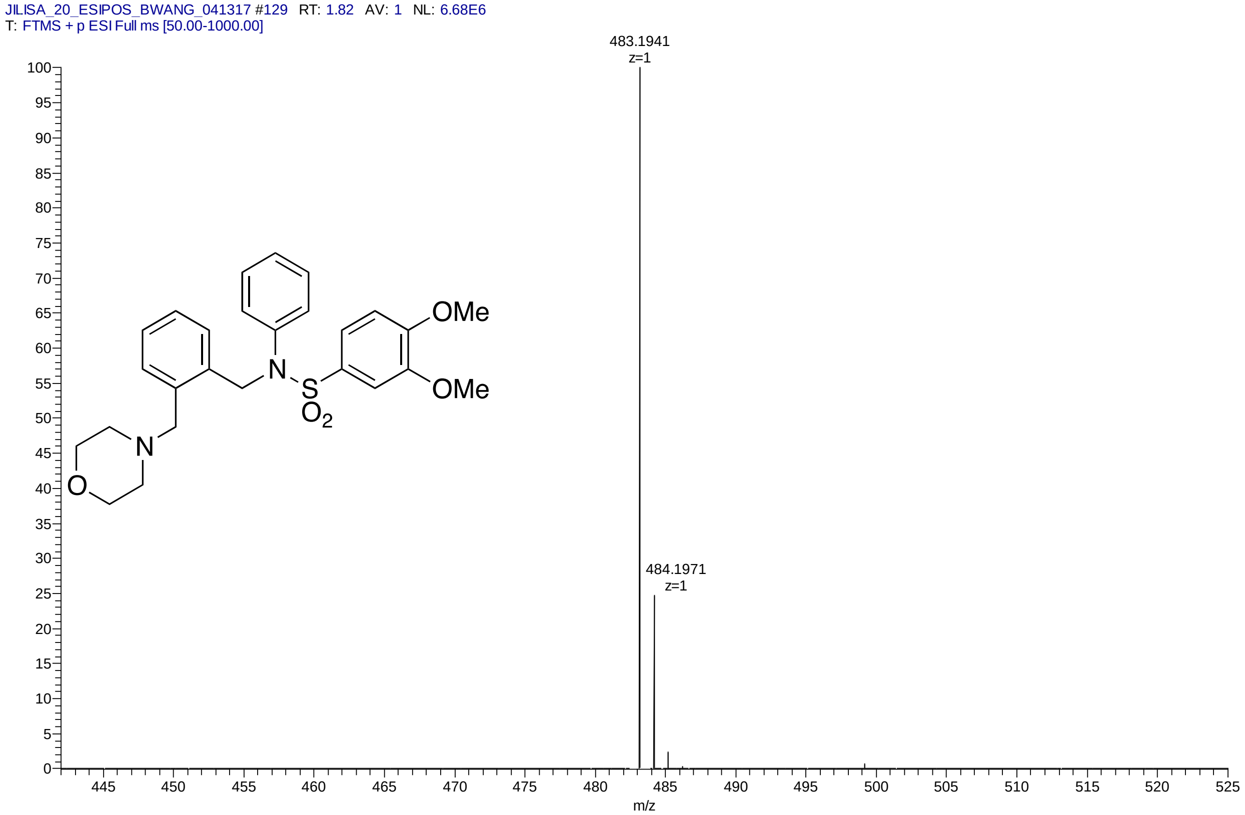
JILISA_20_ESIPOS_BWANG_041317 #129 RT: 1.82 AV: 1 NL: 6.68E6
T: FTMS + p ESI Full ms [50.00-1000.00]
483.1941
z=1
484.1971
z=1
m/z
OMe
OMe
N
S
O2
N
O

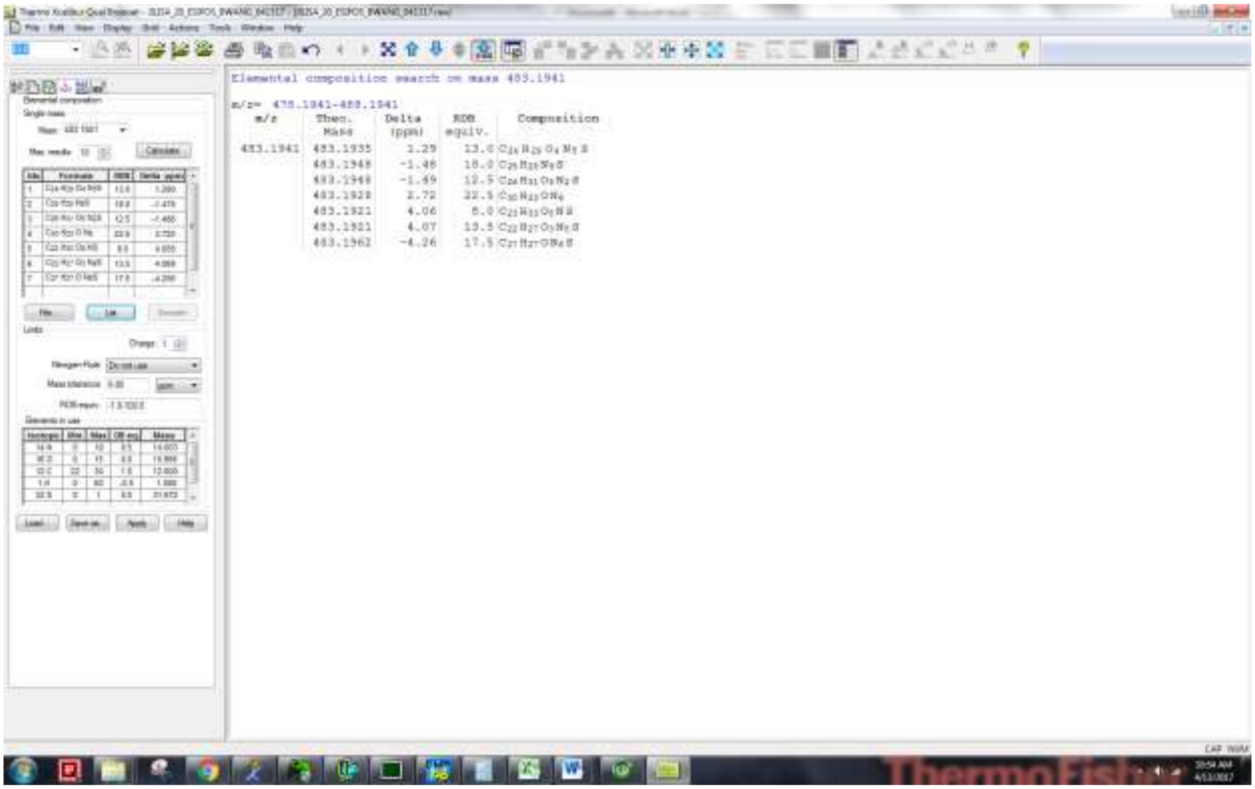
Elemental composition search on mass 483.1941
m/z Theo. Mass Delta (ppm) RDB equiv. Composition
483.1941 483.1935 1.29 13.0
483.1948 -1.48 18.0
483.1948 -1.49 12.5
483.1928 2.72 22.5
483.1921 4.06 8.0
483.1921 4.07 13.5
483.1962 -4.26 17.5

3,4-Dimethoxy-*N*-(4-morpholinobenzyl)-*N*-phenylbenzenesulfonamide (3d)

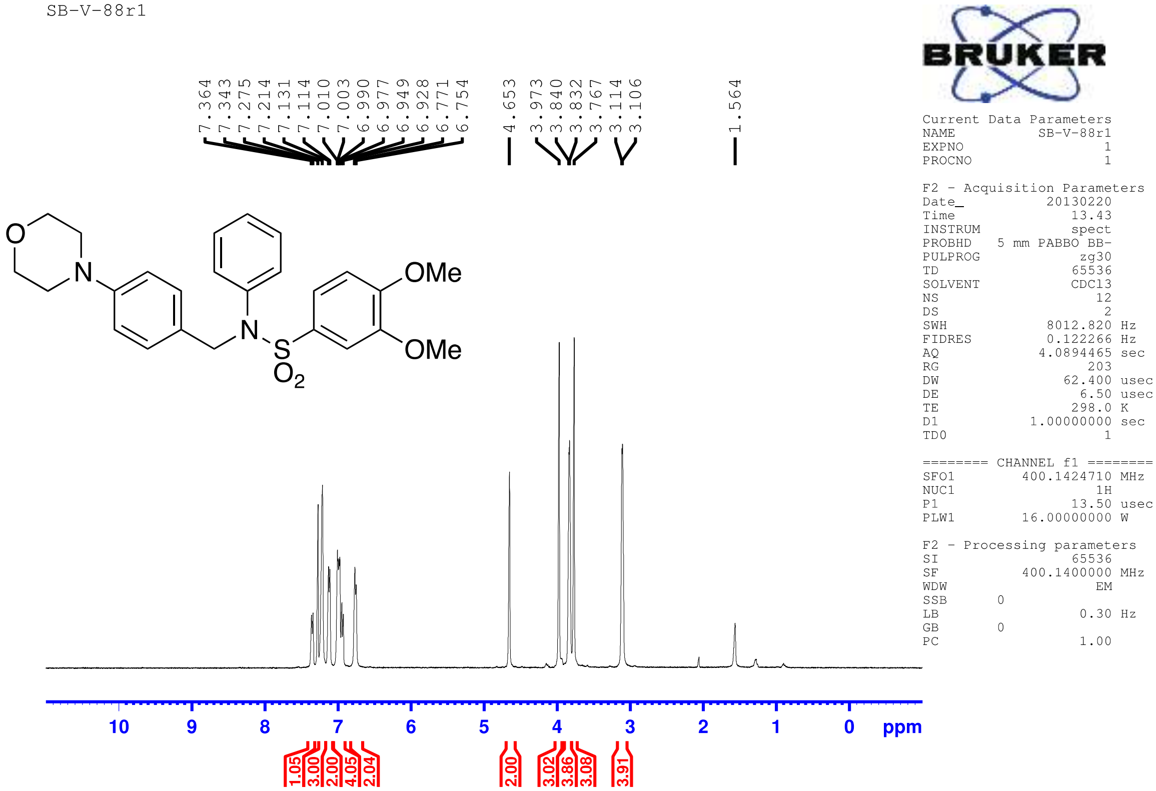

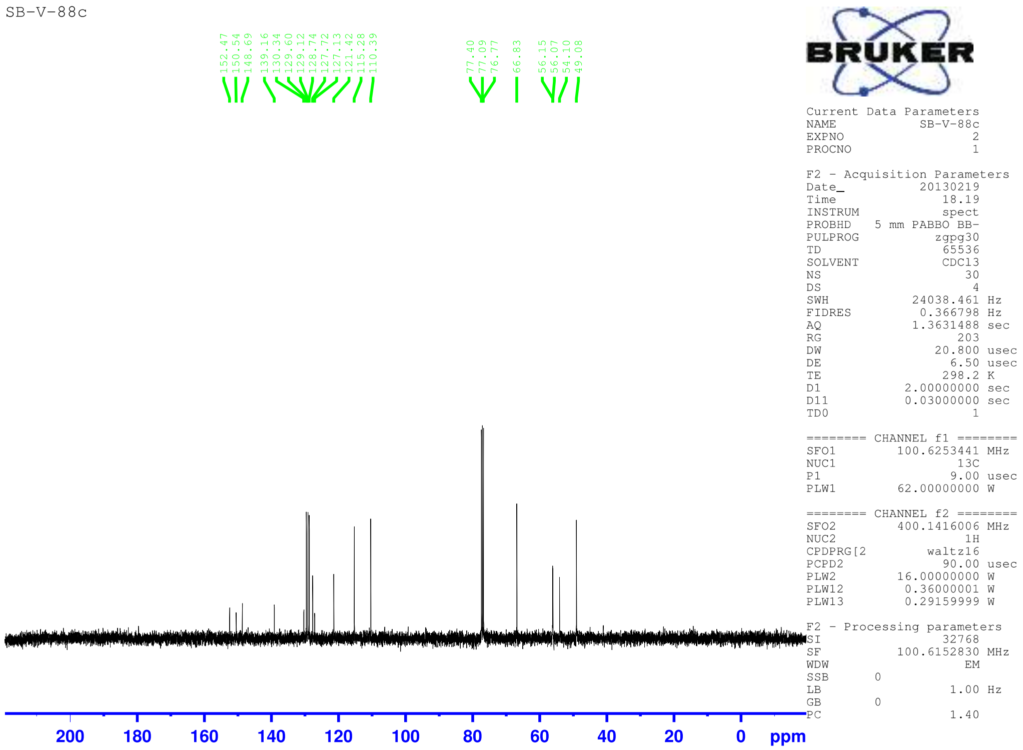

MeOH 15:07:31 26-Feb-2013

SARAH_SB-V-88_BWANG-ACCU_02-26-2013_ESI-POS02 30 (0.597) AM (Cen,2, 80.00, Ar,5000.0,556.28,0.70); Sm (SG, 3x

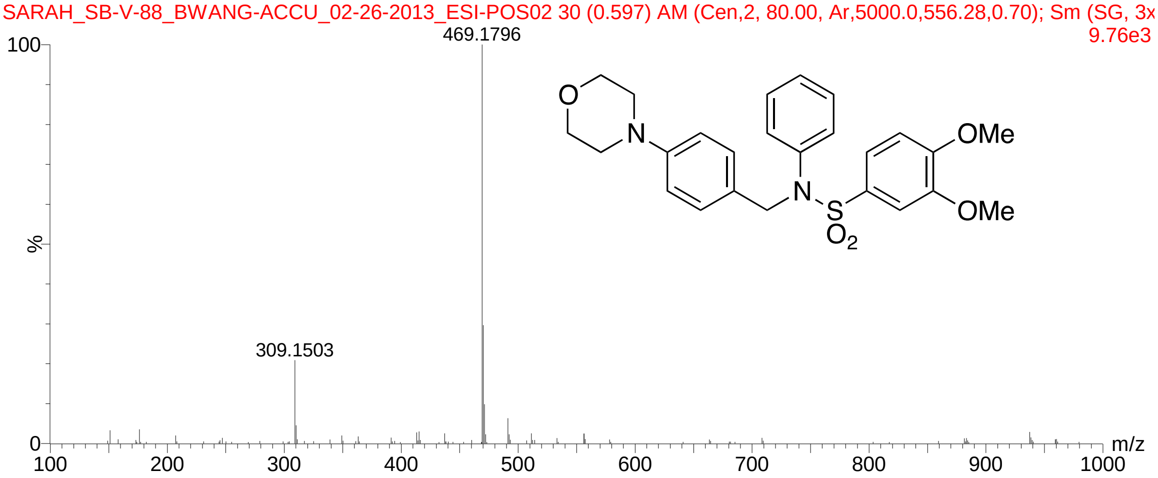

Elemental Composition Report

Single Mass Analysis
Tolerance = 5.0 PPM / DBE: min = -1.5, max = 50.0
Element prediction: Off
Number of isotope peaks used for i-FIT = 3

Monoisotopic Mass, Even Electron Ions
2331 formula(e) evaluated with 5 results within limits (up to 100 closest results for each mass)
Elements Used:
C: 1-150 H: 1-150 N: 1-6 O: 1-30 S: 1-10

Minimum:				-1.5			
Maximum:		5.0	5.0	50.0			
Mass	Calc. Mass	mDa	PPM	DBE	i-FIT	Formula	
469.1796	469.1797	-0.1	-0.2	12.5	0.3	C25 H29 N2 O5 S	

***N*-Cyclobutyl-3,4-dimethoxy-*N*-(4-(morpholinomethyl)benzyl)benzenesulfonamide (4a)**

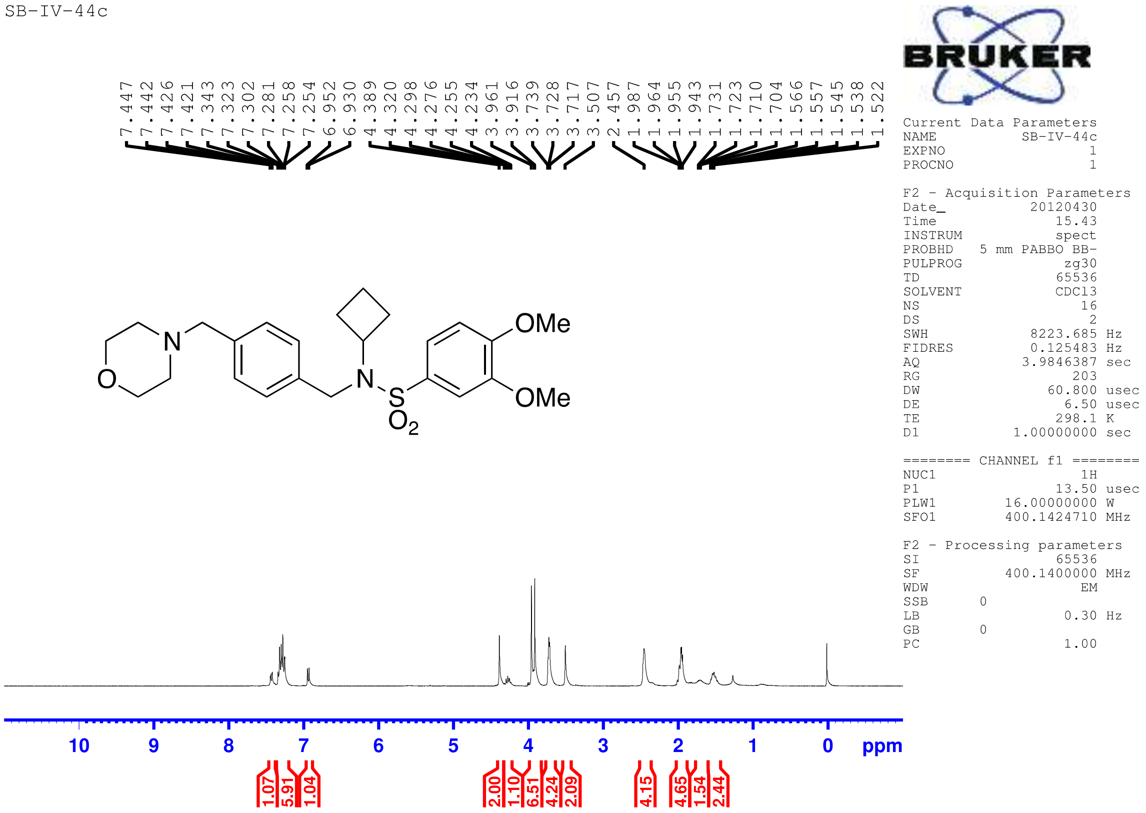

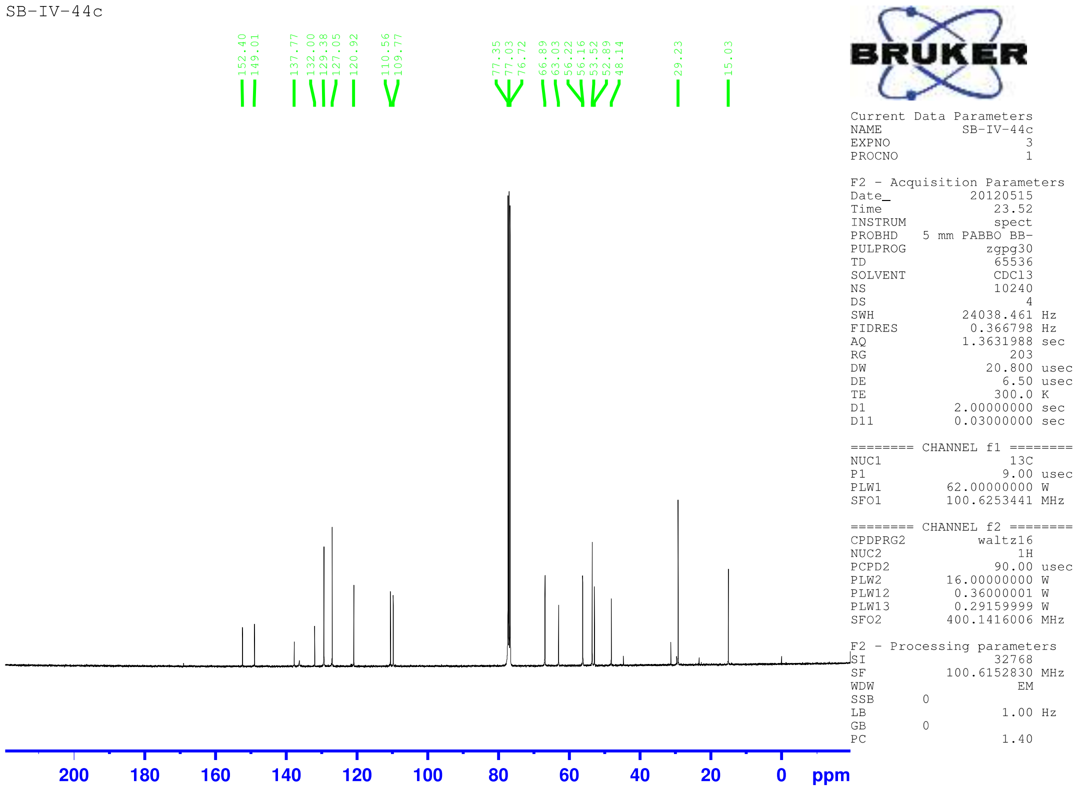

100%MeOH+0.1%HCOOH 12:30:52 17-May-2012

SARAH_SB-IV-44C_BWANG-ACCU_05-17-2012_ESI-POS02 80 (1.490) AM (Cen,4, 80.00, Ar,5000.0,556.28,0.70); Cm

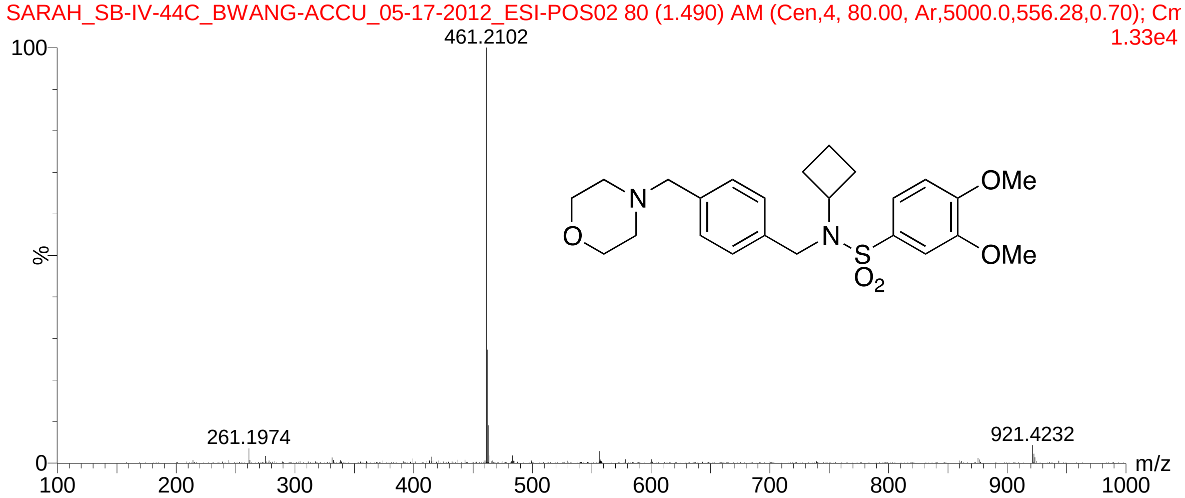

Elemental Composition Report

Single Mass Analysis
Tolerance = 5.0 PPM / DBE: min = -1.5, max = 50.0
Element prediction: Off
Number of isotope peaks used for i-FIT = 3

Monoisotopic Mass, Even Electron Ions
4674 formula(e) evaluated with 14 results within limits (all results (up to 1000) for each mass)
Elements Used:
C: 1-100 H: 1-100 N: 1-15 O: 1-100 S: 0-6

Minimum:				-1.5			
Maximum:		5.0	5.0	50.0			
Mass	Calc. Mass	mDa	PPM	DBE	i-FIT		Formula
461.2102	461.2110	-0.8	-1.7	9.5	5.9		C24 H33 N2 O5 S

N-Cyclobutyl-3,4-dimethoxy-*N*-(3-(morpholinomethyl)benzyl)benzenesulfonamide (4b)

SB-V-92f

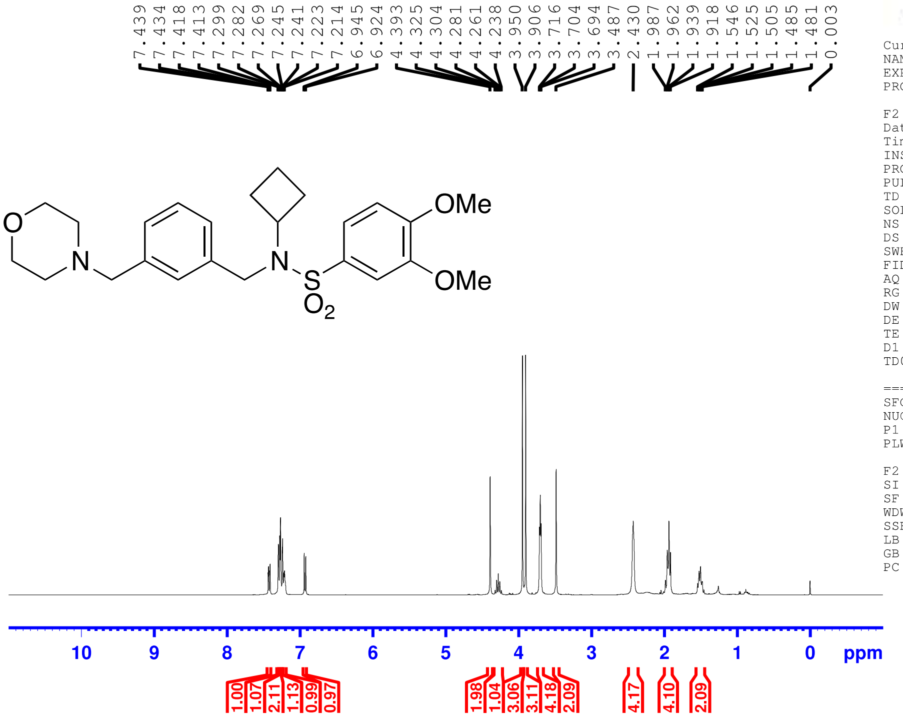


```
Current Data Parameters
NAME            SB-V-92f
EXPNO                  1
PROCNO                 1

F2 - Acquisition Parameters
Date_           20130312
Time               20.47
INSTRUM            spect
PROBHD   5 mm PABBO BB-
PULPROG             zg30
TD                 65536
SOLVENT            CDCl3
NS                    16
DS                     2
SWH             8012.820 Hz
FIDRES          0.122266 Hz
AQ             4.0894465 sec
RG                    57
DW                62.400 usec
DE                  6.50 usec
TE                 293.7 K
D1            1.00000000 sec
TD0                    1

======== CHANNEL f1 ========
SFO1         400.1424710 MHz
NUC1                  1H
P1                 13.50 usec
PLW1         16.00000000 W

F2 - Processing parameters
SI                 65536
SF           400.1400000 MHz
WDW                   EM
SSB      0
LB                  0.30 Hz
GB       0
PC                  1.00
```

SB-V-92f

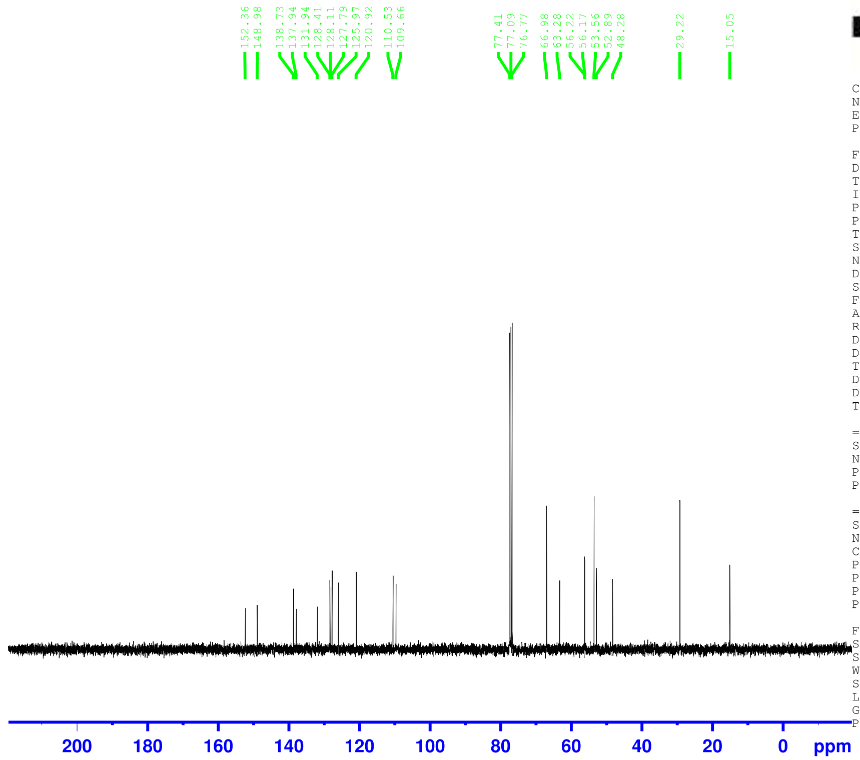


```
Current Data Parameters
NAME            SB-V-92f
EXPNO                  2
PROCNO                 1

F2 - Acquisition Parameters
Date_           20130312
Time               20.51
INSTRUM            spect
PROBHD   5 mm PABBO BB-
PULPROG           zgpg30
TD                 65536
SOLVENT            CDCl3
NS                    30
DS                     4
SWH            24038.461 Hz
FIDRES          0.366798 Hz
AQ             1.3631488 sec
RG                   203
DW                20.800 usec
DE                  6.50 usec
TE                 294.4 K
D1            2.00000000 sec
D11           0.03000000 sec
TD0                    1

======== CHANNEL f1 ========
SFO1         100.6253441 MHz
NUC1                 13C
P1                  9.00 usec
PLW1         62.00000000 W

======== CHANNEL f2 ========
SFO2         400.1416006 MHz
NUC2                  1H
CPDPRG[2         waltz16
PCPD2              90.00 usec
PLW2         16.00000000 W
PLW12         0.36000001 W
PLW13         0.29159999 W

F2 - Processing parameters
SI                 32768
SF           100.6152830 MHz
WDW                   EM
SSB      0
LB                  1.00 Hz
GB       0
PC                  1.40
```

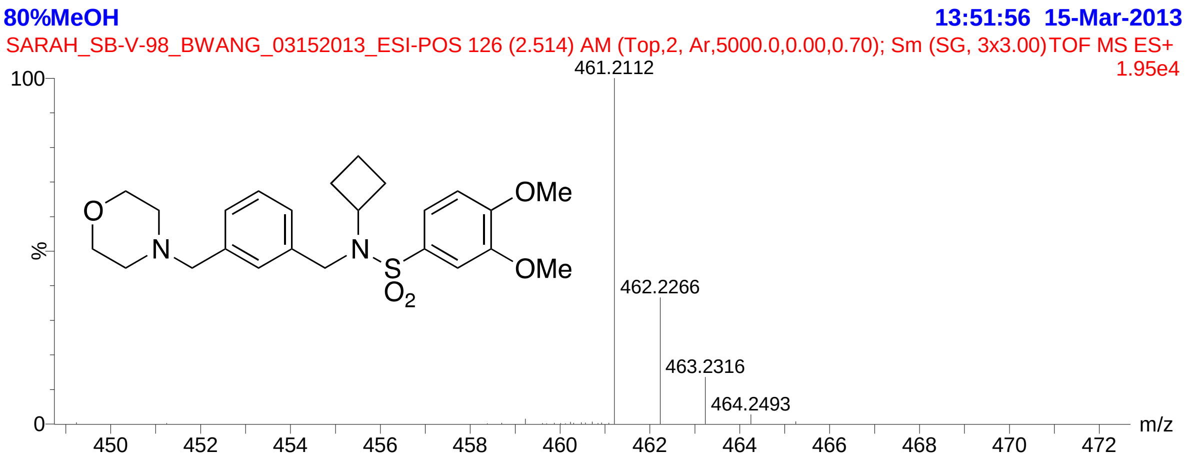

Elemental Composition Report

Single Mass Analysis
Tolerance = 5.0 PPM / DBE: min = -1.5, max = 50.0
Element prediction: Off
Number of isotope peaks used for i-FIT = 3

Monoisotopic Mass, Even Electron Ions
4668 formula(e) evaluated with 10 results within limits (all results (up to 1000) for each mass)
Elements Used:
C: 1-150 H: 1-150 N: 1-30 O: 1-60 S: 1-10

Minimum:				-1.5		
Maximum:		5.0	5.0	50.0		
Mass	Calc. Mass	mDa	PPM	DBE	i-FIT	Formula
461.2112	461.2110	0.2	0.4	9.5	243.0	C24 H33 N2 O5 S

N-Cyclobutyl-3,4-dimethoxy-*N*-(2-(morpholinomethyl)benzyl)benzenesulfonamide (4c)

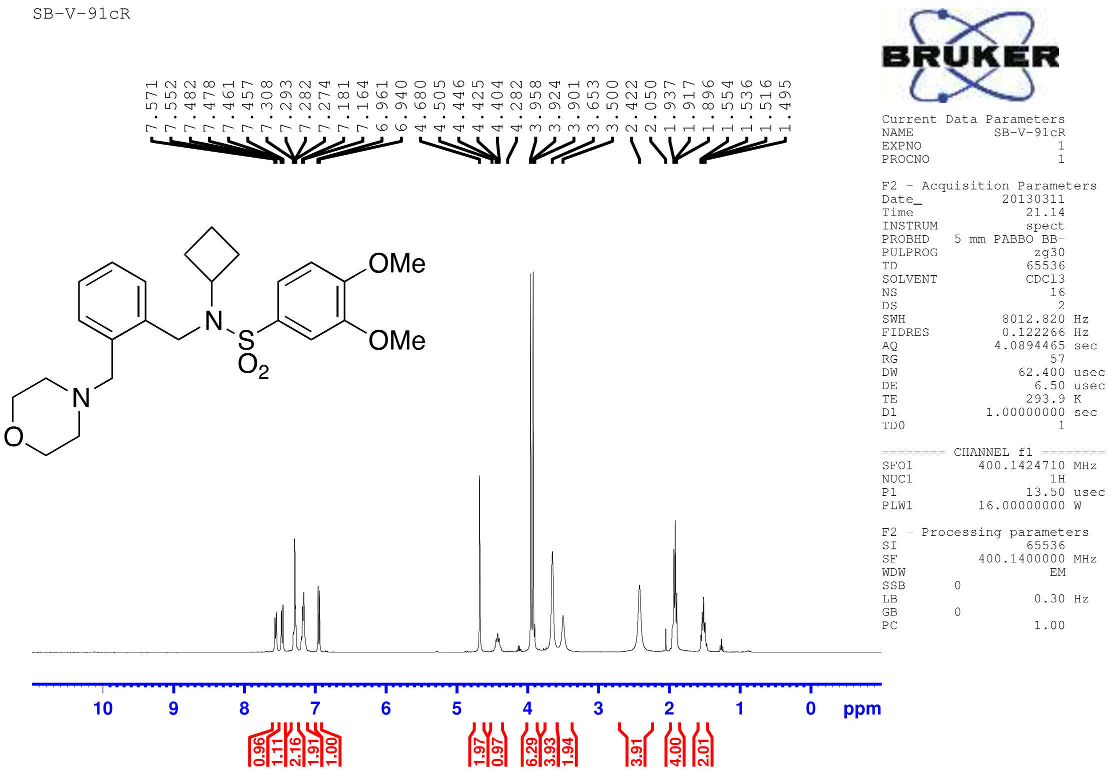

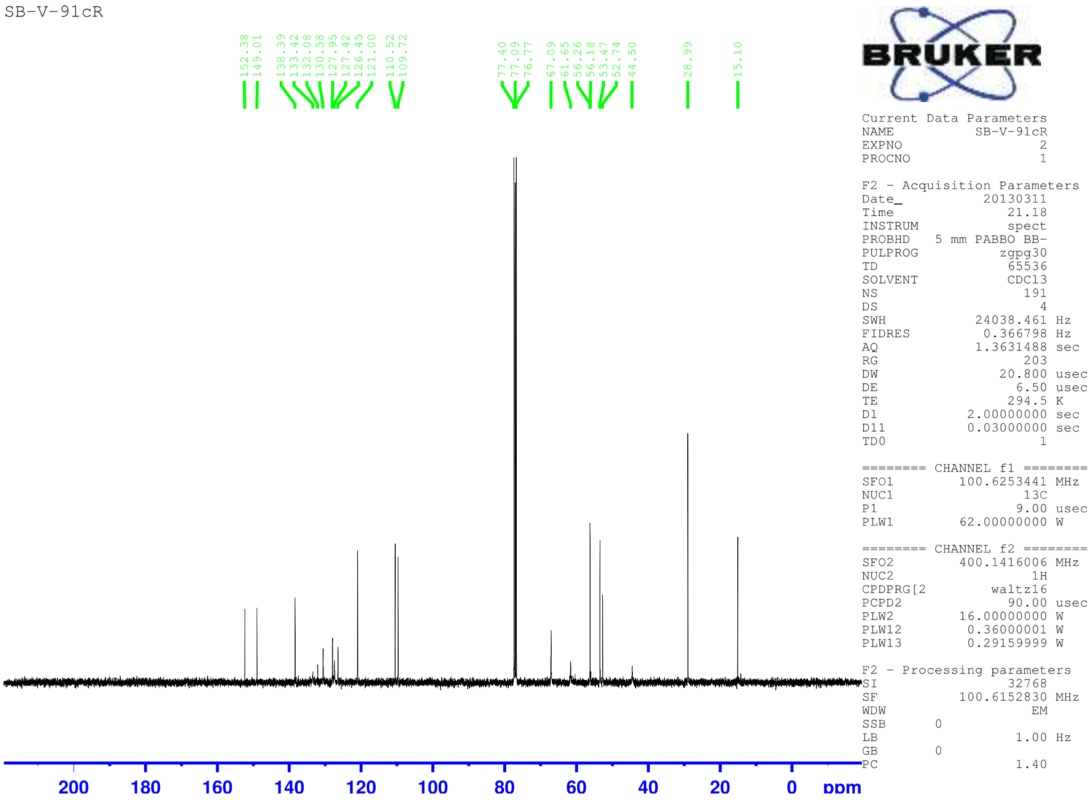

diluted in 50%ACN **16:22:35 25-Mar-2013**

SARAH_SB-V-97C_BWANG-ACCU_03152013_ESI-POS03 76 (1.512) AM (Cen,2, 80.00, Ar,5000.0,556.28,0.70); Sm (SG, 3x

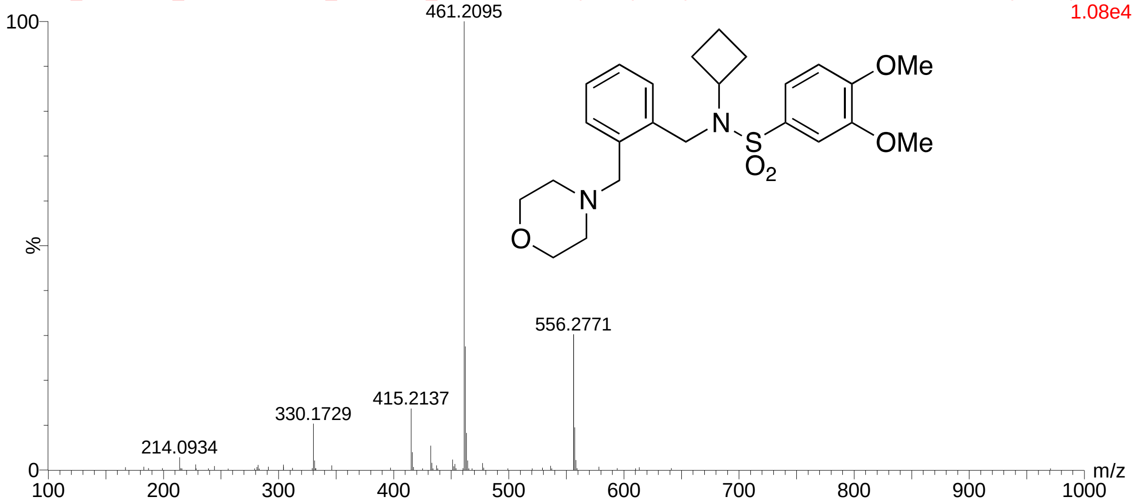

Elemental Composition Report

Single Mass Analysis
Tolerance = 5.0 PPM / DBE: min = -1.5, max = 50.0
Element prediction: Off
Number of isotope peaks used for i-FIT = 3

Monoisotopic Mass, Odd and Even Electron Ions
4668 formula(e) evaluated with 21 results within limits (up to 100 closest results for each mass)
Elements Used:
C: 1-150 H: 1-150 N: 1-30 O: 1-60 S: 1-10

Minimum:				-1.5		
Maximum:		5.0	5.0	50.0		
Mass	Calc. Mass	mDa	PPM	DBE	i-FIT	Formula
461.2095	461.2110	-1.5	-3.3	9.5	11.1	C24 H33 N2 O5 S

***N*-Cyclobutyl-3,4-dimethoxy-N-(4-morpholinobenzyl)benzenesulfonamide (4d)**

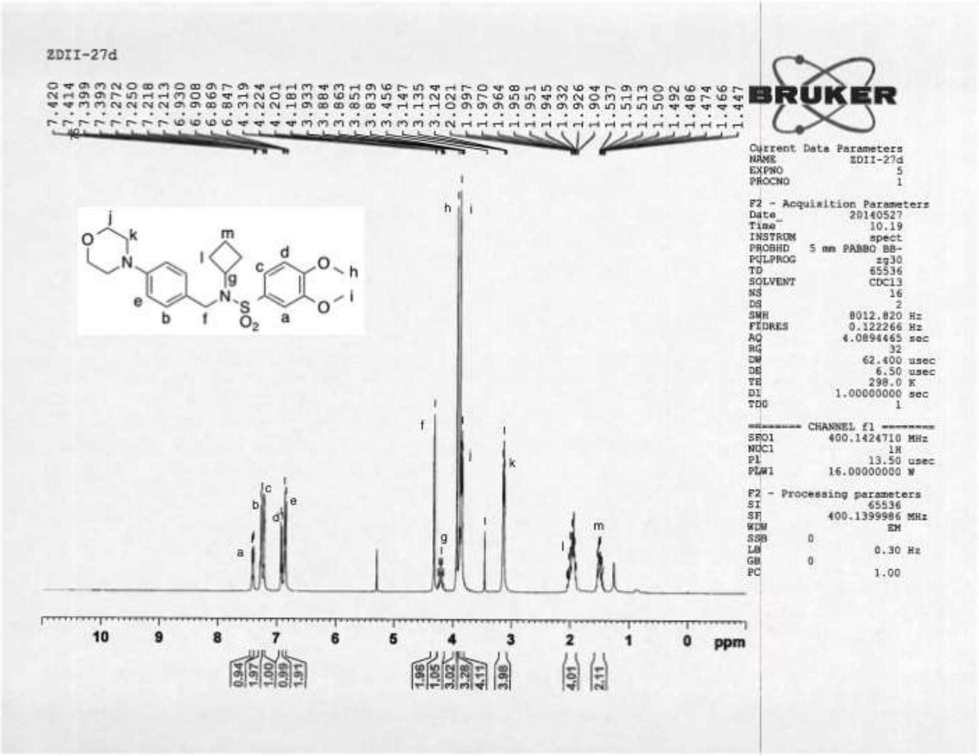

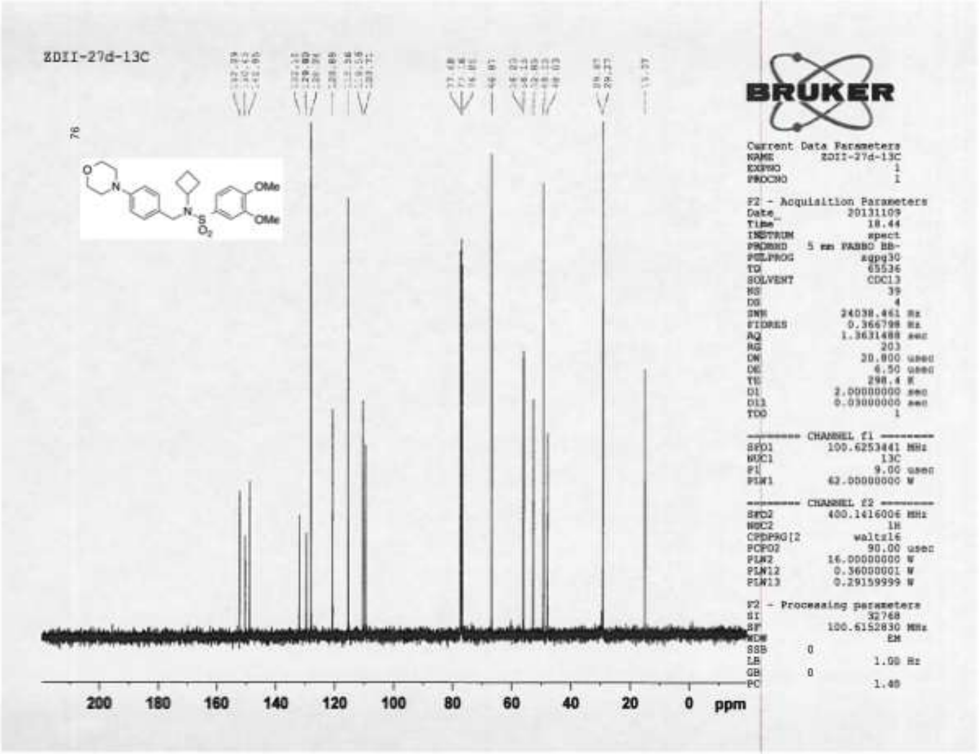

***N*-((2,2-Dimethyl-2H-chromen-6-yl)methyl)-3,4-dimethoxy-N-(2-morpholinoethyl)benzenesulfonamide (5a)**

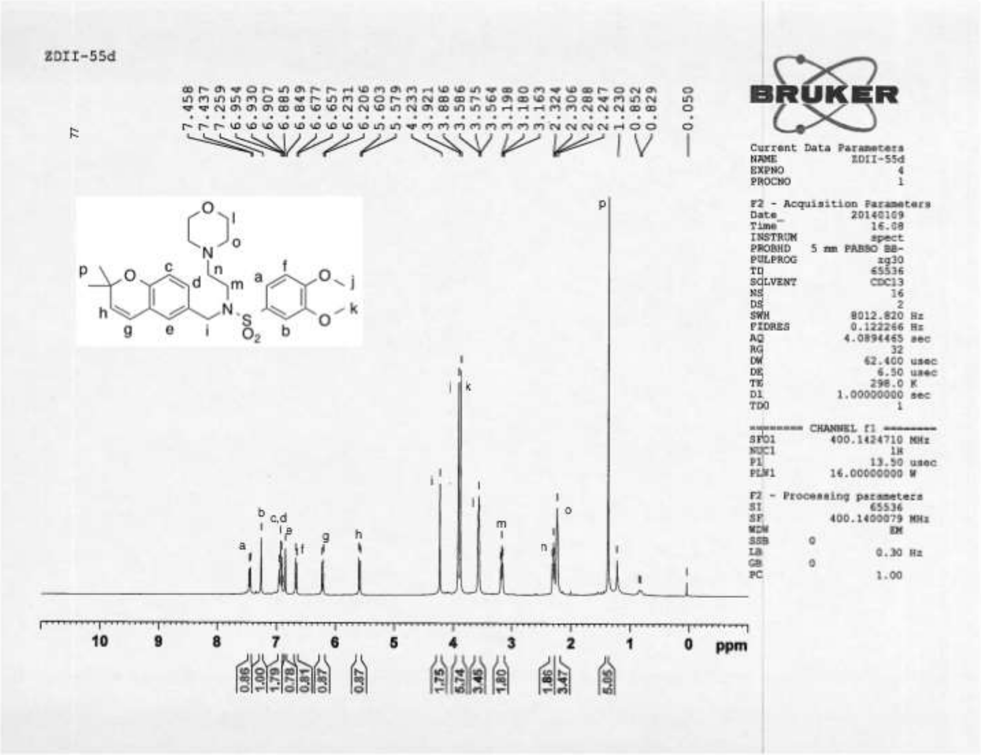

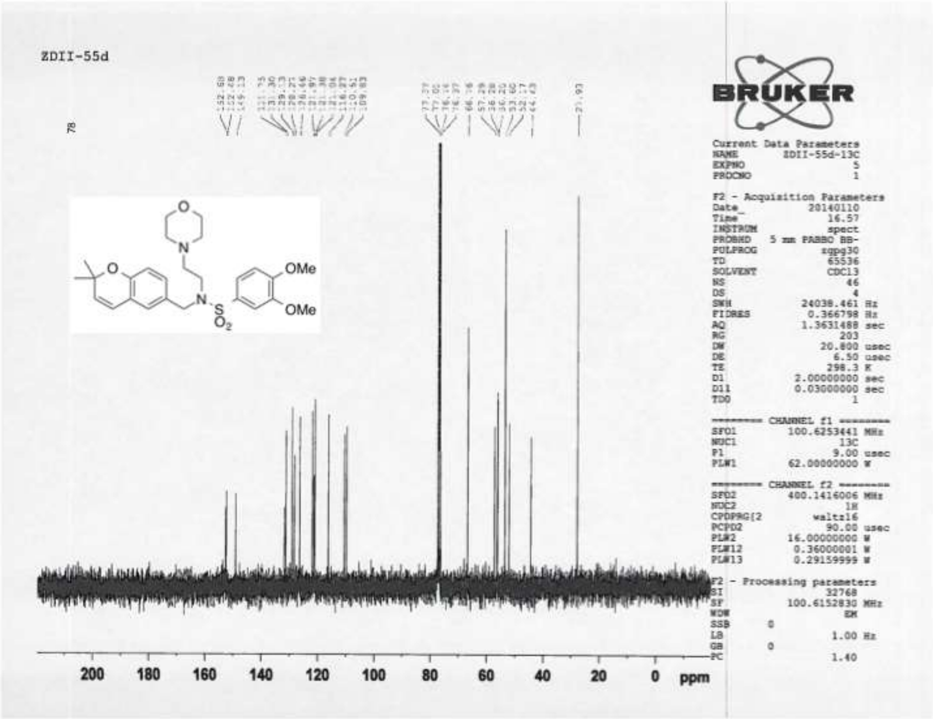

***N*-((2,2-Dimethyl-2H-chromen-6-yl)methyl)-3,4-dimethoxy-N-(3-morpholinopropyl)benzenesulfonamide (5b)**

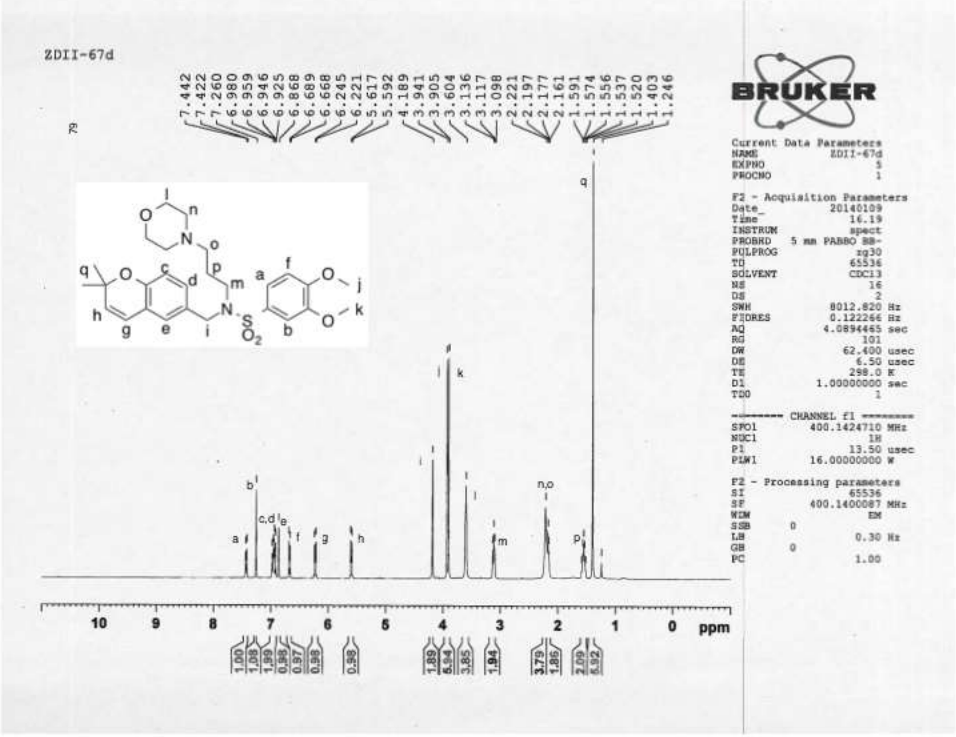

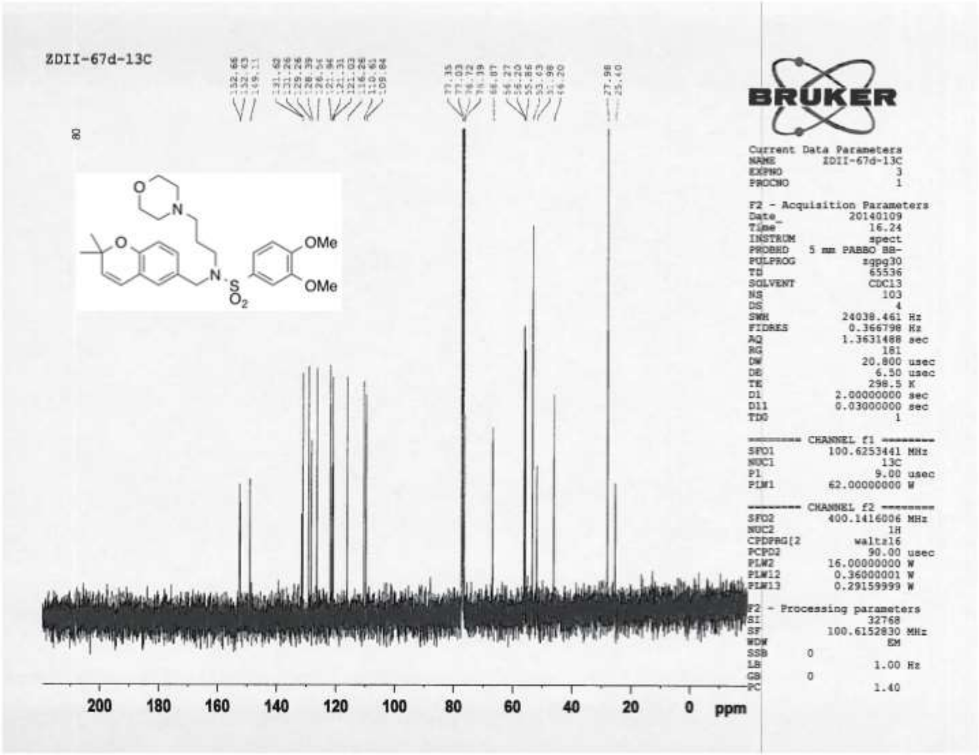

N-((2,2-Dimethyl-2*H*-chromen-6-yl)methyl)-*N*-phenylmorpholine-4-sulfonamide (6a)

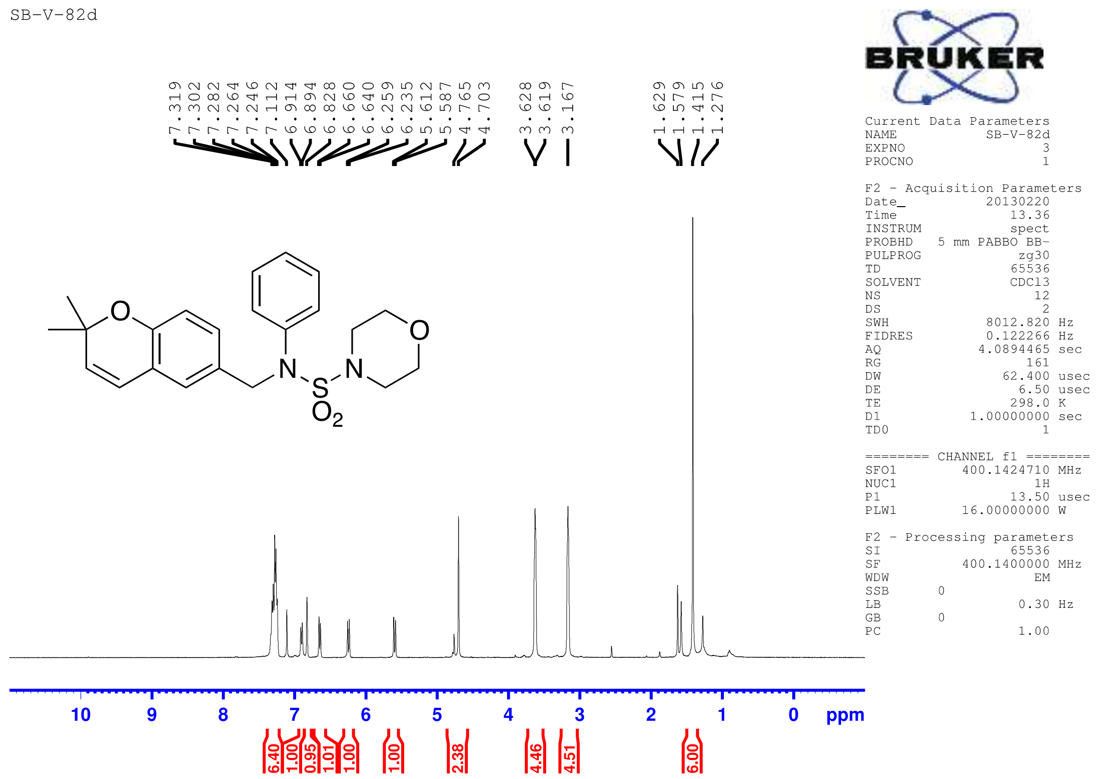

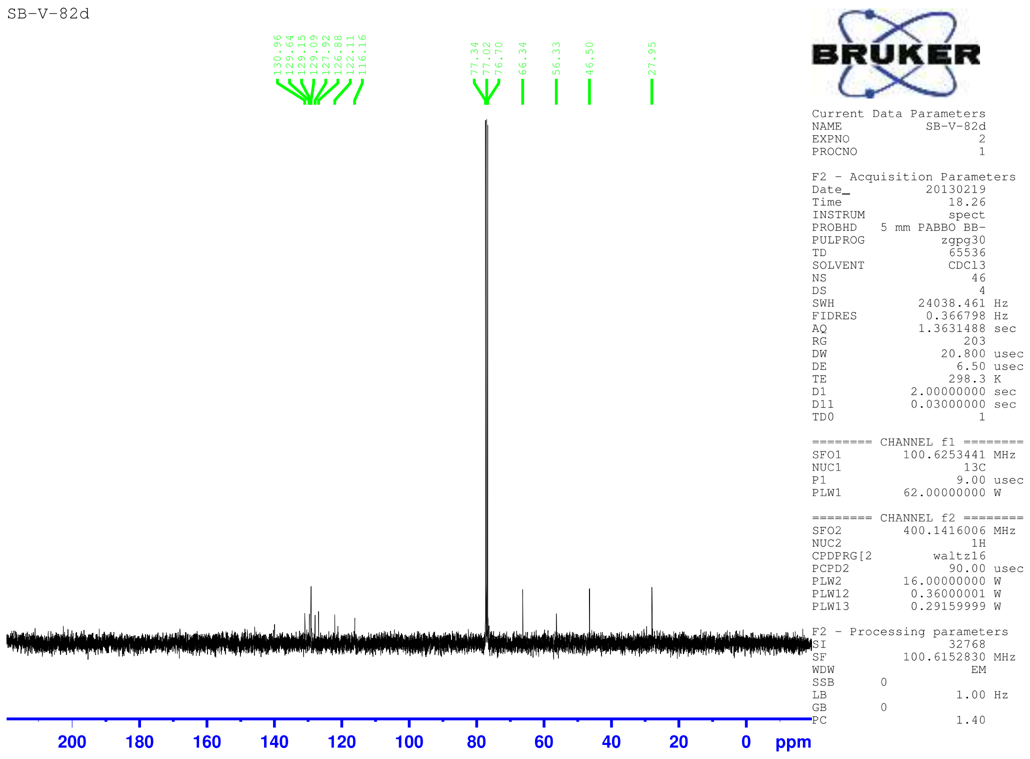

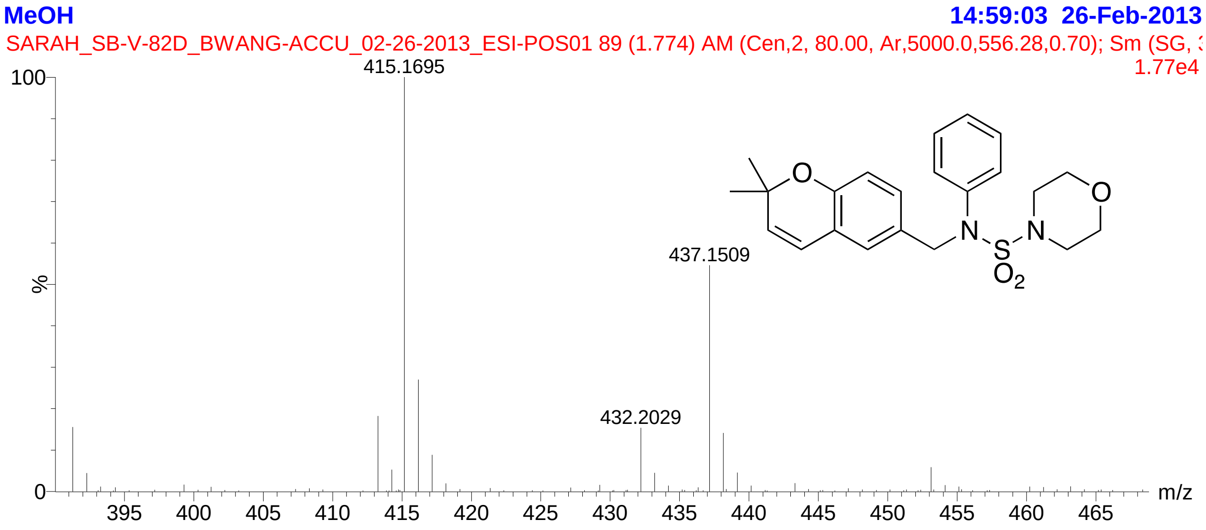

Elemental Composition Report

Single Mass Analysis
Tolerance = 5.0 PPM / DBE: min = -1.5, max = 50.0
Element prediction: Off
Number of isotope peaks used for i-FIT = 3

Monoisotopic Mass, Odd and Even Electron Ions
1658 formula(e) evaluated with 7 results within limits (up to 100 closest results for each mass)
Elements Used:
C: 1-150 H: 1-150 N: 1-6 O: 1-30 S: 1-10

Minimum:				-1.5			
Maximum:		5.0	5.0	50.0			
Mass	Calc. Mass	mDa	PPM	DBE	i-FIT		Formula
415.1695	415.1692	0.3	0.7	10.5	1.3		C22 H27 N2 O4 S

_N_-Cyclobutyl-_N_-((2,2-dimethyl-2_H_-chromen-6-yl)methyl)morpholine-4-sulfonamide (6b)

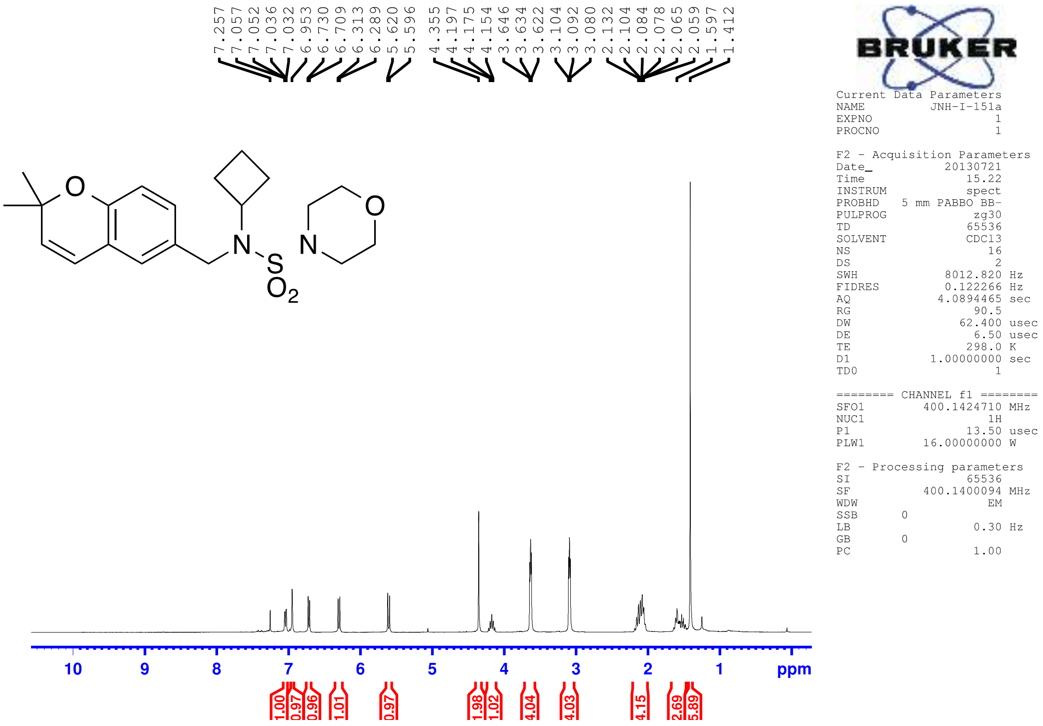

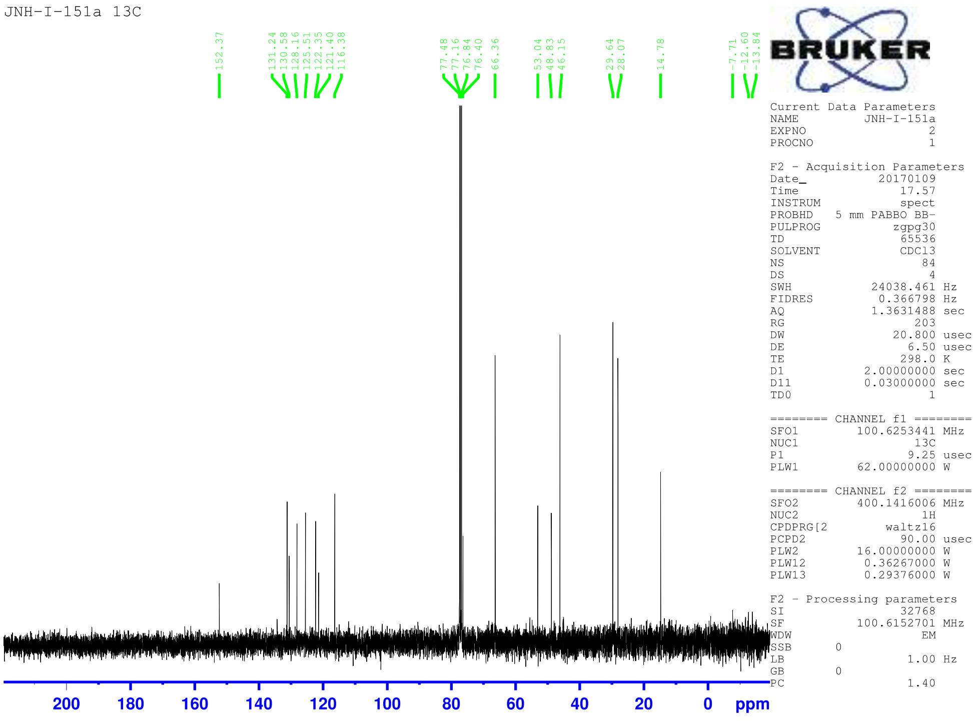

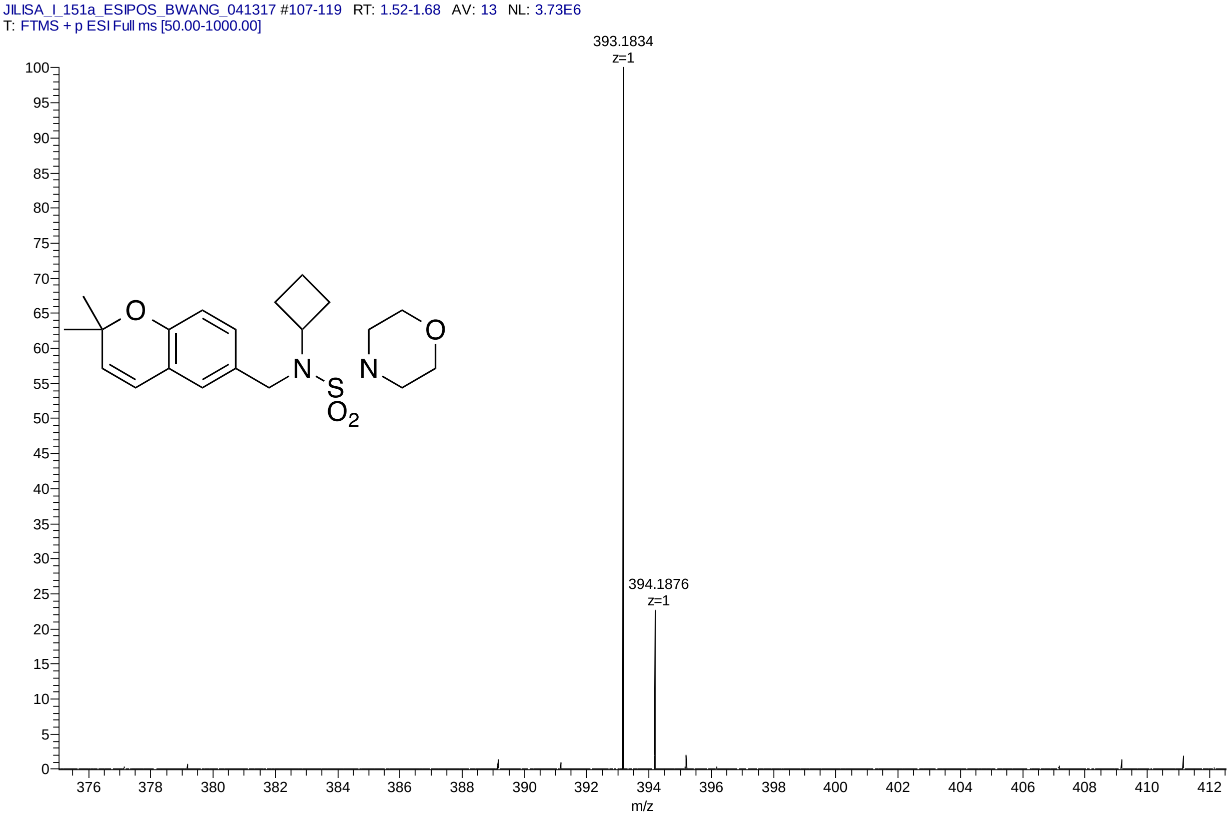
JILISA_I_151a_ESIPOS_BWANG_041317 #107-119 RT: 1.52-1.68 AV: 13 NL: 3.73E6
T: FTMS + p ESI Full ms [50.00-1000.00]
393.1834
z=1
394.1876
z=1
m/z
O
N
S
O2
N
O

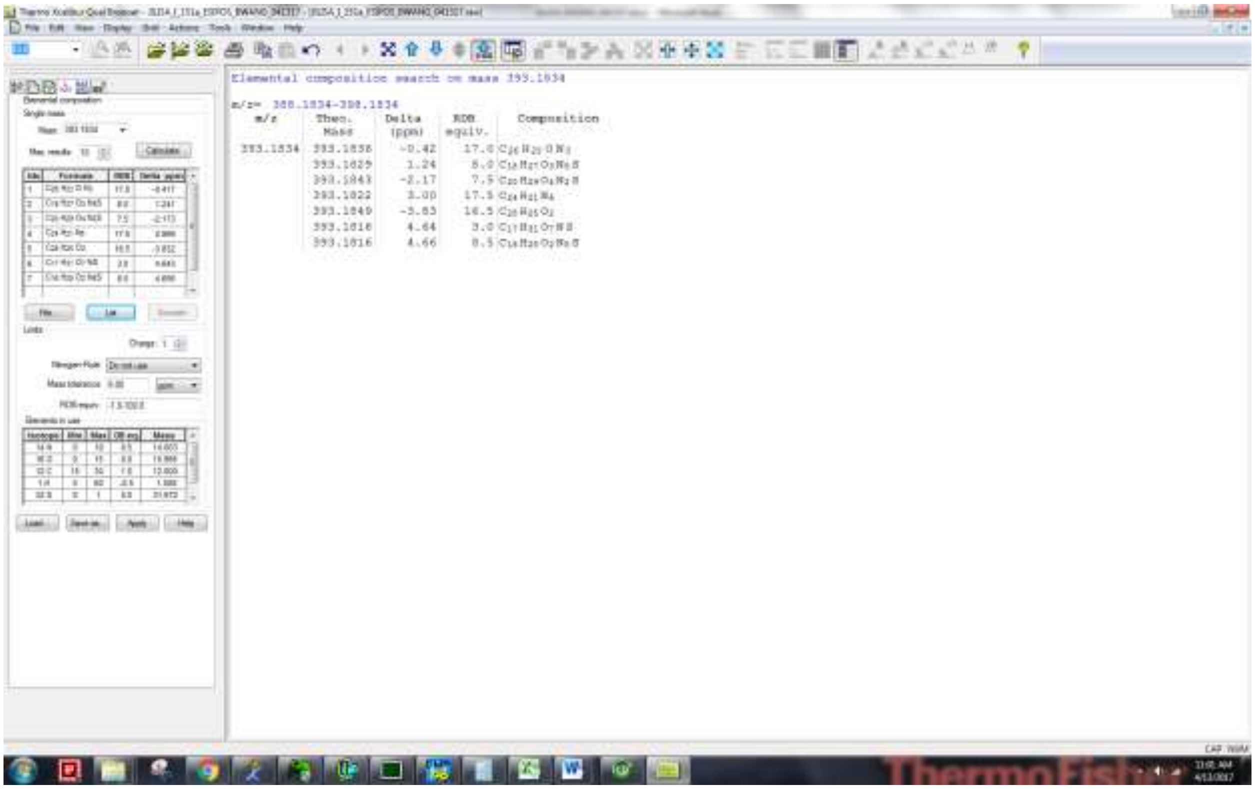
Elemental composition search on mass 393.1834
m/z
Theo. Mass
Delta (ppm)
RDB equiv.
Composition
393.1834
393.1836
-0.42
17.0
393.1829
1.24
8.0
393.1843
-2.17
7.5
393.1822
3.00
17.5
393.1849
-3.83
16.5
393.1816
4.64
3.0
393.1816
4.66
8.5

4-(4-Bromobenzyl)morpholine (8a)

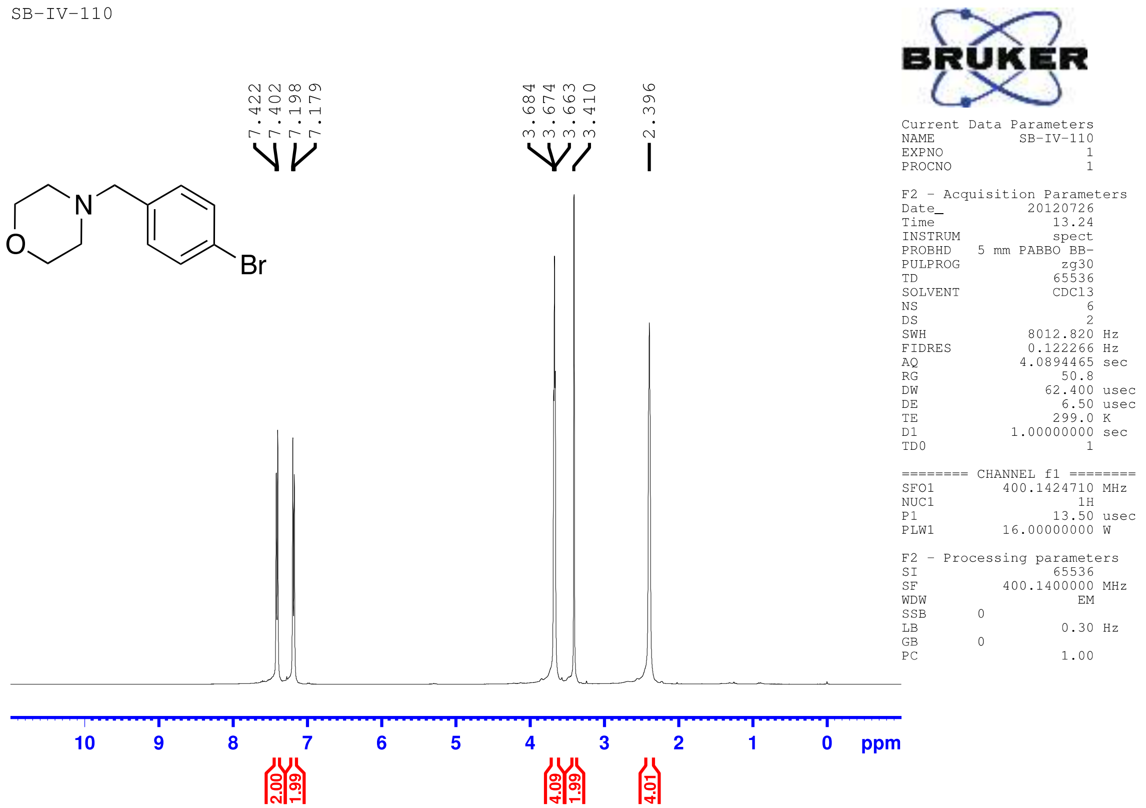

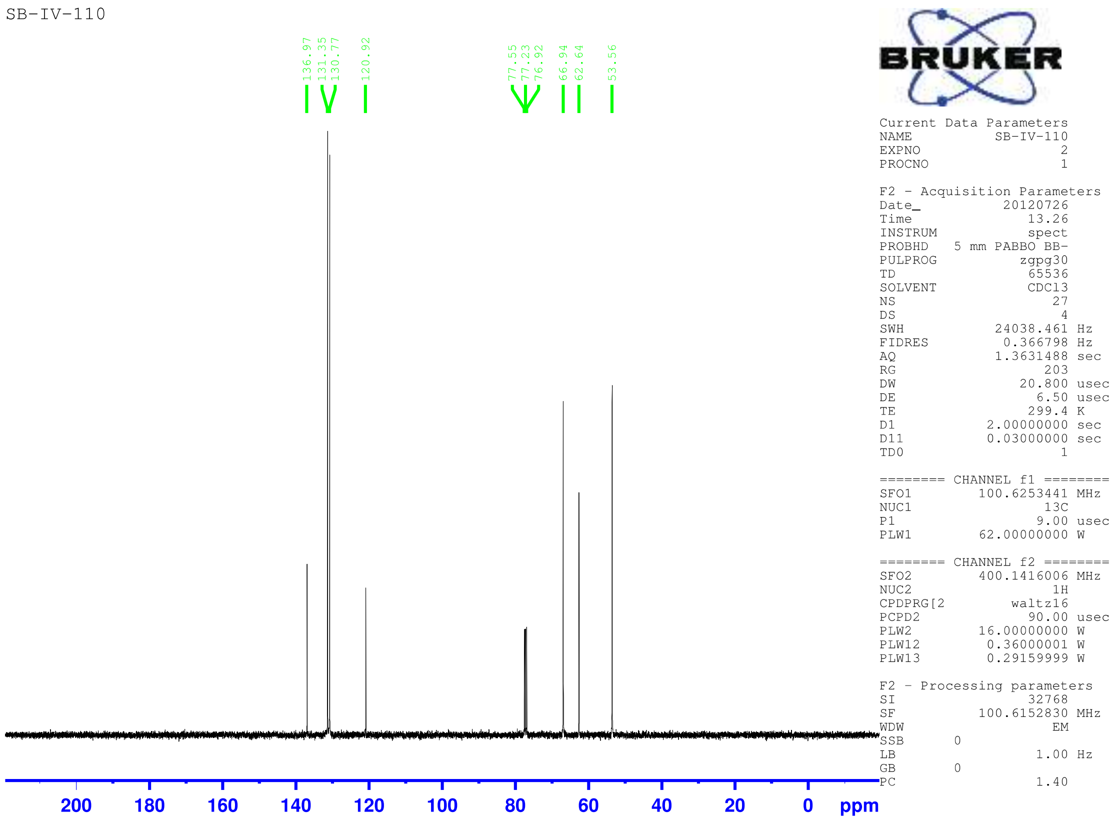

100%MeOH+0.1%HCOOH 13:20:16 21-Feb-2012

SARAH_SB-III-91B_BWANG-ACCU_02-21-2012_POS 100 (1.861) AM (Cen,4, 80.00, Ar,5000.0,556.28,0.70); Cm (98:1

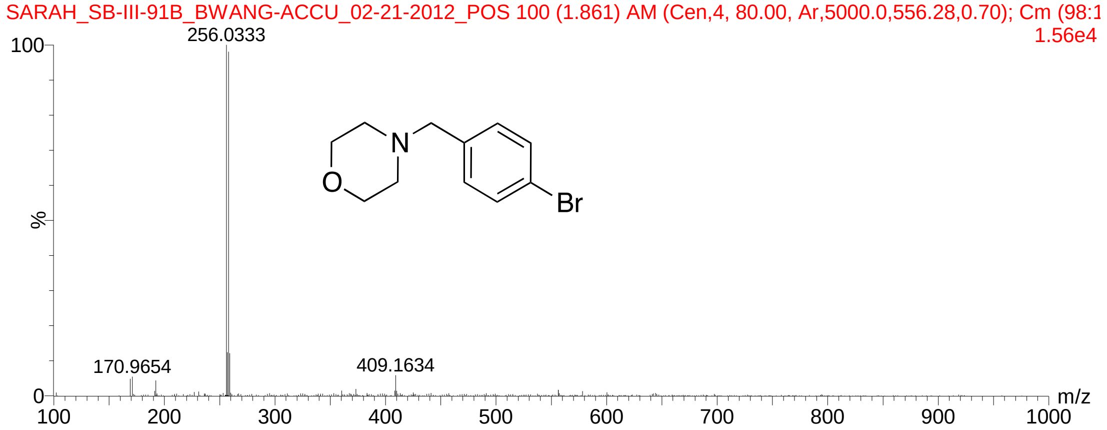

100%MeOH+0.1%HCOOH 13:20:16 21-Feb-2012

SARAH_SB-III-91B_BWANG-ACCU_02-21-2012_POS 100 (1.861) AM (Cen,4, 80.00, Ar,5000.0,556.28,0.70); Cm (98:1

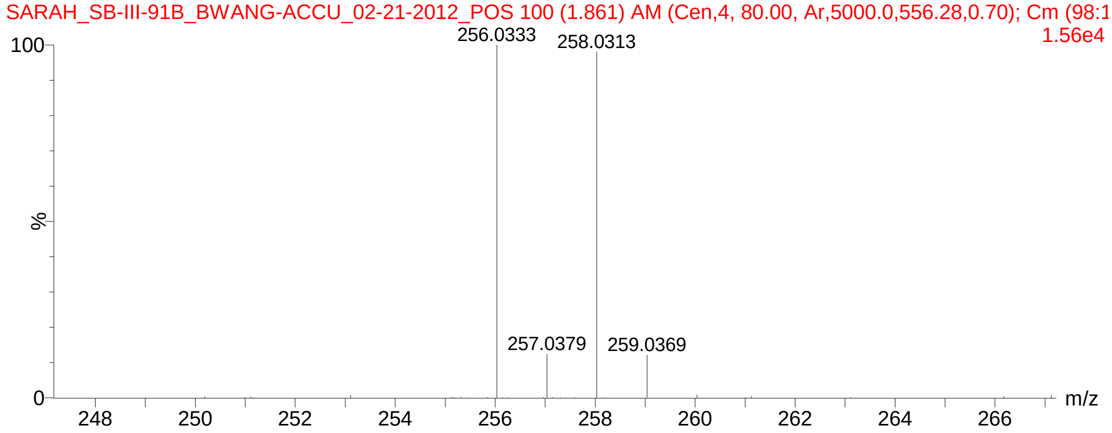

Elemental Composition Report

Single Mass Analysis

Tolerance = 5.0 PPM / DBE: min = -1.5, max = 50.0

Element prediction: Off

Number of isotope peaks used for i-FIT = 3

Monoisotopic Mass, Even Electron Ions

106 formula(e) evaluated with 1 results within limits (all results (up to 1000) for each mass)

Elements Used:

C: 1-100 H: 1-100 N: 1-15 O: 1-20 Br: 1-5

Minimum:				-1.5			
Maximum:		5.0	5.0	50.0			
Mass	Calc. Mass	mDa		PPM	DBE	i-FIT	Formula
256.0333	256.0337	-0.4	-1.6	4.5	1.6		C11 H15 N O Br

3-(2-Bromobenzyl)morpholine (8b)

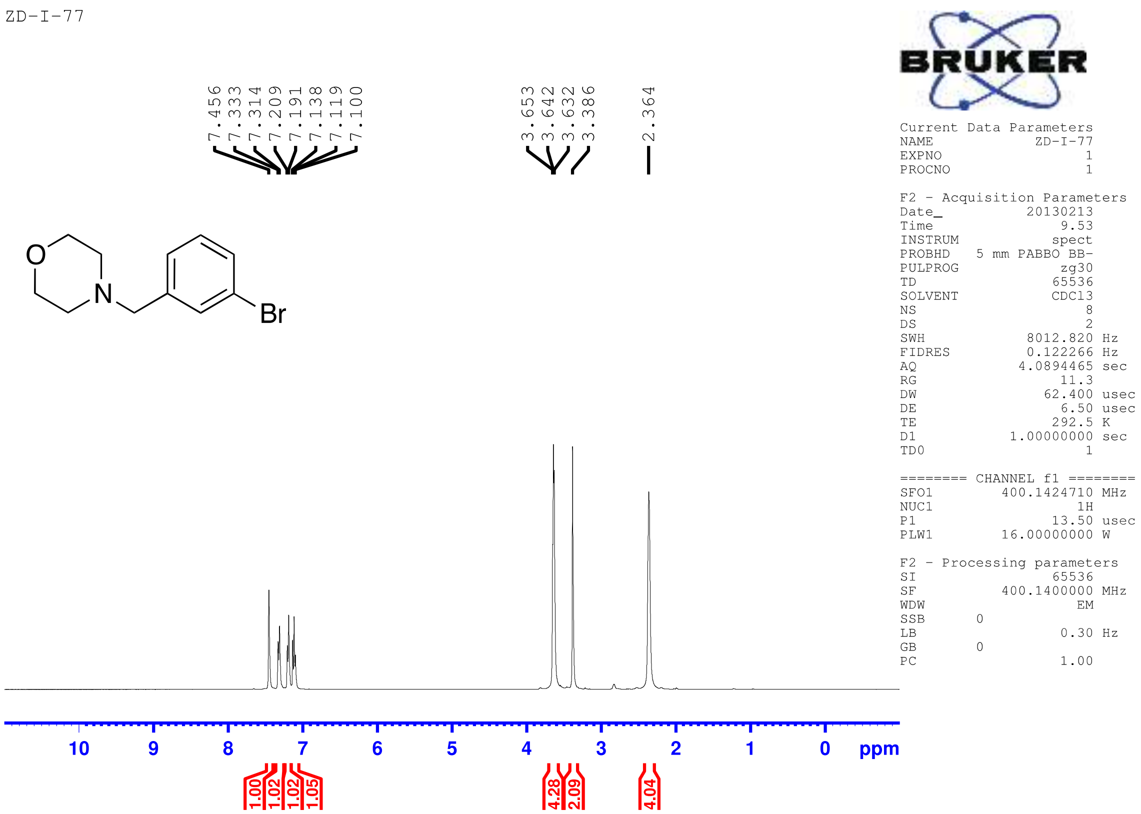

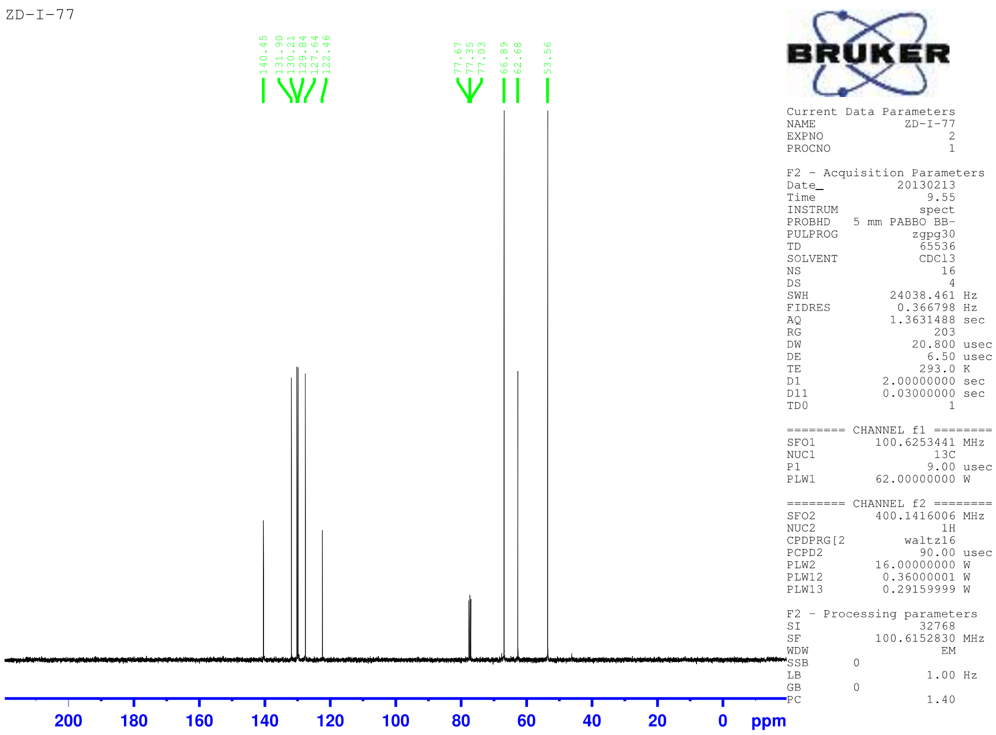

90%ACN+0.1%HCOOH 16:27:04 31-Oct-2012
SARAH_SB-IV-113_BWANG-ACCU_10-31-2012_ESI-POS001 81 (0.849) AM (Cen,2, 80.00, Ar,5000.0,556.28,0.70); Cm (81:10

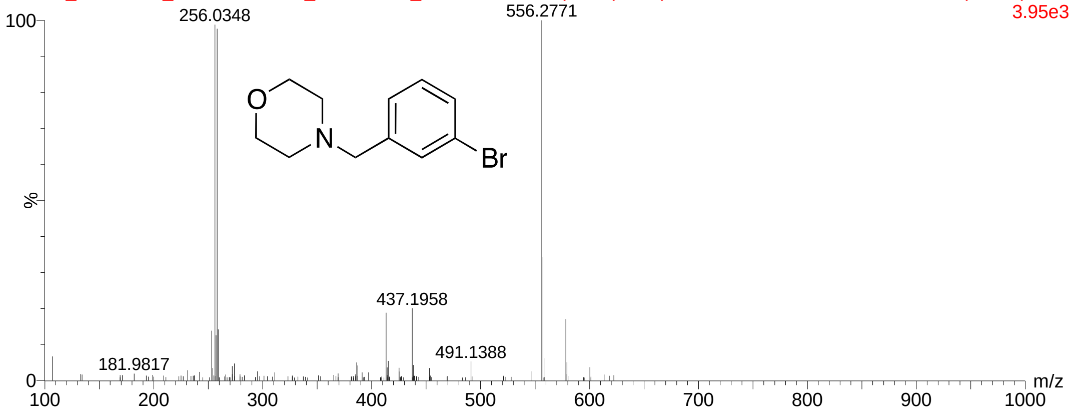

90%ACN+0.1%HCOOH 16:27:04 31-Oct-2012
SARAH_SB-IV-113_BWANG-ACCU_10-31-2012_ESI-POS001 81 (0.849) AM (Cen,2, 80.00, Ar,5000.0,556.28,0.70); Cm (81:10

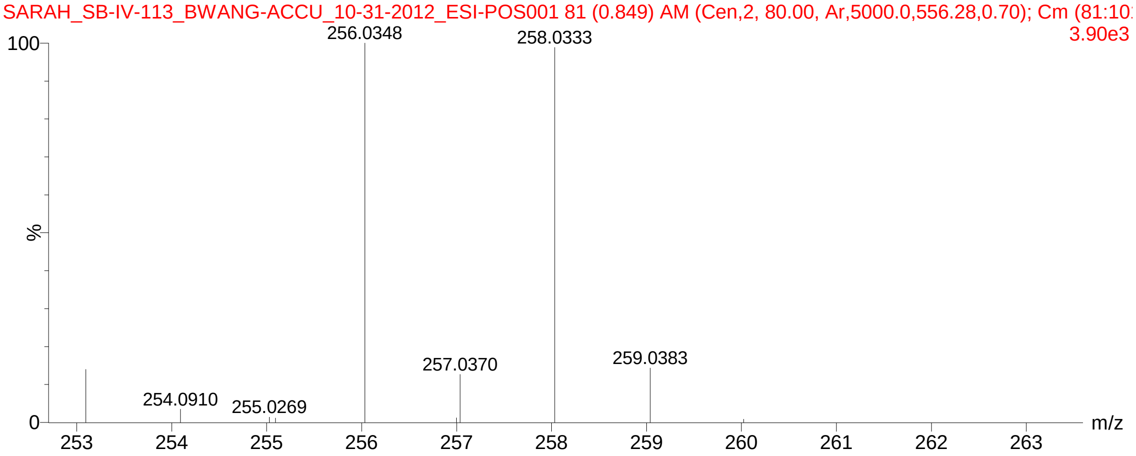

Elemental Composition Report

Single Mass Analysis
Tolerance = 5.0 PPM / DBE: min = -1.5, max = 50.0
Element prediction: Off
Number of isotope peaks used for i-FIT = 3

Monoisotopic Mass, Odd and Even Electron Ions
106 formula(e) evaluated with 1 results within limits (all results (up to 1000) for each mass)
Elements Used:
C: 1-200 H: 1-200 N: 1-20 O: 1-30 Br: 1-5

Minimum:				-1.5			
Maximum:		5.0	5.0	50.0			
Mass	Calc. Mass	mDa	PPM	DBE	i-FIT		Formula
256.0348	256.0337	1.1	4.3	4.5	0.7		C11 H15 N O Br

4-(2-Bromobenzyl)morpholine (8c)

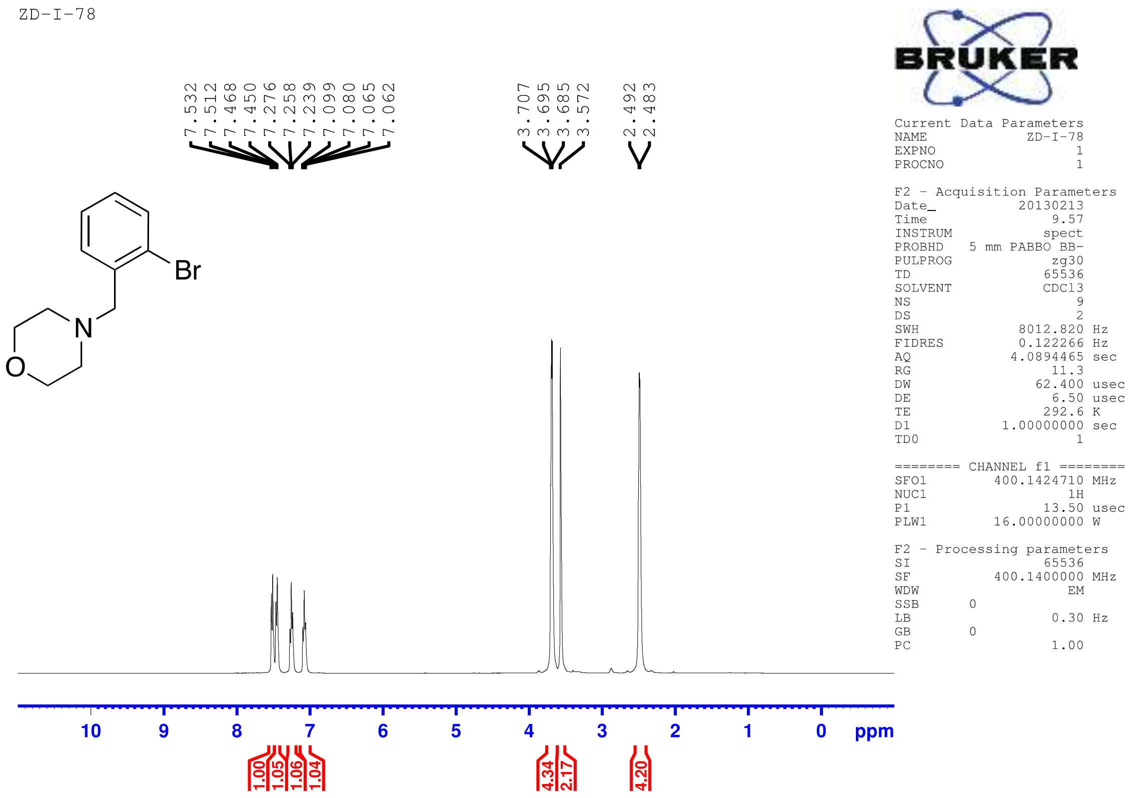

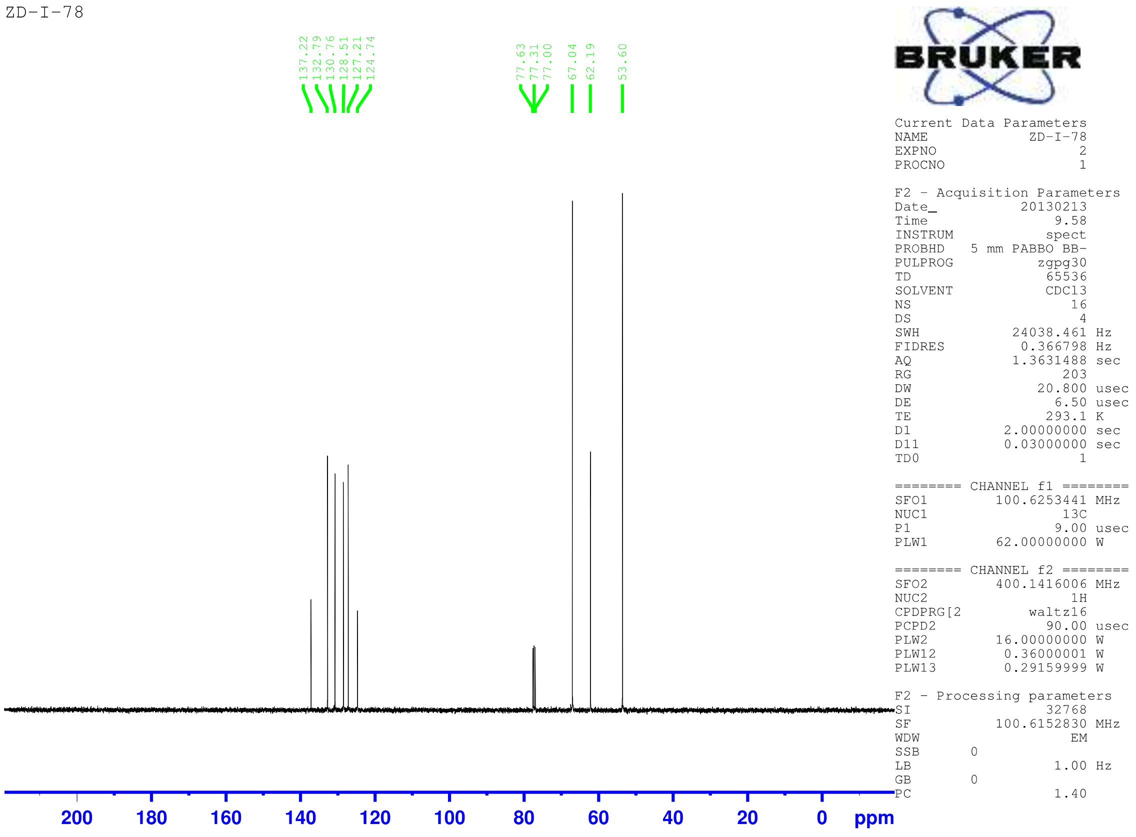

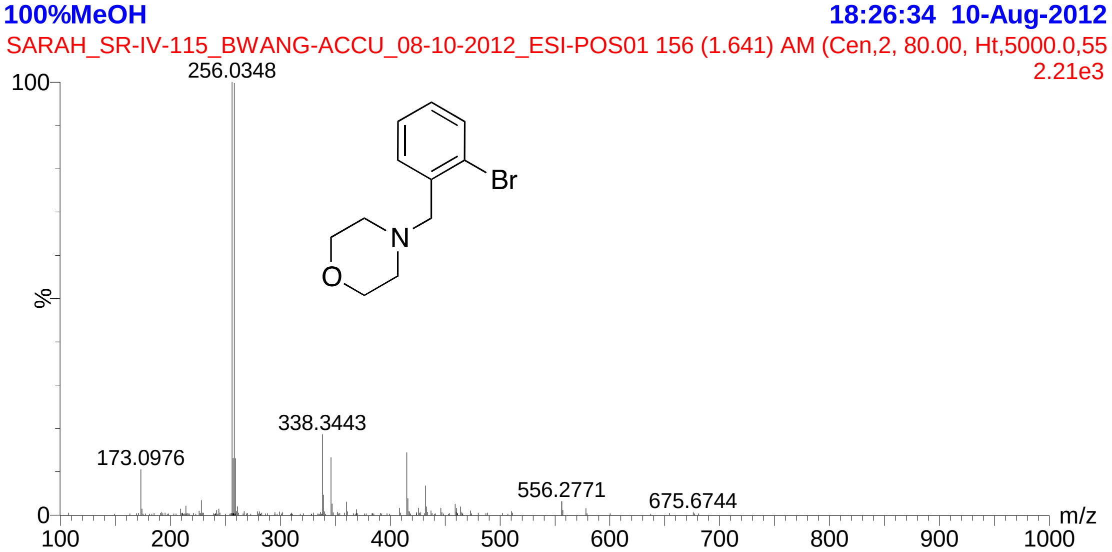

Elemental Composition Report

Single Mass Analysis
Tolerance = 5.0 PPM / DBE: min = -1.5, max = 50.0
Element prediction: Off
Number of isotope peaks used for i-FIT = 3

Monoisotopic Mass, Even Electron Ions
106 formula(e) evaluated with 1 results within limits (all results (up to 1000) for each mass)
Elements Used:
C: 1-200 H: 1-200 N: 1-15 O: 1-100 Br: 1-5

Minimum:				-1.5			
Maximum:		5.0	5.0	50.0			
Mass	Calc. Mass	mDa		PPM	DBE	i-FIT	Formula
256.0348	256.0337	1.1	4.3	4.5	0.5		C11 H15 N O Br

4-(Morpholinomethyl)benzaldehyde (9a)

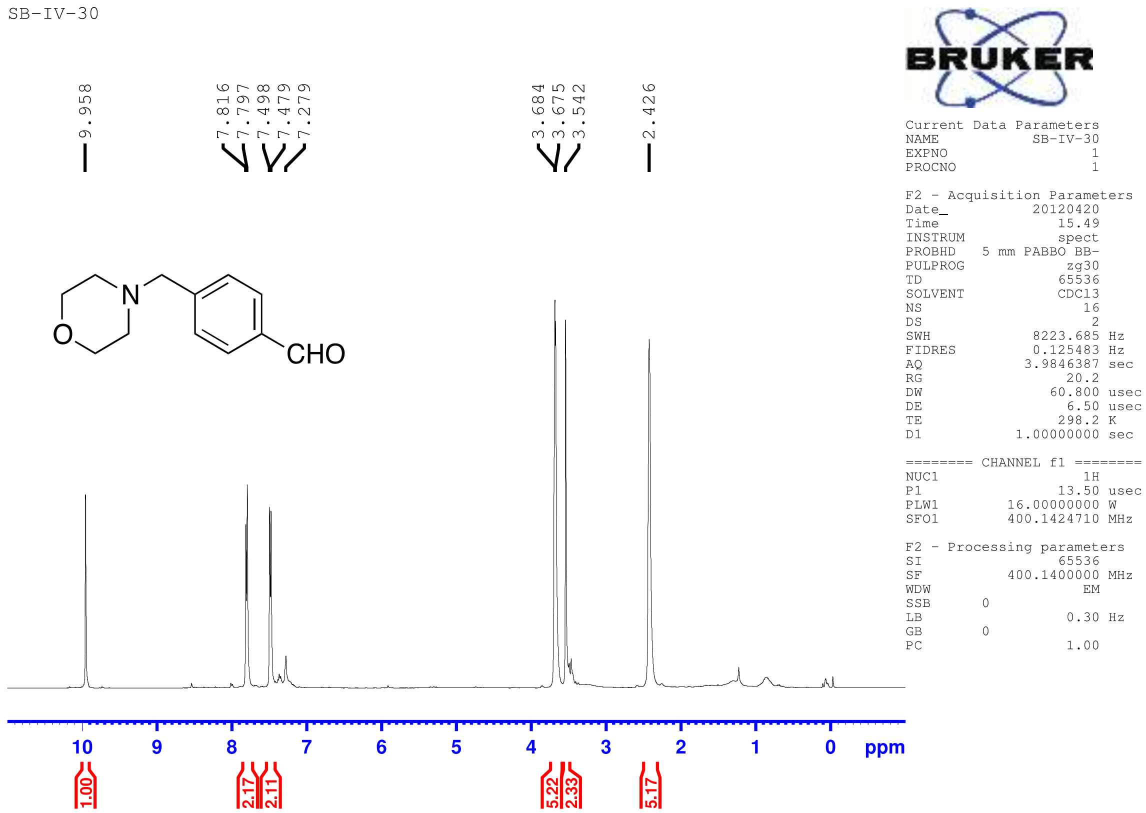

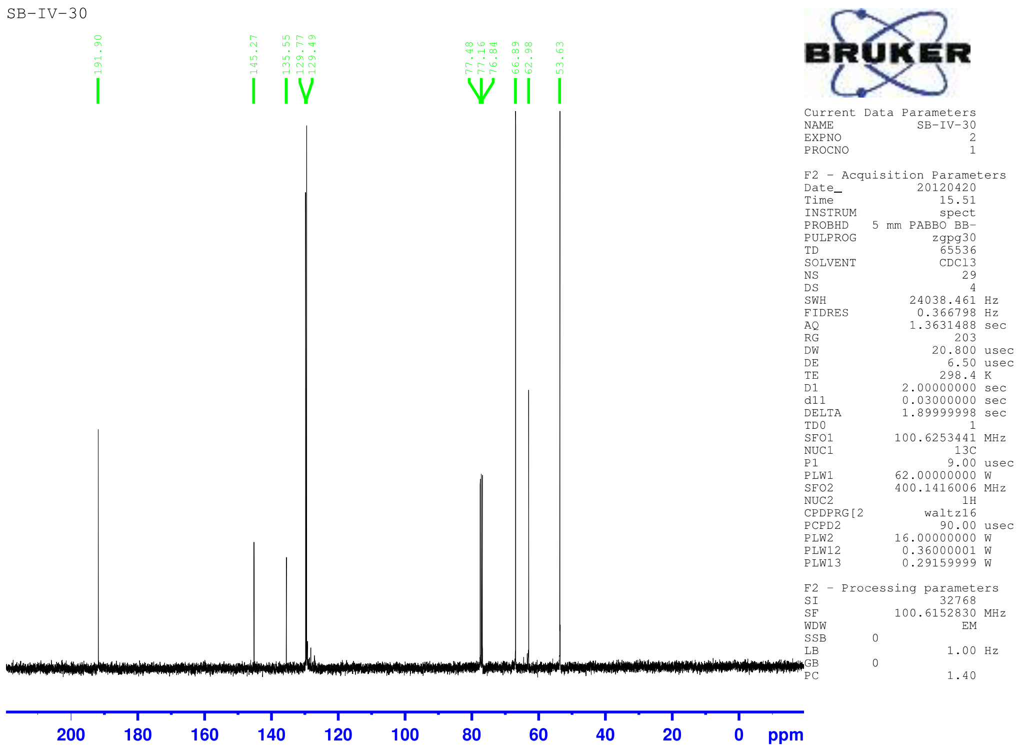

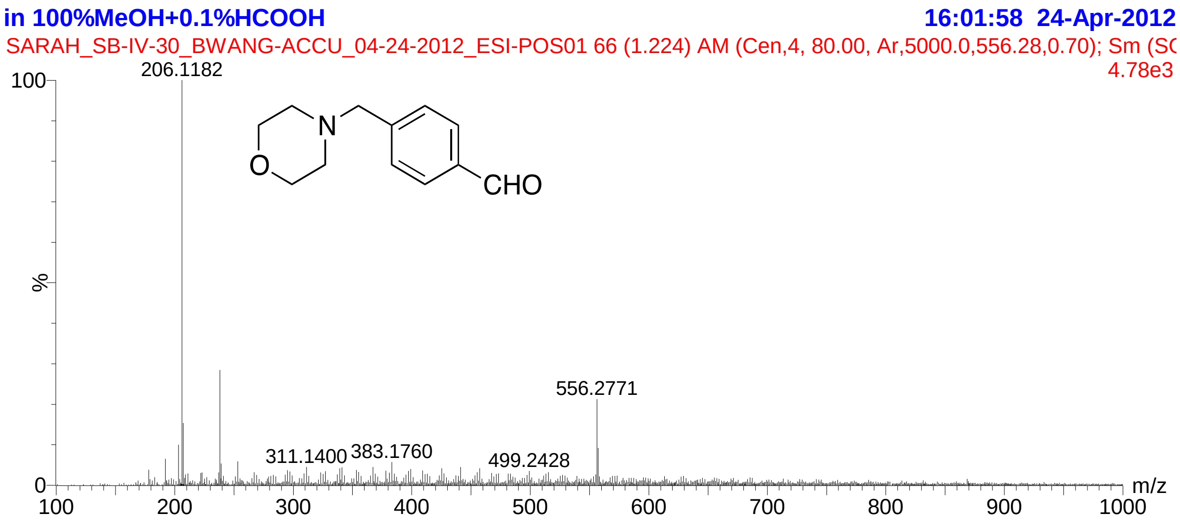

Elemental Composition Report

Single Mass Analysis

Tolerance = 5.0 PPM / DBE: min = -1.5, max = 50.0

Element prediction: Off

Number of isotope peaks used for i-FIT = 3

Monoisotopic Mass, Even Electron Ions

181 formula(e) evaluated with 1 results within limits (all results (up to 1000) for each mass)

Elements Used:

C: 1-100 H: 1-100 N: 1-15 O: 1-35 80Se: 0-1

Minimum:				-1.5			
Maximum:		5.0	5.0	50.0			
Mass	Calc. Mass	mDa		PPM	DBE	i-FIT	Formula
206.1182	206.1181	0.1	0.5	5.5	5.0		C12 H16 N O2

3-(Morpholinomethyl)benzaldehyde (9b)

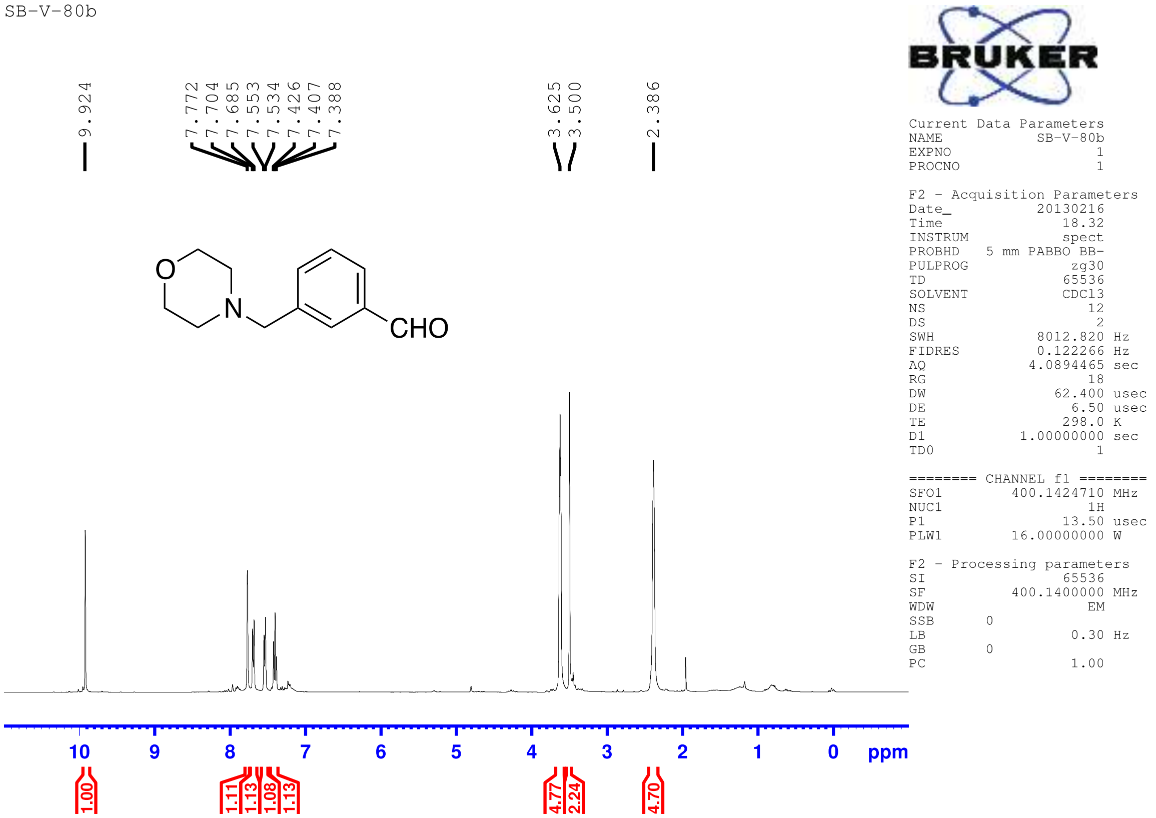

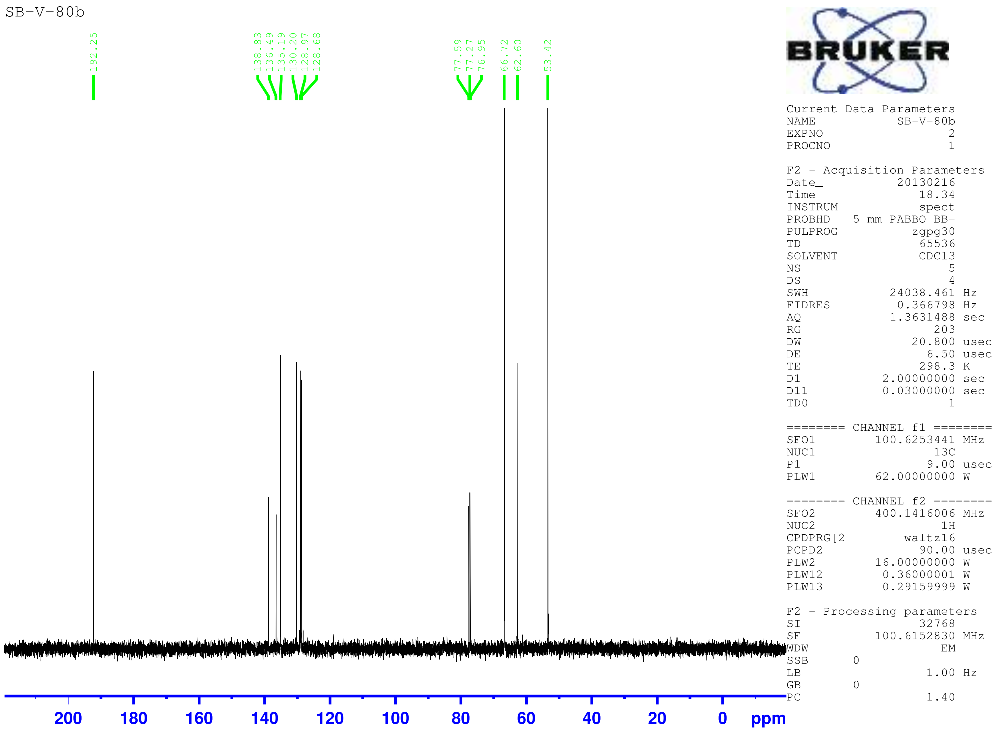

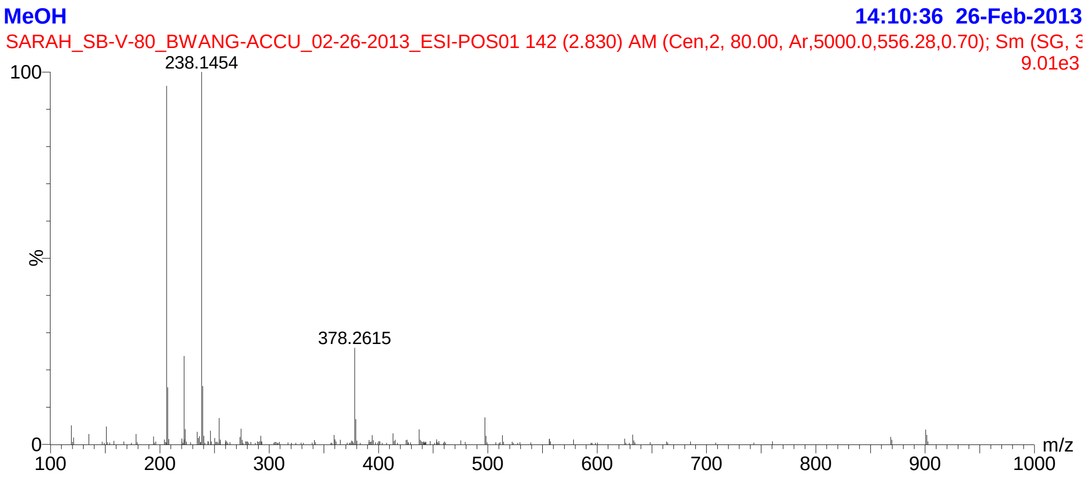

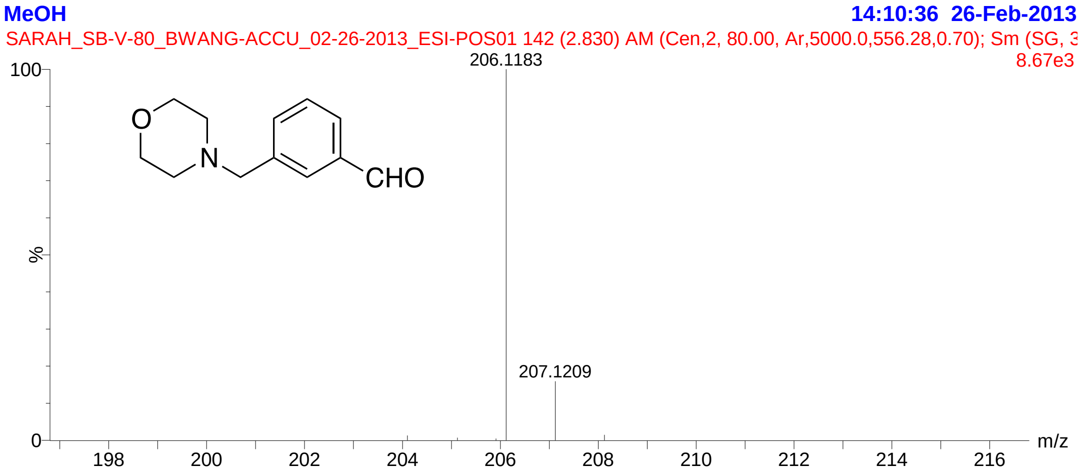

Elemental Composition Report

Single Mass Analysis
Tolerance = 5.0 PPM / DBE: min = -1.5, max = 50.0
Element prediction: Off
Number of isotope peaks used for i-FIT = 3

Monoisotopic Mass, Even Electron Ions
105 formula(e) evaluated with 1 results within limits (up to 100 closest results for each mass)
Elements Used:
C: 1-150 H: 1-150 N: 1-6 O: 1-30

Minimum:				-1.5		
Maximum:		5.0	5.0	50.0		
Mass	Calc. Mass	mDa	PPM	DBE	i-FIT	Formula
206.1183	206.1181	0.2	1.0	5.5	8.5	C12 H16 N O2

2-(Morpholinomethyl)benzaldehyde (9c)

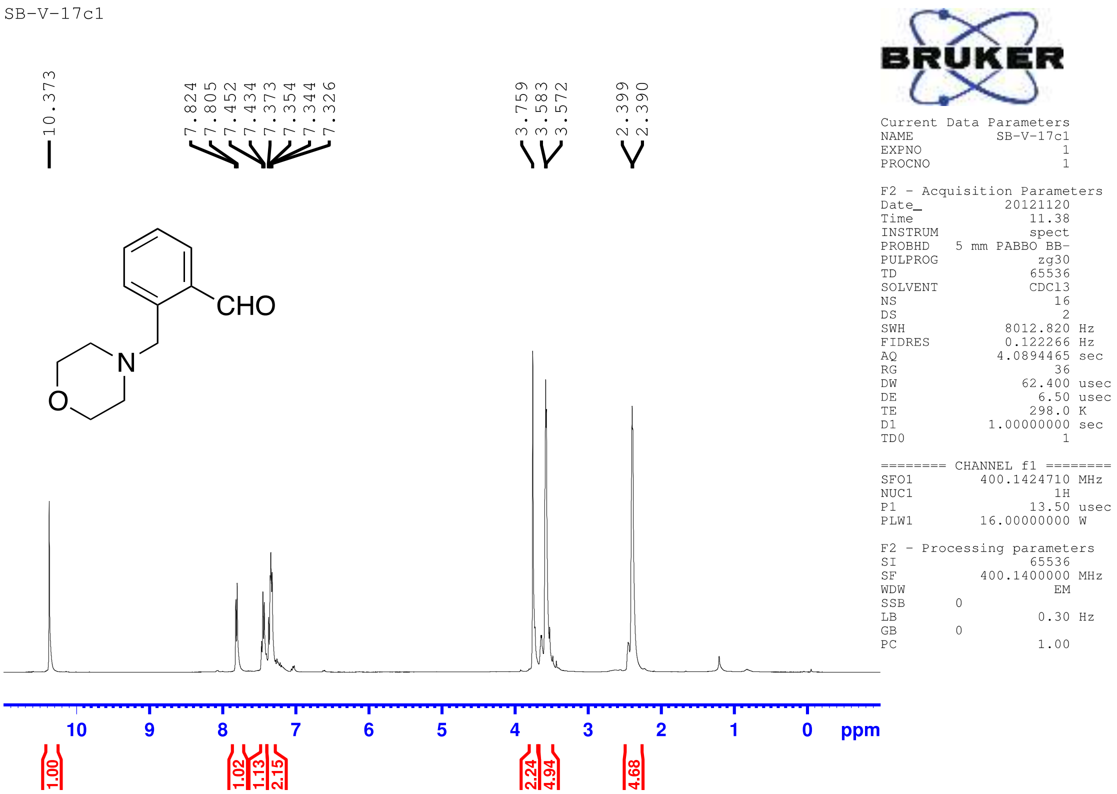

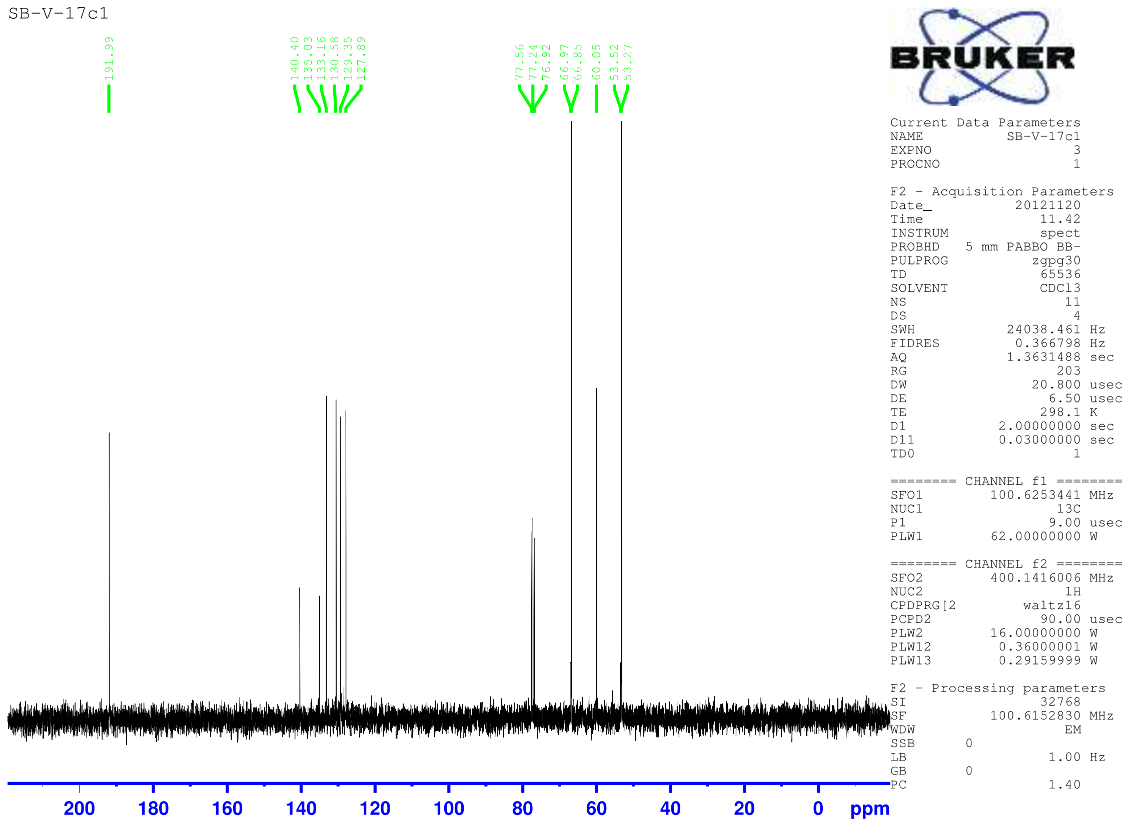

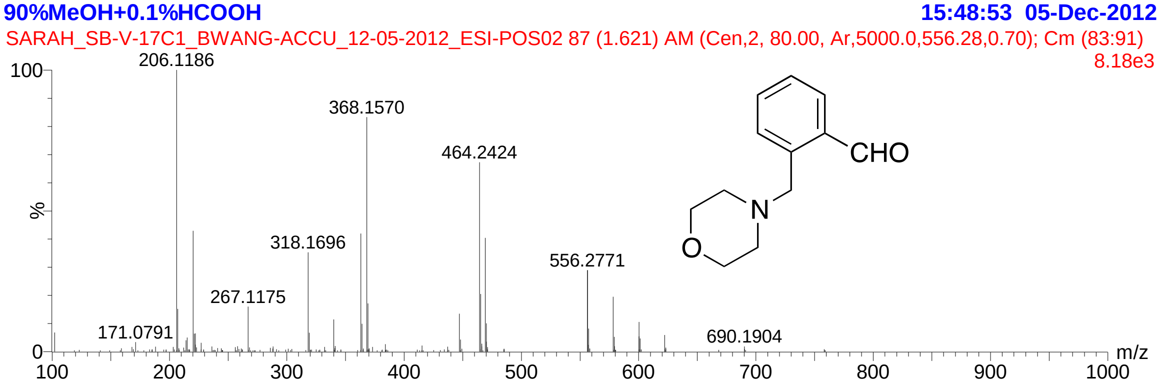

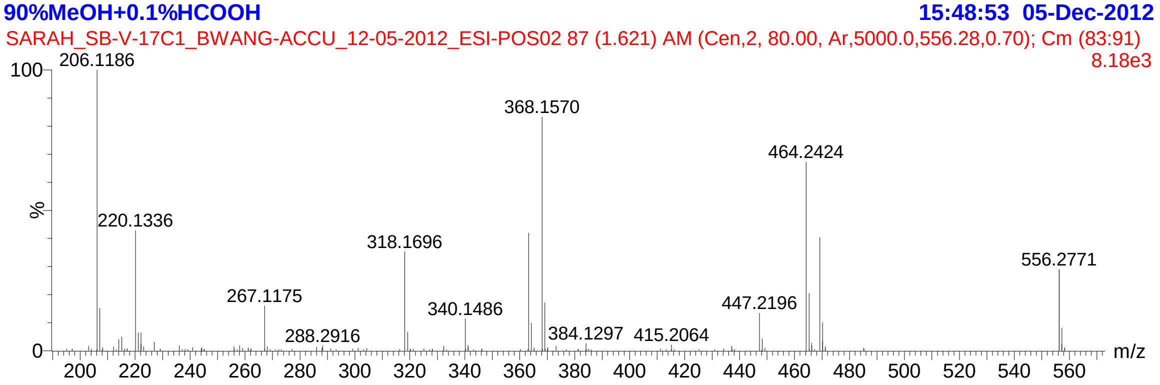

Elemental Composition Report

Single Mass Analysis
Tolerance = 5.0 PPM / DBE: min = -1.5, max = 50.0
Element prediction: Off
Number of isotope peaks used for i-FIT = 3

Monoisotopic Mass, Odd and Even Electron Ions
145 formula(e) evaluated with 1 results within limits (all results (up to 1000) for each mass)
Elements Used:
C: 1-200 H: 1-200 N: 1-20 O: 1-30

Minimum:					-1.5		
Maximum:		5.0	5.0		50.0		
Mass	Calc. Mass	mDa	PPM	DBE	i-FIT		Formula
206.1186	206.1181	0.5	2.4	5.5	5.5		C12 H16 N O2

N-(4-(morpholinomethyl)benzyl)aniline (10a)

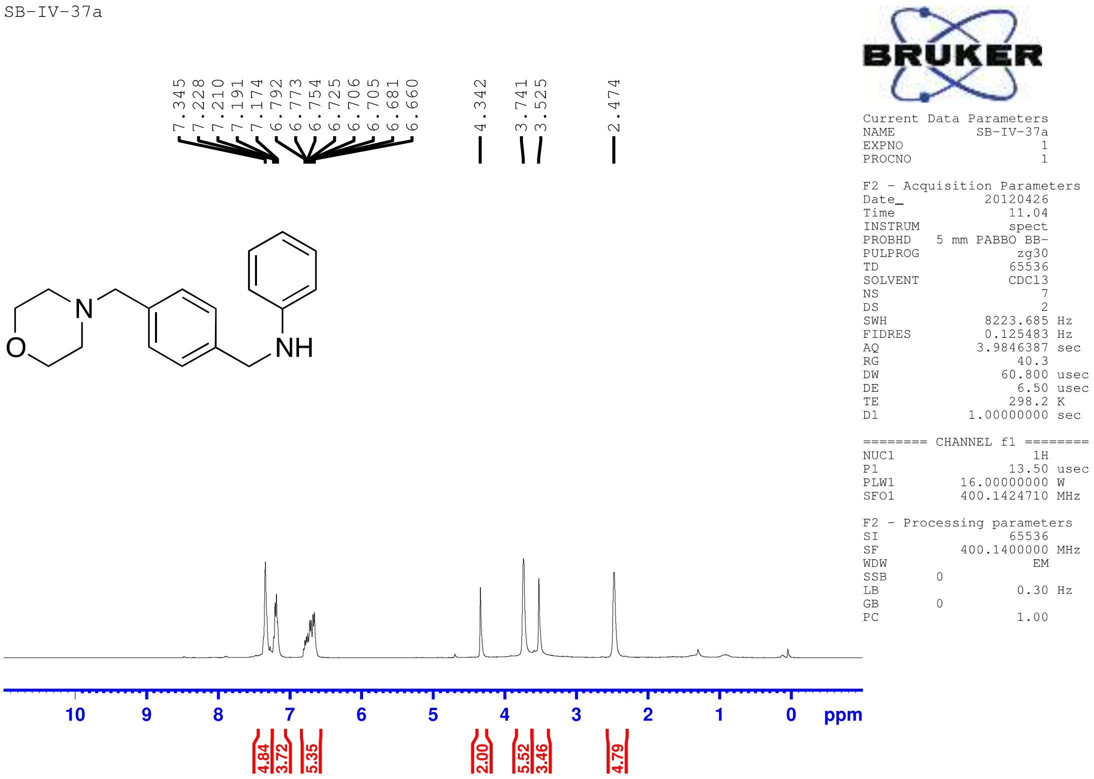

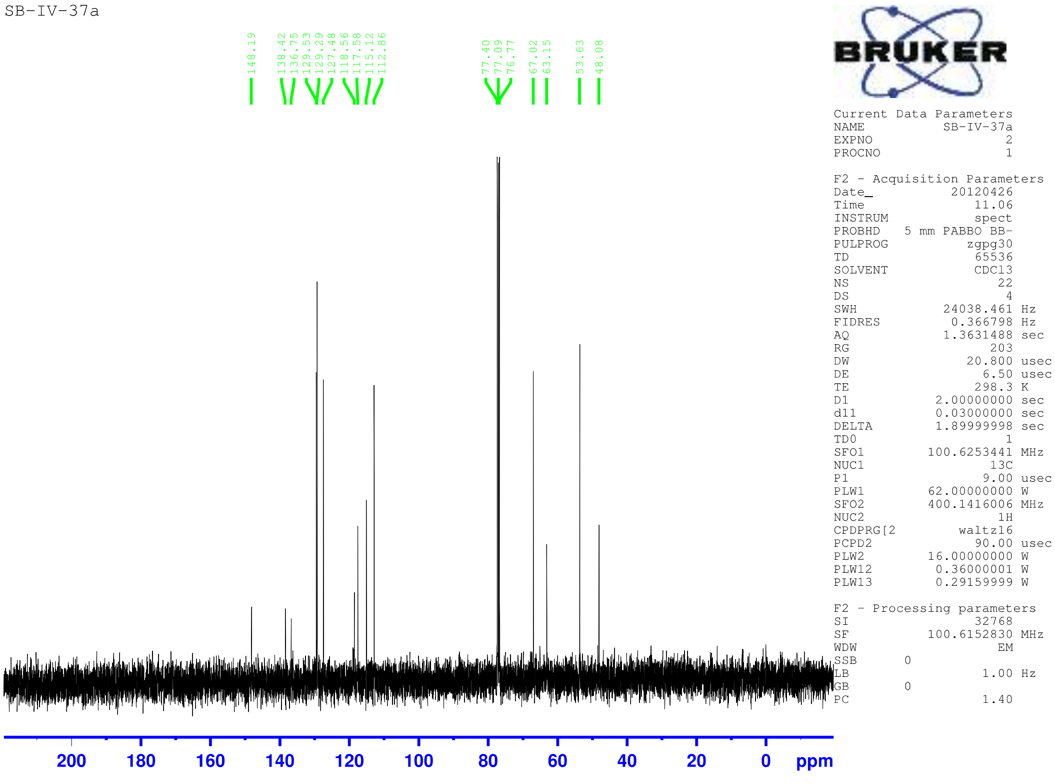

in 90%MeOH 17:58:03 05-Mar-2013
SARAH_SB-V-93B_BWANG-ACCU_03-05-2013_ESI-POS01 100 (1.988) AM (Cen,2, 80.00, Ar,5000.0,556.28,0.70); Sm (SG, 1.70e4

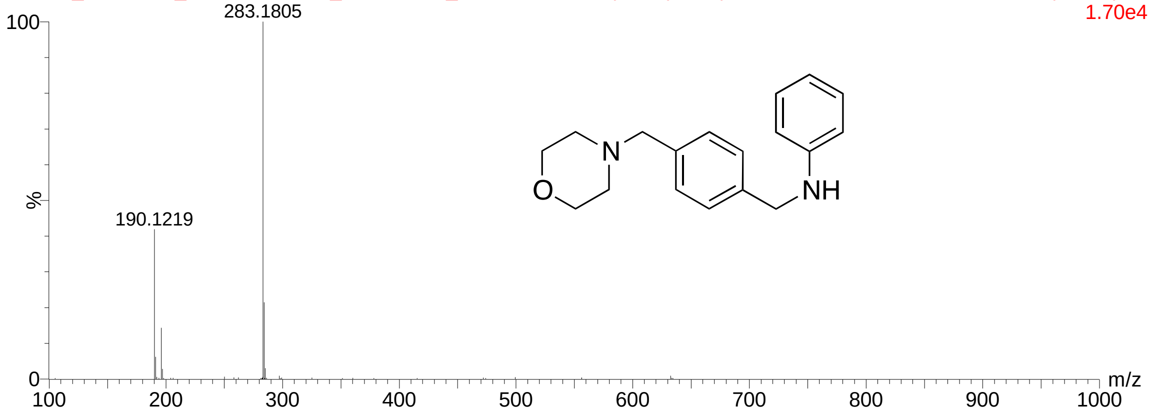

Elemental Composition Report

Single Mass Analysis
Tolerance = 5.0 PPM / DBE: min = -1.5, max = 50.0
Element prediction: Off
Number of isotope peaks used for i-FIT = 3

Monoisotopic Mass, Even Electron Ions
363 formula(e) evaluated with 2 results within limits (up to 100 closest results for each mass)
Elements Used:
C: 1-150 H: 1-150 N: 1-30 O: 1-60

Minimum:				-1.5		
Maximum:		5.0	5.0	50.0		
Mass	Calc. Mass	mDa	PPM	DBE	i-FIT	Formula
283.1805	283.1810	-0.5	-1.8	8.5	12.4	C18 H23 N2 O
	283.1815	-1.0	-3.5	1.5	741.3	C3 H19 N14 O2

N-(3-(Morpholinomethyl)benzyl)aniline (10b)

SB-V-107d

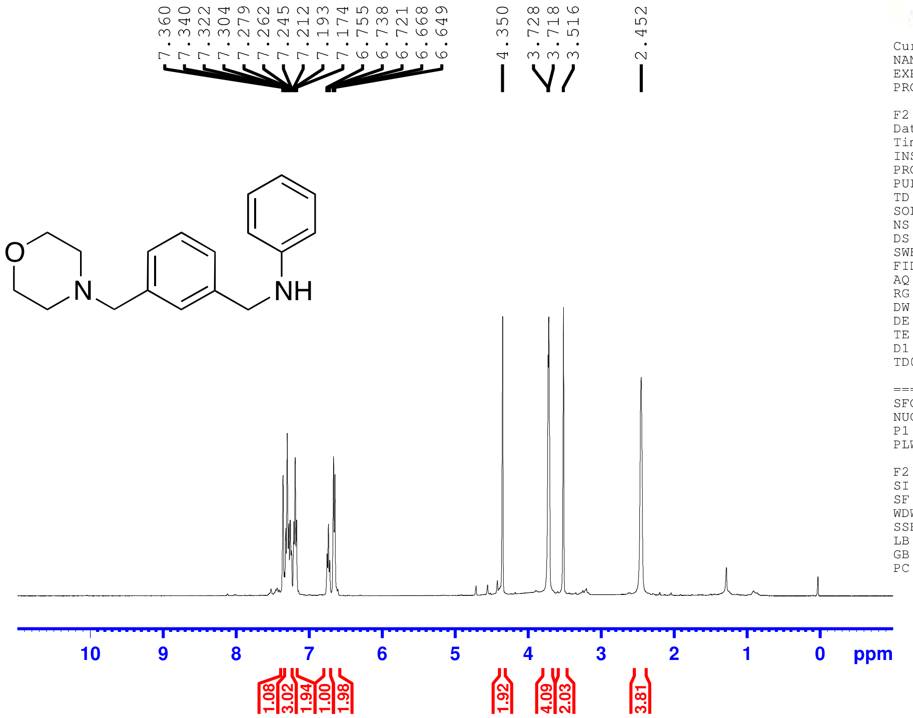


```
Current Data Parameters
NAME             SB-V-107d
EXPNO                    3
PROCNO                   1

F2 - Acquisition Parameters
Date_             20130319
Time                 13.06
INSTRUM              spect
PROBHD   5 mm PABBO BB-
PULPROG               zg30
TD                   65536
SOLVENT              CDCl3
NS                       6
DS                       2
SWH               8012.820 Hz
FIDRES            0.122266 Hz
AQ               4.0894465 sec
RG                     101
DW                  62.400 usec
DE                    6.50 usec
TE                   294.2 K
D1              1.00000000 sec
TD0                      1

======== CHANNEL f1 ========
SFO1           400.1424710 MHz
NUC1                    1H
P1                   13.50 usec
PLW1           16.00000000 W

F2 - Processing parameters
SI                   65536
SF             400.1400000 MHz
WDW                     EM
SSB      0
LB                    0.30 Hz
GB       0
PC                    1.00
```

SB-V-107d

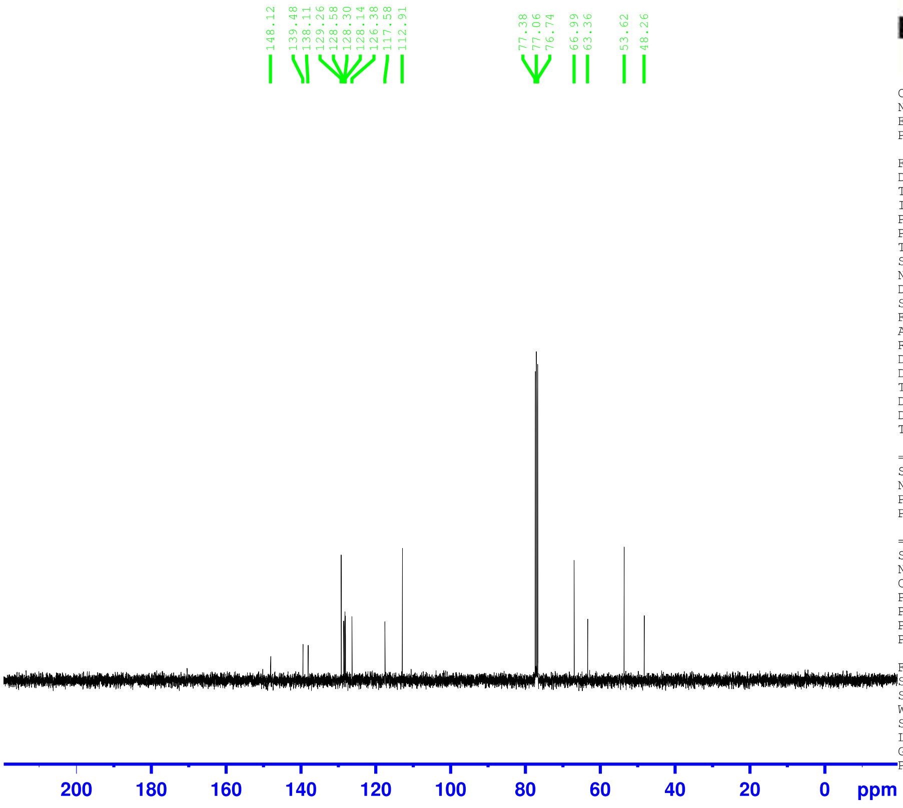


```
Current Data Parameters
NAME             SB-V-107d
EXPNO                    4
PROCNO                   1

F2 - Acquisition Parameters
Date_             20130319
Time                 13.08
INSTRUM              spect
PROBHD   5 mm PABBO BB-
PULPROG            zgpg30
TD                   65536
SOLVENT              CDCl3
NS                      30
DS                       4
SWH              24038.461 Hz
FIDRES            0.366798 Hz
AQ               1.3631488 sec
RG                     203
DW                  20.800 usec
DE                    6.50 usec
TE                   294.8 K
D1              2.00000000 sec
D11             0.03000000 sec
TD0                      1

======== CHANNEL f1 ========
SFO1           100.6253441 MHz
NUC1                   13C
P1                    9.00 usec
PLW1           62.00000000 W

======== CHANNEL f2 ========
SFO2           400.1416006 MHz
NUC2                    1H
CPDPRG[2           waltz16
PCPD2                90.00 usec
PLW2           16.00000000 W
PLW12           0.36000001 W
PLW13           0.29159999 W

F2 - Processing parameters
SI                   32768
SF             100.6152830 MHz
WDW                     EM
SSB      0
LB                    1.00 Hz
GB       0
PC                    1.40
```

80%MeOH 14:18:33 15-Mar-2013

SARAH_SB-V-107D_BWANG-ACCU_03152013_ESI-POS02 197 (3.921) AM (Top,2, Ar,5000.0,556.28,0.70); Cm (193:201) 6.70e3

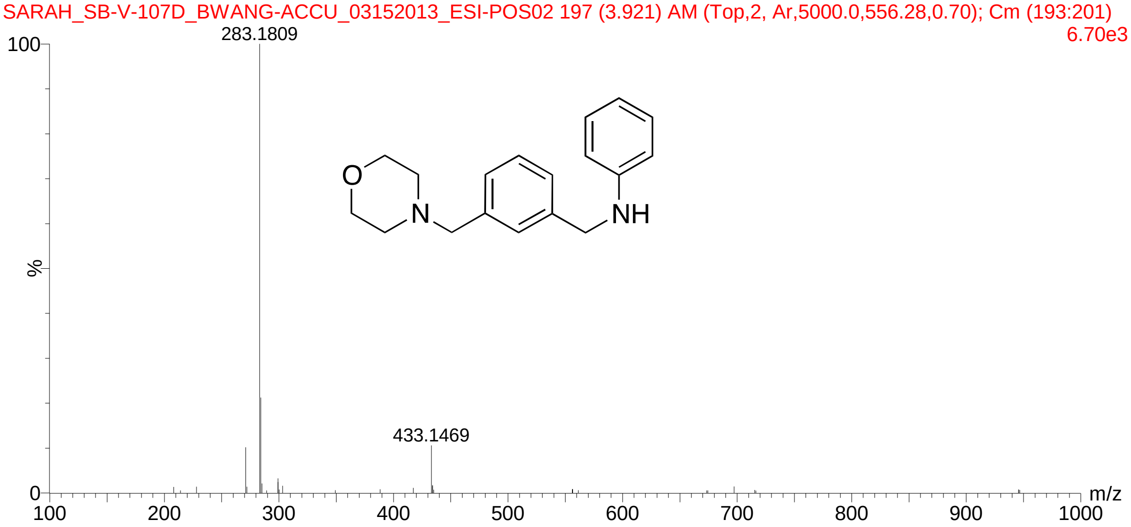

Elemental Composition Report

Single Mass Analysis
Tolerance = 5.0 PPM / DBE: min = -1.5, max = 50.0
Element prediction: Off
Number of isotope peaks used for i-FIT = 3

Monoisotopic Mass, Odd and Even Electron Ions
363 formula(e) evaluated with 3 results within limits (up to 100 closest results for each mass)
Elements Used:
C: 1-150 H: 1-150 N: 1-30 O: 1-60

Minimum:				-1.5		
Maximum:		5.0	5.0	50.0		
Mass	Calc. Mass	mDa	PPM	DBE	i-FIT	Formula
283.1809	283.1810	-0.1	-0.4	8.5	1.7	C18 H23 N2 O

N-(2-(Morpholinomethyl)benzyl)aniline (10c)

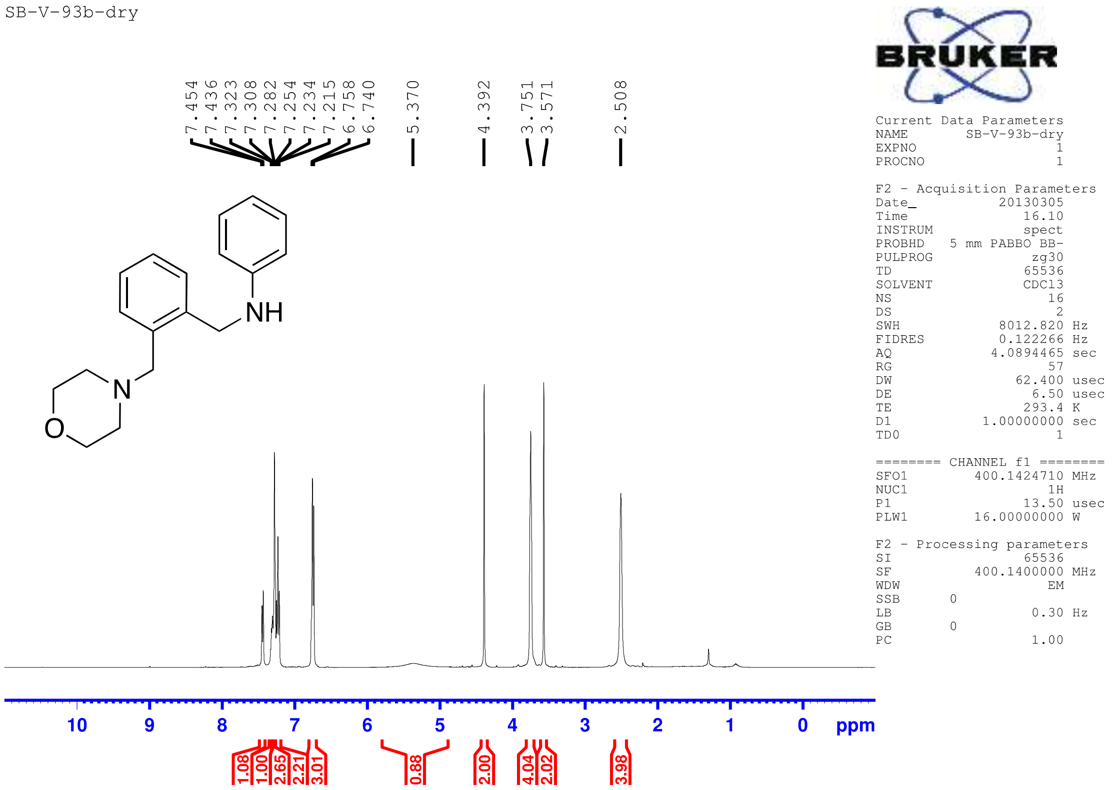

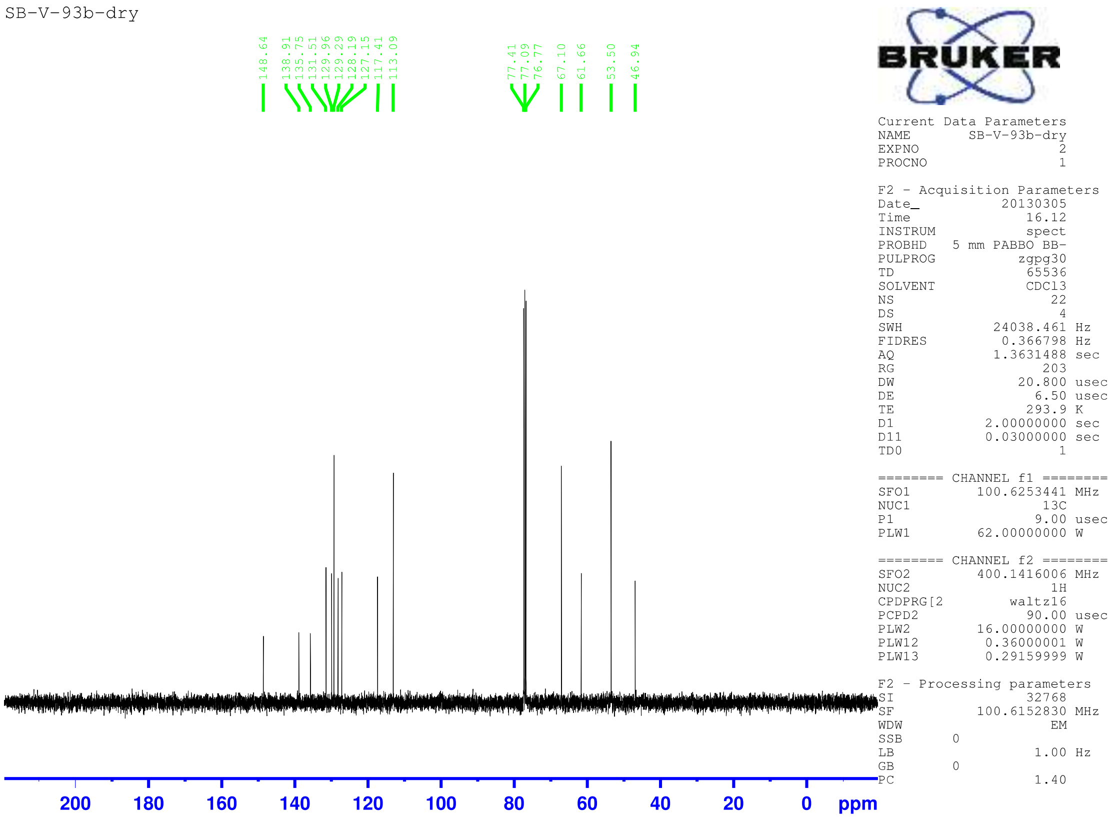

in 90%MeOH **17:58:03 05-Mar-2013**

SARAH_SB-V-93B_BWANG-ACCU_03-05-2013_ESI-POS01 100 (1.988) AM (Cen,2, 80.00, Ar,5000.0,556.28,0.70); Sm (SG, 1.70e4

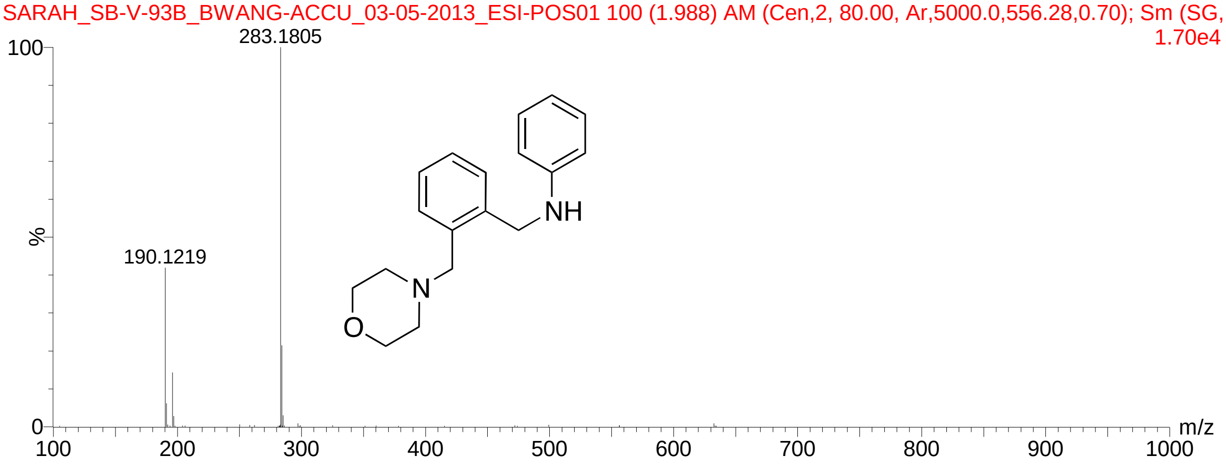

Elemental Composition Report

Single Mass Analysis
Tolerance = 5.0 PPM / DBE: min = -1.5, max = 50.0
Element prediction: Off
Number of isotope peaks used for i-FIT = 3

Monoisotopic Mass, Even Electron Ions
363 formula(e) evaluated with 2 results within limits (up to 100 closest results for each mass)
Elements Used:
C: 1-150 H: 1-150 N: 1-30 O: 1-60

Minimum:				-1.5		
Maximum:		5.0	5.0	50.0		
Mass	Calc. Mass	mDa	PPM	DBE	i-FIT	Formula
283.1805	283.1810	-0.5	-1.8	8.5	12.4	C18 H23 N2 O

N-(4-Morpholinobenzyl)aniline (10d)

ZD-I-75c

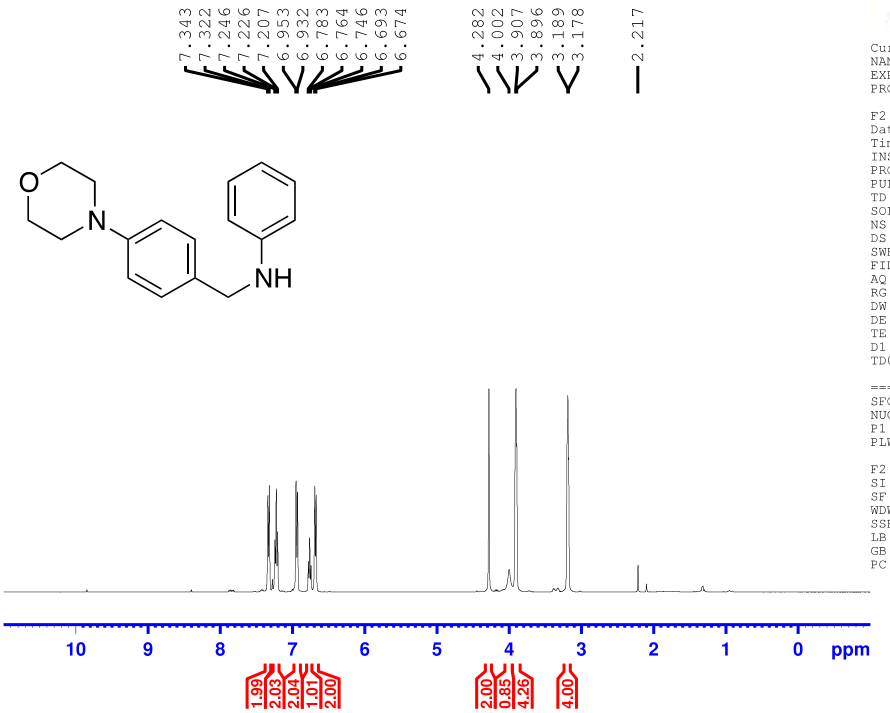


```
Current Data Parameters
NAME             ZD-I-75c
EXPNO                   1
PROCNO                  1

F2 - Acquisition Parameters
Date_            20130212
Time                11.53
INSTRUM             spect
PROBHD   5 mm PABBO BB-
PULPROG              zg30
TD                  65536
SOLVENT             CDCl3
NS                     15
DS                      2
SWH              8012.820 Hz
FIDRES           0.122266 Hz
AQ              4.0894465 sec
RG                   40.3
DW                 62.400 usec
DE                   6.50 usec
TE                  294.0 K
D1             1.00000000 sec
TD0                     1

======== CHANNEL f1 ========
SFO1          400.1424710 MHz
NUC1                   1H
P1                  13.50 usec
PLW1          16.00000000 W

F2 - Processing parameters
SI                  65536
SF            400.1400000 MHz
WDW                    EM
SSB      0
LB                   0.30 Hz
GB       0
PC                   1.00
```

ZD-I-75c

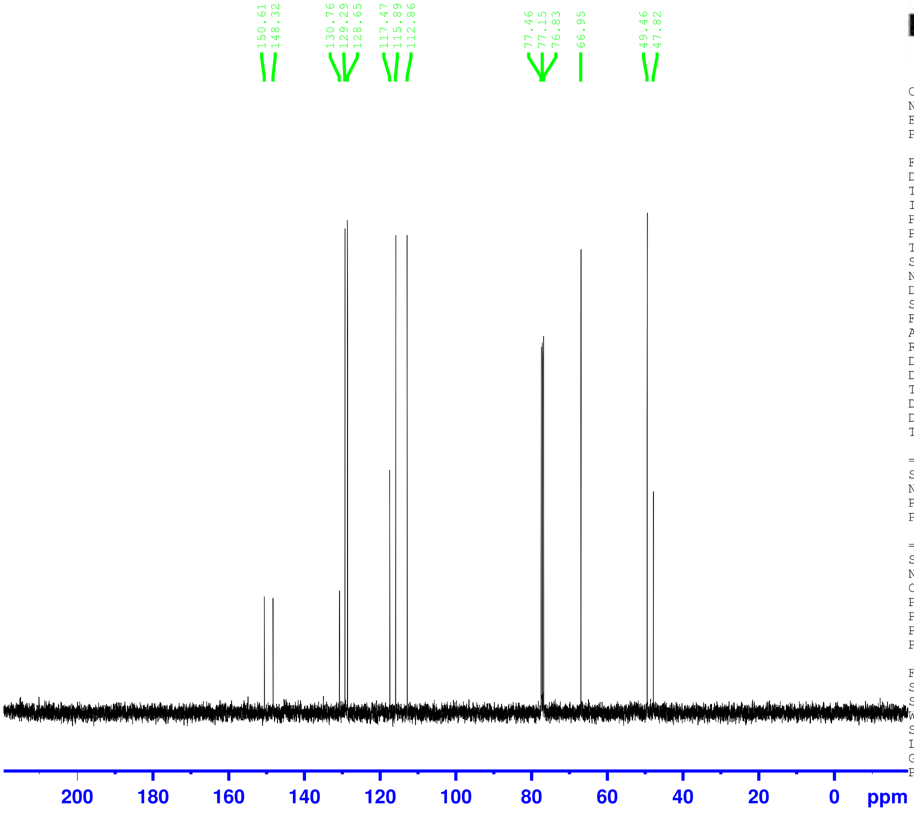


```
Current Data Parameters
NAME             ZD-I-75c
EXPNO                   2
PROCNO                  1

F2 - Acquisition Parameters
Date_            20130212
Time                11.55
INSTRUM             spect
PROBHD   5 mm PABBO BB-
PULPROG            zgpg30
TD                  65536
SOLVENT             CDCl3
NS                     24
DS                      4
SWH             24038.461 Hz
FIDRES           0.366798 Hz
AQ              1.3631488 sec
RG                    203
DW                 20.800 usec
DE                   6.50 usec
TE                  294.5 K
D1             2.00000000 sec
D11            0.03000000 sec
TD0                     1

======== CHANNEL f1 ========
SFO1          100.6253441 MHz
NUC1                  13C
P1                   9.00 usec
PLW1          62.00000000 W

======== CHANNEL f2 ========
SFO2          400.1416006 MHz
NUC2                   1H
CPDPRG[2          waltz16
PCPD2               90.00 usec
PLW2          16.00000000 W
PLW12          0.36000001 W
PLW13          0.29159999 W

F2 - Processing parameters
SI                  32768
SF            100.6152830 MHz
WDW                    EM
SSB      0
LB                   1.00 Hz
GB       0
PC                   1.40
```

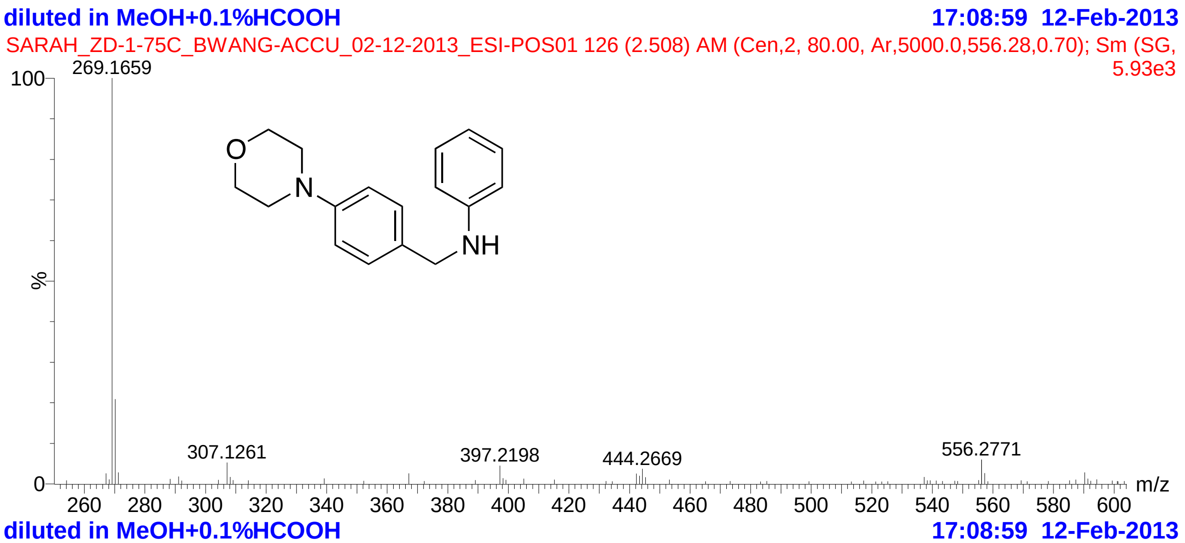

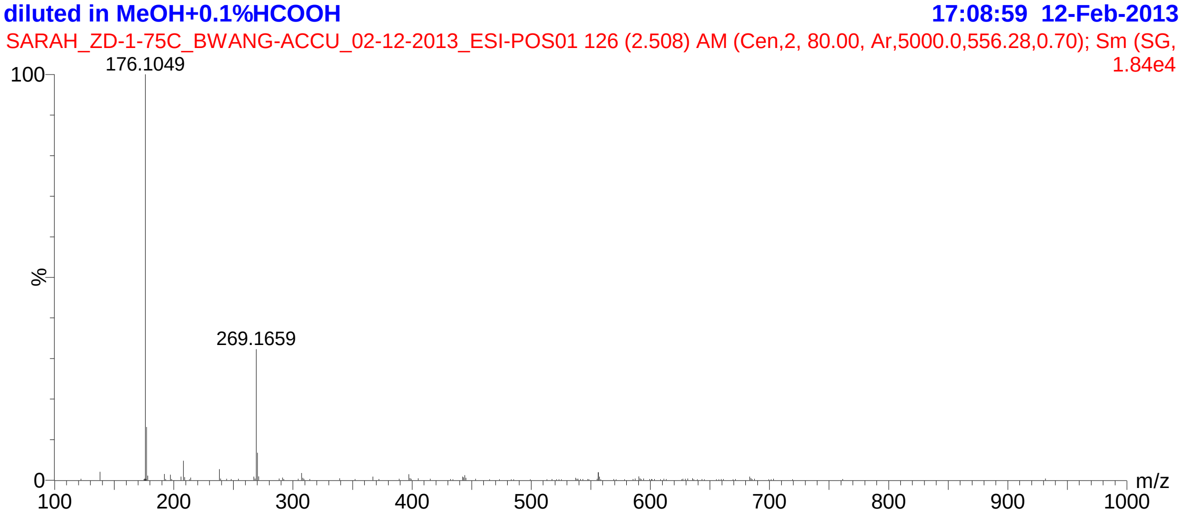

Elemental Composition Report

Single Mass Analysis
Tolerance = 5.0 PPM / DBE: min = -1.5, max = 50.0
Element prediction: Off
Number of isotope peaks used for i-FIT = 3

Monoisotopic Mass, Odd and Even Electron Ions
313 formula(e) evaluated with 3 results within limits (all results (up to 1000) for each mass)
Elements Used:
C: 1-150 H: 1-150 N: 1-30 O: 1-30

Minimum:				-1.5			
Maximum:		5.0	5.0	50.0			
Mass	Calc. Mass	mDa		PPM		DBE	i-FIT	Formula
269.1659	269.1654	0.5	1.9	8.5	n/a	C17H21N2O	

N-(4-(Morpholinomethyl)benzyl)cyclobutanamine (11a)

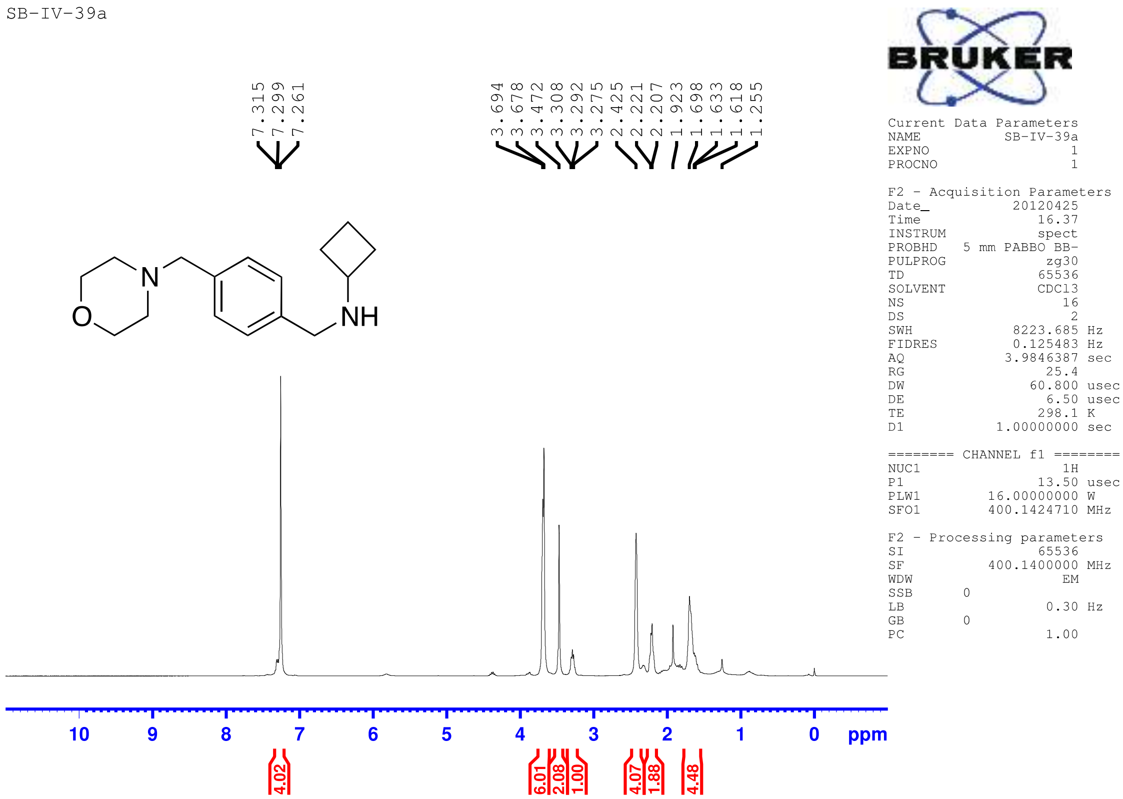

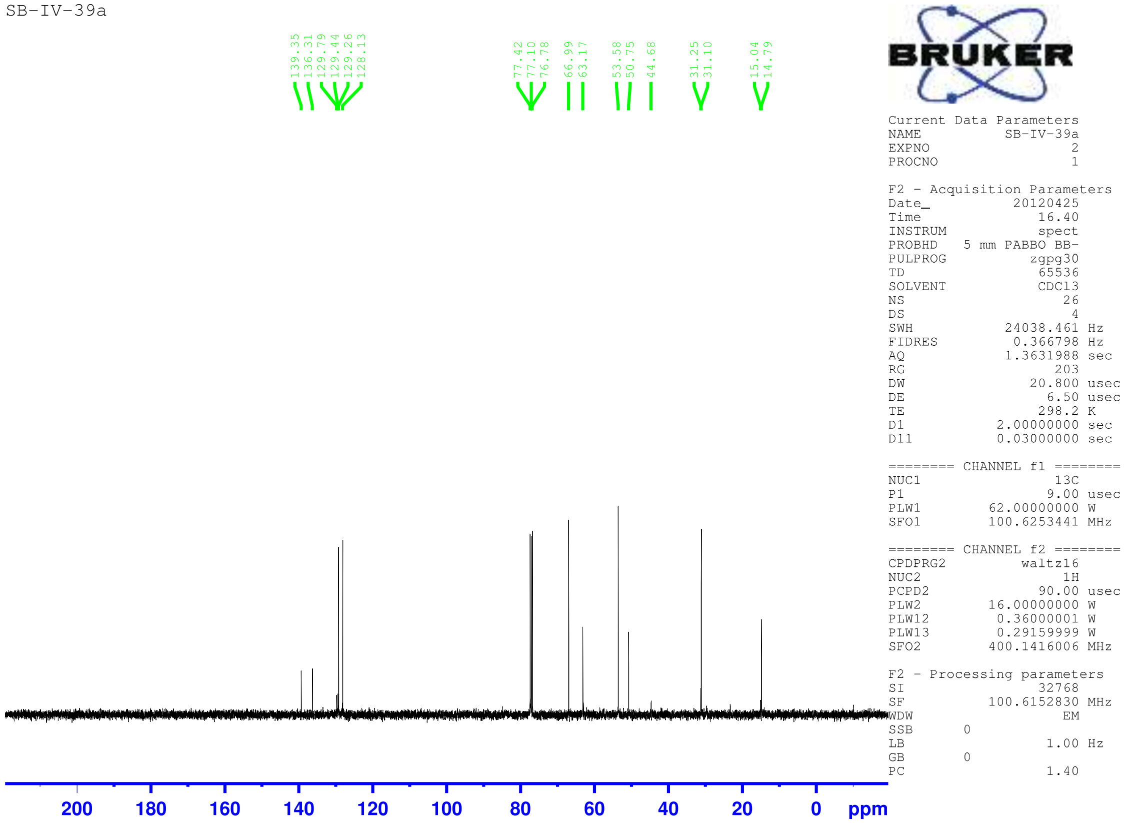

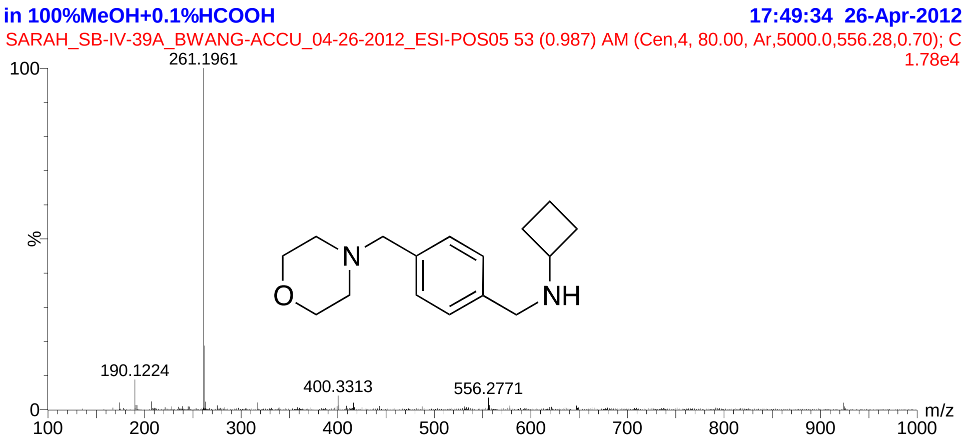

Elemental Composition Report

Single Mass Analysis
Tolerance = 5.0 PPM / DBE: min = -1.5, max = 50.0
Element prediction: Off
Number of isotope peaks used for i-FIT = 3

Monoisotopic Mass, Even Electron Ions
284 formula(e) evaluated with 2 results within limits (all results (up to 1000) for each mass)
Elements Used:
C: 1-100 H: 1-100 N: 1-15 O: 1-100

Minimum:				-1.5		
Maximum:		5.0	5.0	50.0		
Mass	Calc. Mass	mDa	PPM	DBE	i-FIT	Formula
261.1961	261.1967	-0.6	-2.3	5.5	7.3	C16 H25 N2 O

N-(3-(Morpholinomethyl)benzyl)cyclobutanamine (11b)

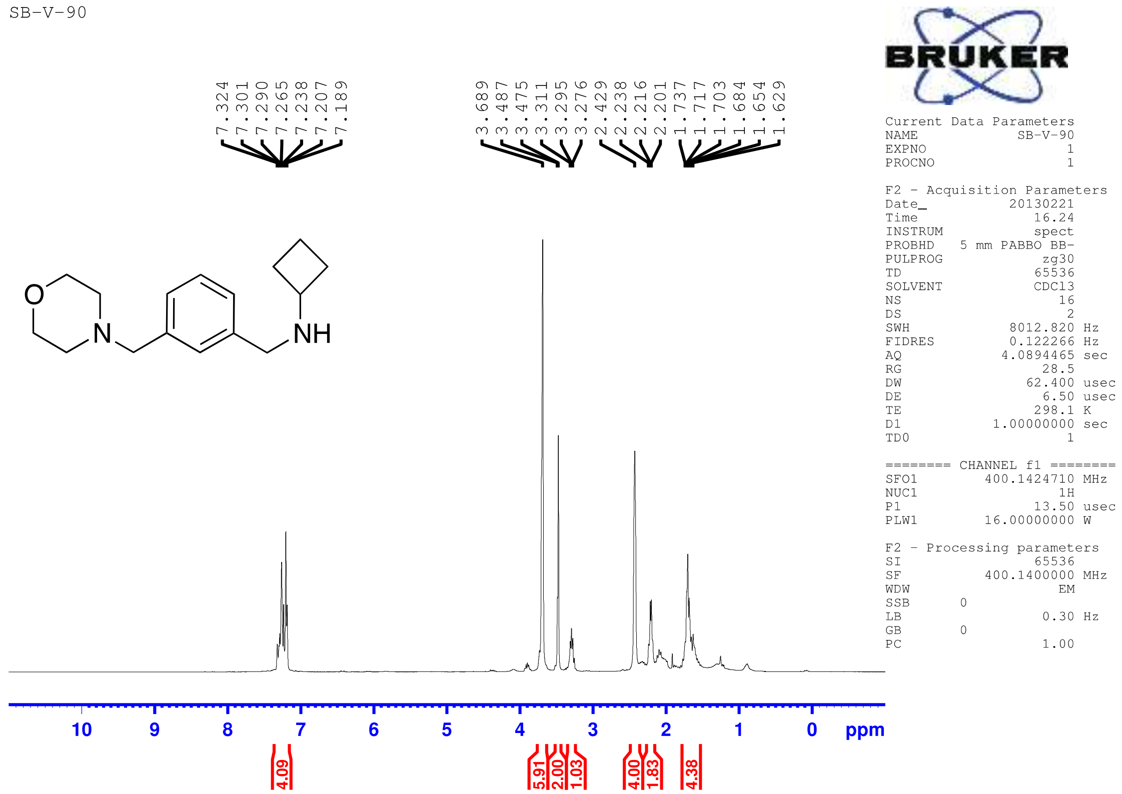

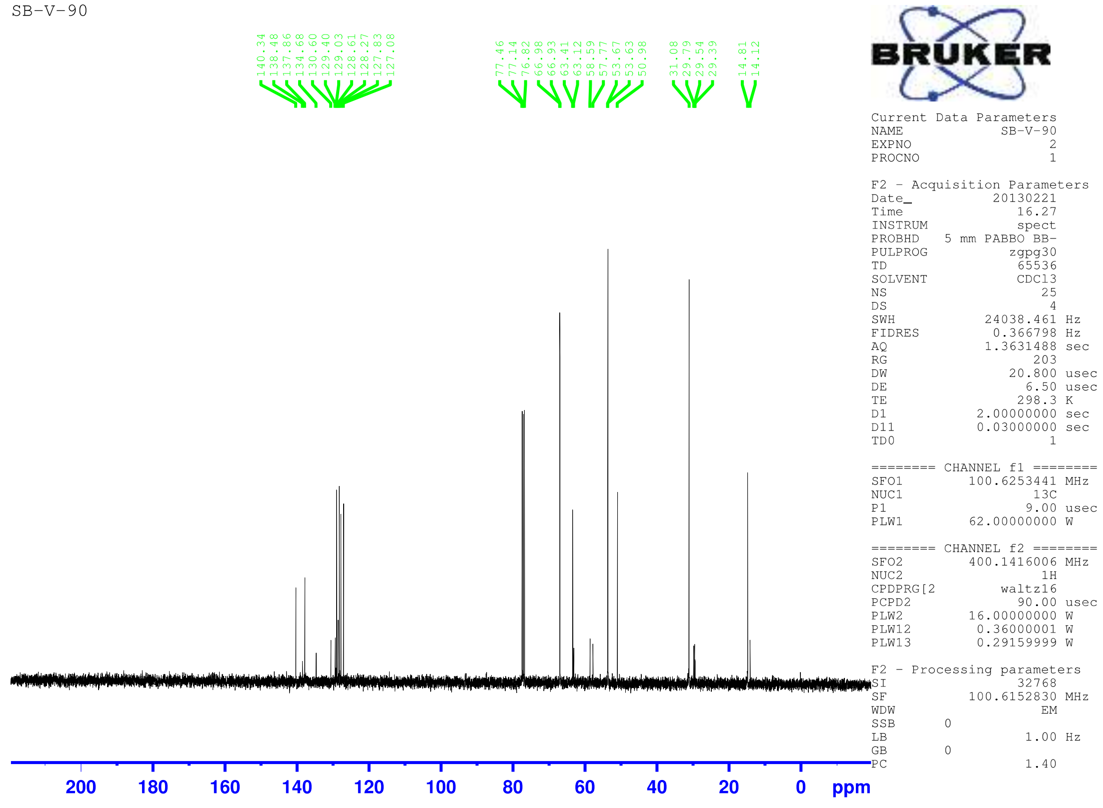

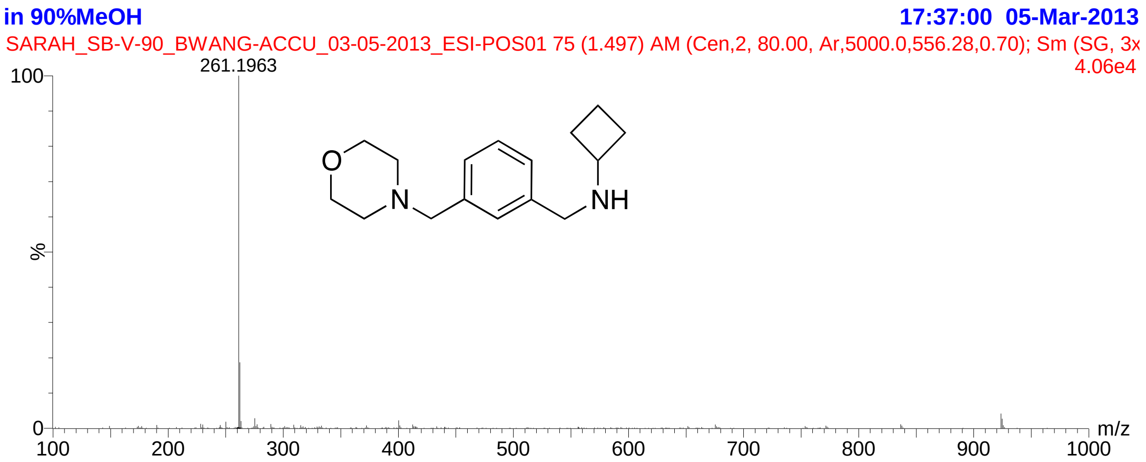

Elemental Composition Report

Single Mass Analysis
Tolerance = 5.0 PPM / DBE: min = -1.5, max = 50.0
Element prediction: Off
Number of isotope peaks used for i-FIT = 3

Monoisotopic Mass, Even Electron Ions
286 formula(e) evaluated with 2 results within limits (up to 100 closest results for each mass)
Elements Used:
C: 1-150 H: 1-150 N: 1-30 O: 1-60

Minimum:				-1.5		
Maximum:		5.0	5.0	50.0		
Mass	Calc. Mass	mDa	PPM	DBE	i-FIT	Formula
261.1963	261.1967	-0.4	-1.5	5.5	2.5	C16 H25 N2 O

N-(2-(Morpholinomethyl)benzyl)cyclobutanamine (11c)

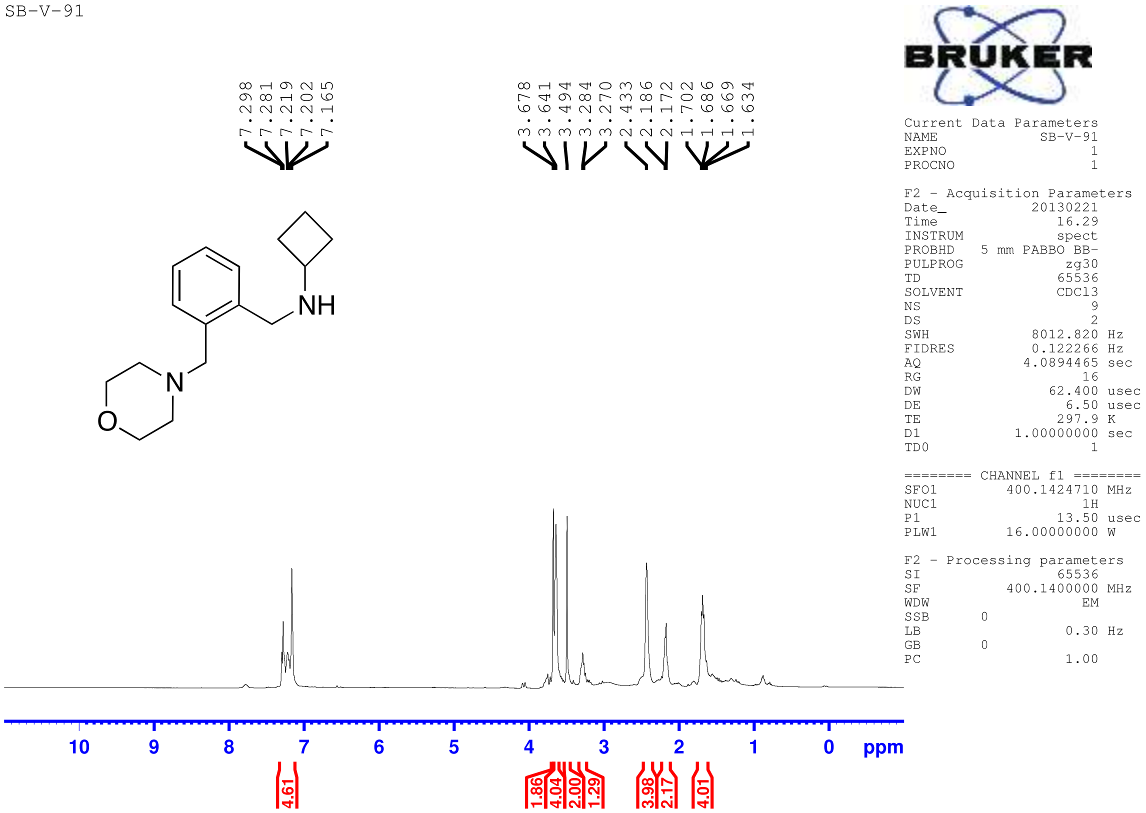

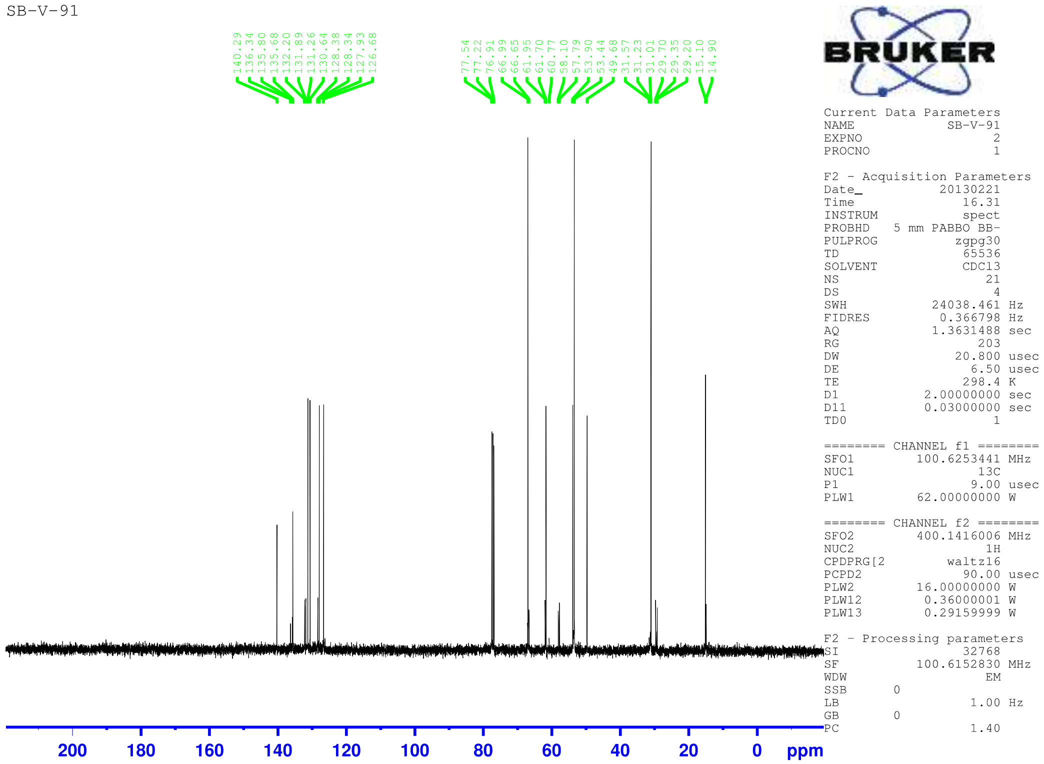

in 90%MeOH 18:08:53 05-Mar-2013
SARAH_SB-V-91_BWANG-ACCU_03-05-2013_ESI-POS01 175 (3.491) AM (Cen,2, 80.00, Ar,5000.0,556.28,0.70); Sm (SG, 3

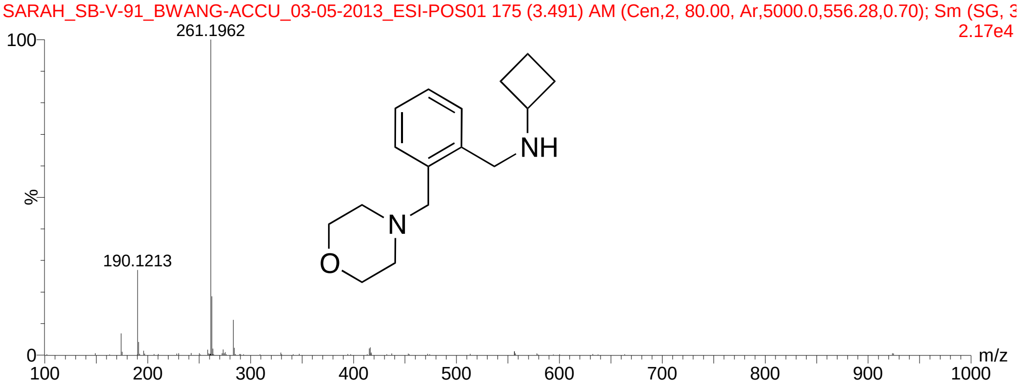

Elemental Composition Report

Single Mass Analysis
Tolerance = 5.0 PPM / DBE: min = -1.5, max = 50.0
Element prediction: Off
Number of isotope peaks used for i-FIT = 3

Monoisotopic Mass, Even Electron Ions
286 formula(e) evaluated with 2 results within limits (up to 100 closest results for each mass)
Elements Used:
C: 1-150 H: 1-150 N: 1-30 O: 1-60

Minimum:				-1.5			
Maximum:		5.0	5.0	50.0			
Mass	Calc. Mass	mDa	PPM	DBE	i-FIT	Formula	
261.1962	261.1967	-0.5	-1.9	5.5	1.6	C16 H25 N2 O	

N-(4-Morpholinobenzyl)cyclobutanamine (11d)

MeOH 14:20:04 26-Feb-2013

SARAH_SB-V-87_BWANG-ACCU_02-26-2013_ESI-POS01 79 (1.573) AM (Cen,2, 80.00, Ar,5000.0,556.28,0.70); Sm (SG, 3x

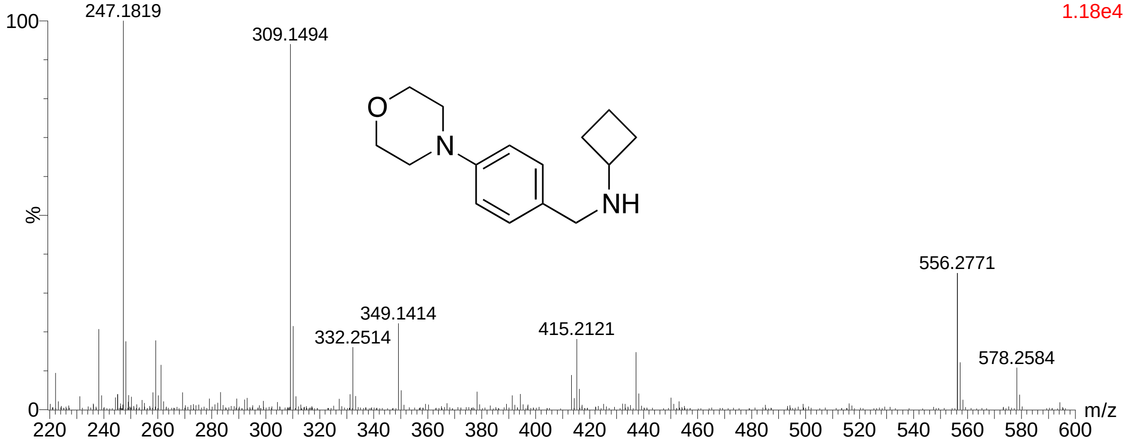

Elemental Composition Report

Single Mass Analysis
Tolerance = 5.0 PPM / DBE: min = -1.5, max = 50.0
Element prediction: Off
Number of isotope peaks used for i-FIT = 3

Monoisotopic Mass, Even Electron Ions
153 formula(e) evaluated with 1 results within limits (up to 100 closest results for each mass)
Elements Used:
C: 1-150 H: 1-150 N: 1-6 O: 1-30

Minimum:				-1.5		
Maximum:		5.0	5.0	50.0		
Mass	Calc. Mass	mDa	PPM	DBE	i-FIT	Formula
247.1819	247.1810	0.9	3.6	5.5	70.6	C15 H23 N2 O

2,2-Dimethyl-2*H*-chromene-6-carbaldehyde (12)

JH-I-50ca

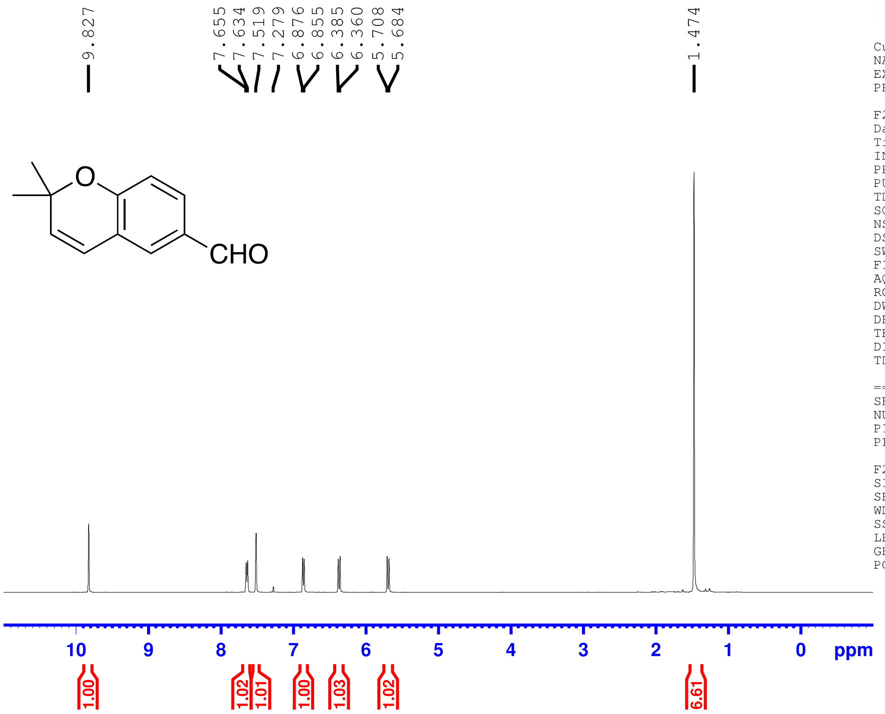

BRUKER

```
Current Data Parameters
NAME             JH-I-50ca
EXPNO                    1
PROCNO                   1

F2 - Acquisition Parameters
Date_             20130118
Time                  9.45
INSTRUM              spect
PROBHD   5 mm PABBO BB-
PULPROG               zg30
TD                   65536
SOLVENT              CDCl3
NS                      16
DS                       2
SWH               8012.820 Hz
FIDRES            0.122266 Hz
AQ               4.0894465 sec
RG                    50.8
DW                  62.400 usec
DE                    6.50 usec
TE                   293.8 K
D1              1.00000000 sec
TD0                      1

======== CHANNEL f1 ========
SFO1           400.1424710 MHz
NUC1                    1H
P1                   13.50 usec
PLW1           16.00000000 W

F2 - Processing parameters
SI                   65536
SF             400.1400000 MHz
WDW                     EM
SSB      0
LB                    0.30 Hz
GB       0
PC                    1.00
```

N-((2,2-Dimethyl-2*H*-chromen-6-yl)methyl)-2-morpholinoethanamine (13a)

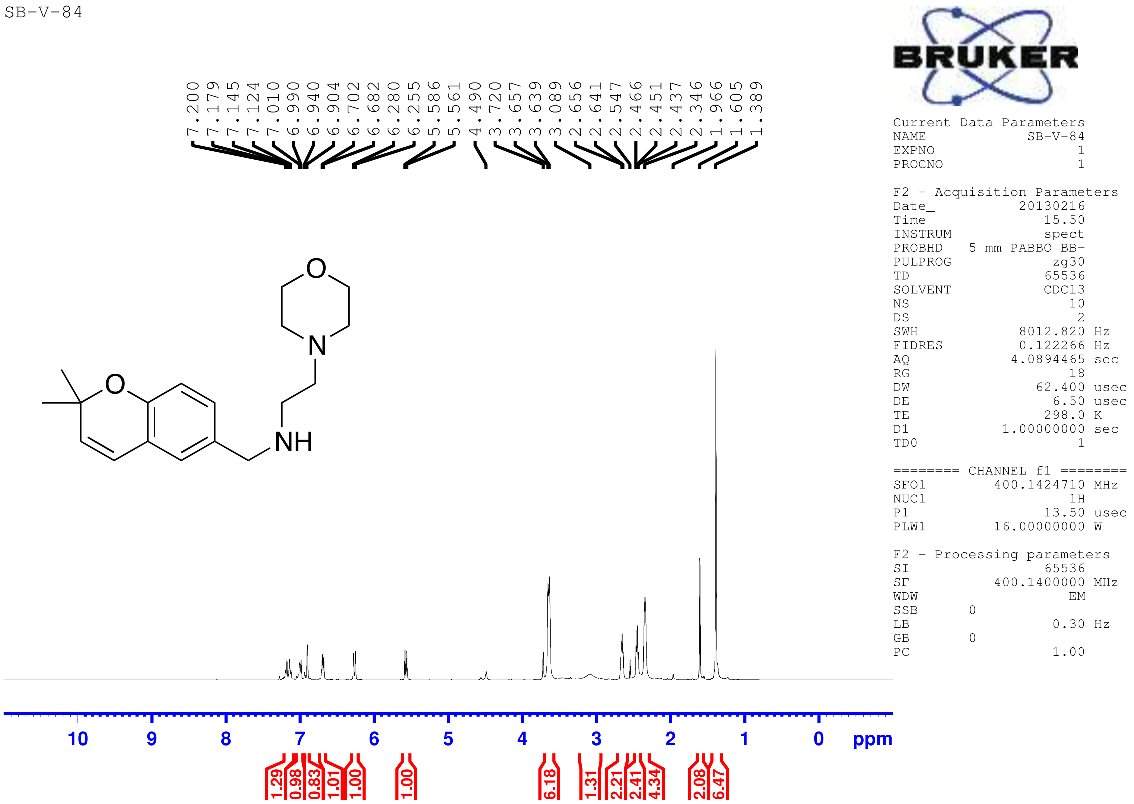

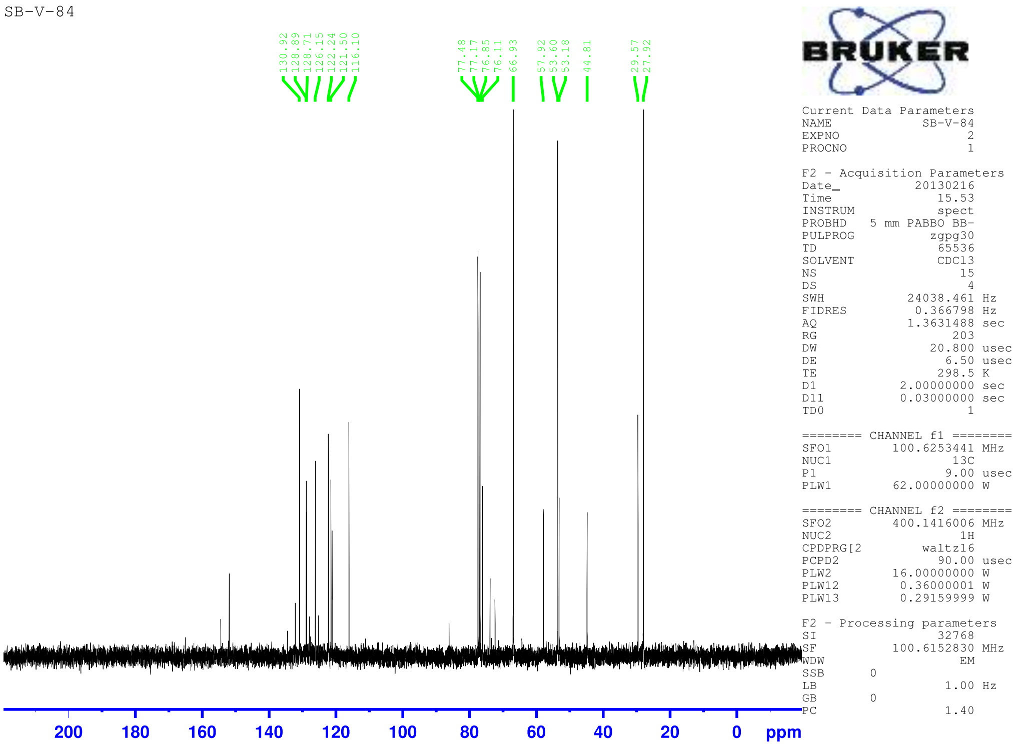

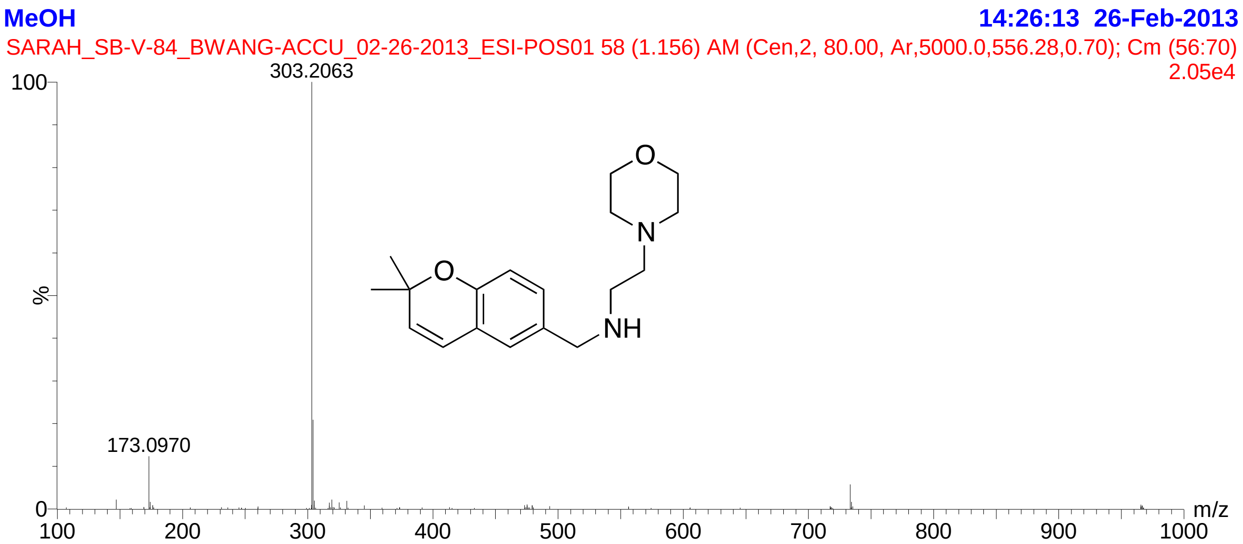

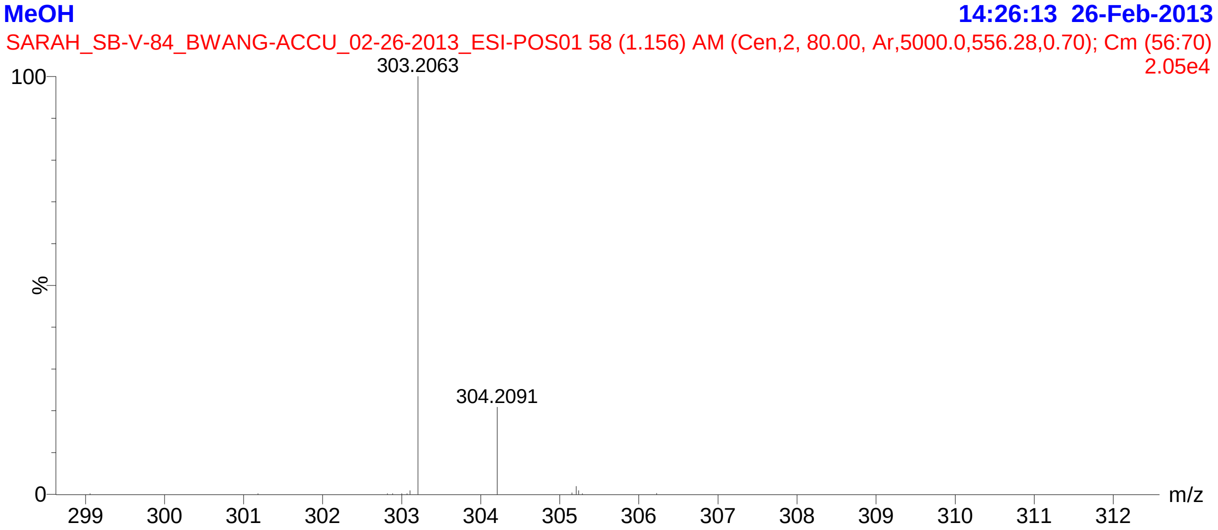

Elemental Composition Report

Single Mass Analysis
Tolerance = 5.0 PPM / DBE: min = -1.5, max = 50.0
Element prediction: Off
Number of isotope peaks used for i-FIT = 3

Monoisotopic Mass, Even Electron Ions
234 formula(e) evaluated with 1 results within limits (up to 100 closest results for each mass)
Elements Used:
C: 1-150 H: 1-150 N: 1-6 O: 1-30

Minimum:					-1.5		
Maximum:		5.0	5.0	50.0			
Mass	Calc. Mass	mDa	PPM	DBE	i-FIT	Formula	
303.2063	303.2073	-1.0	-3.3	6.5	22.6	C18 H27 N2 O2	

N-((2,2-Dimethyl-2*H*-chromen-6-yl)methyl)-3-morpholinopropan-1-amine (13b)

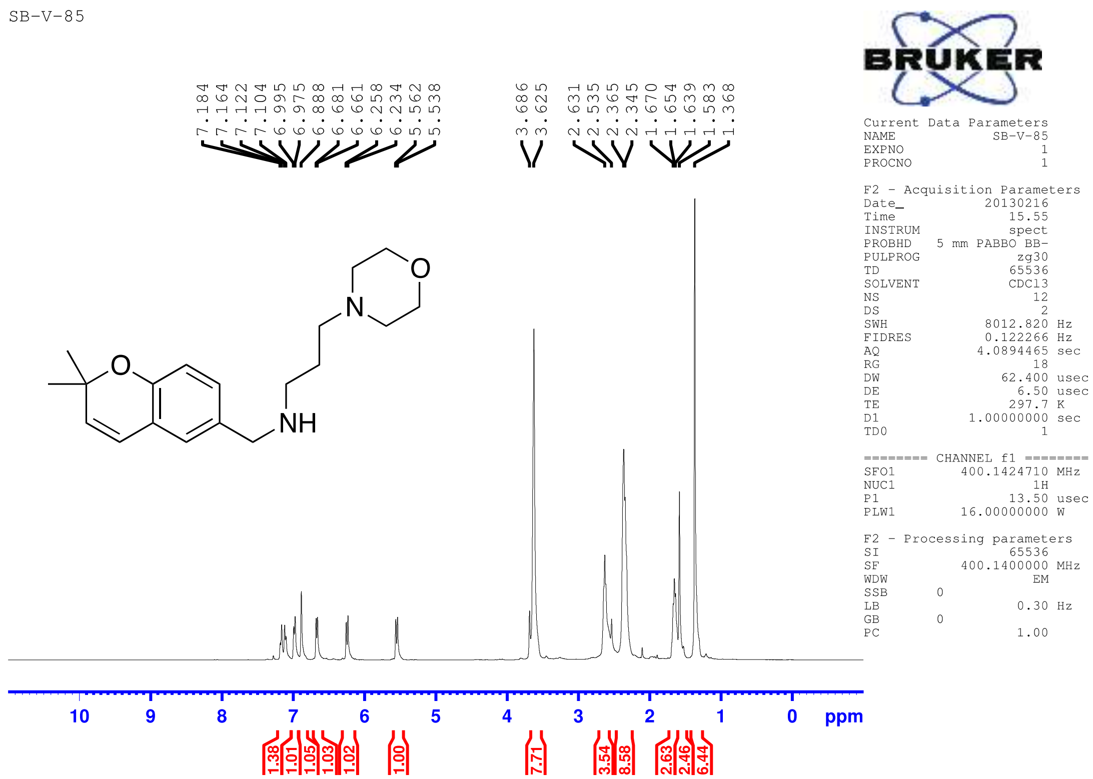

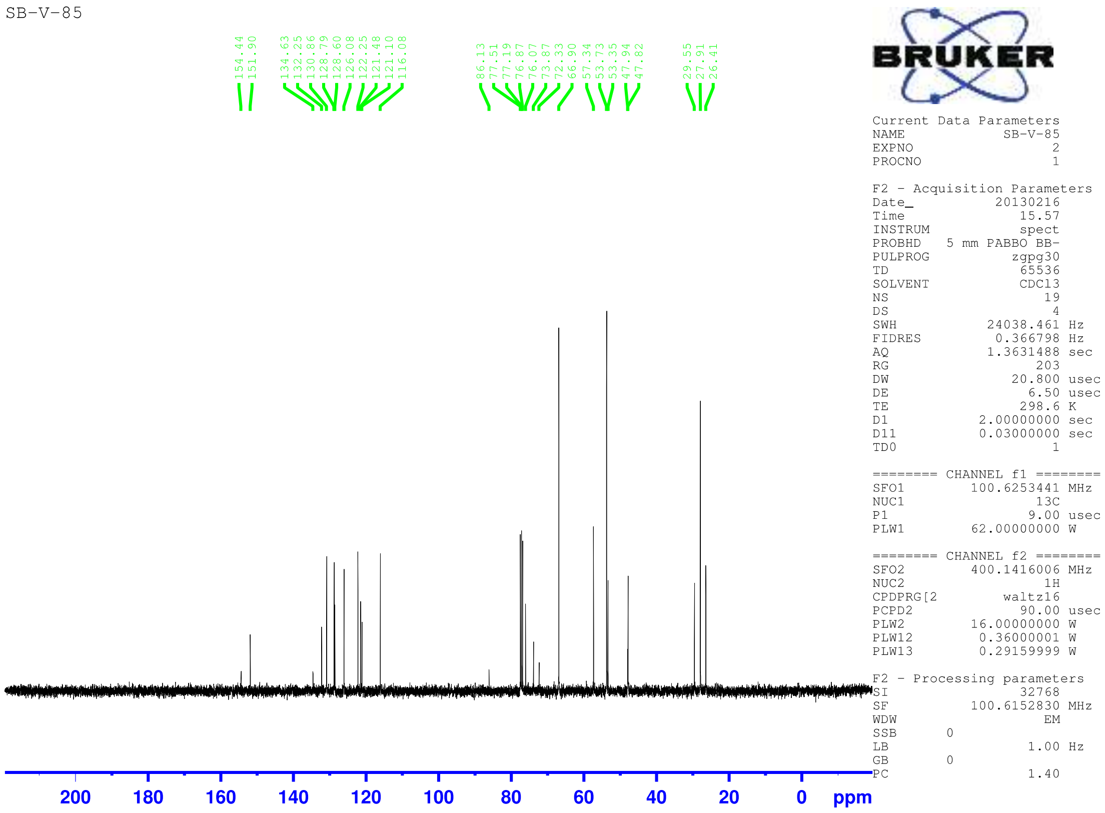

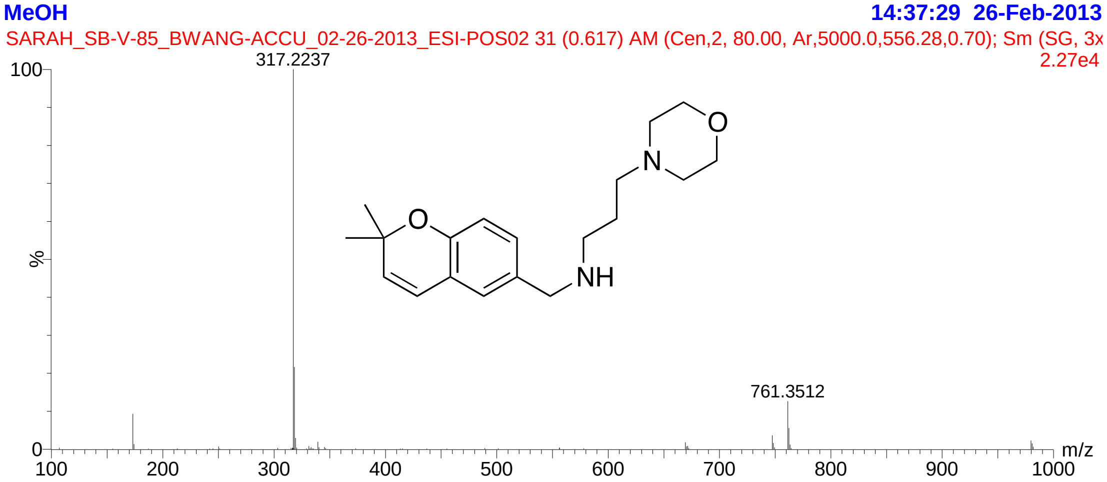

Elemental Composition Report

Single Mass Analysis
Tolerance = 5.0 PPM / DBE: min = -1.5, max = 50.0
Element prediction: Off
Number of isotope peaks used for i-FIT = 3

Monoisotopic Mass, Even Electron Ions
255 formula(e) evaluated with 1 results within limits (up to 100 closest results for each mass)
Elements Used:
C: 1-150 H: 1-150 N: 1-6 O: 1-30

	Mass	Calc. Mass	mDa	PPM	DBE	i-FIT	Formula
Minimum:					-1.5		
Maximum:			5.0	5.0	50.0		
	317.2237	317.2229	0.8	2.5	6.5	3.5	C19 H29 N2 O2

N-((2,2-Dimethyl-2*H*-chromen-6-yl)methyl)aniline (14a)

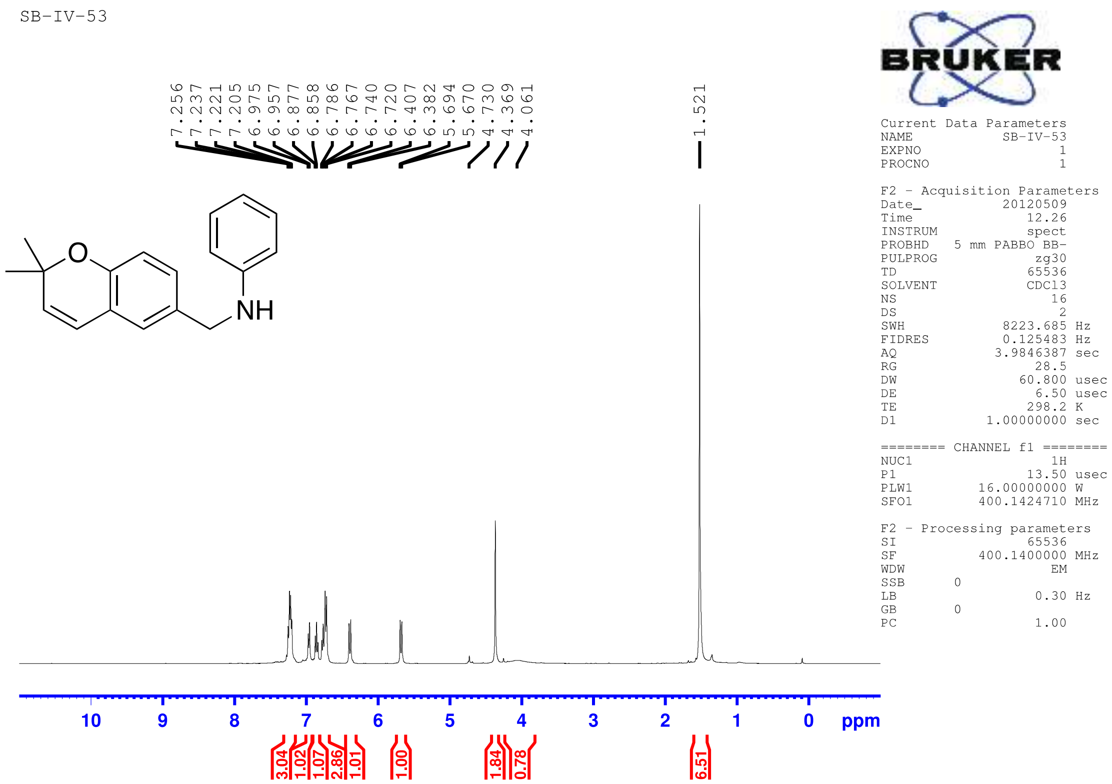

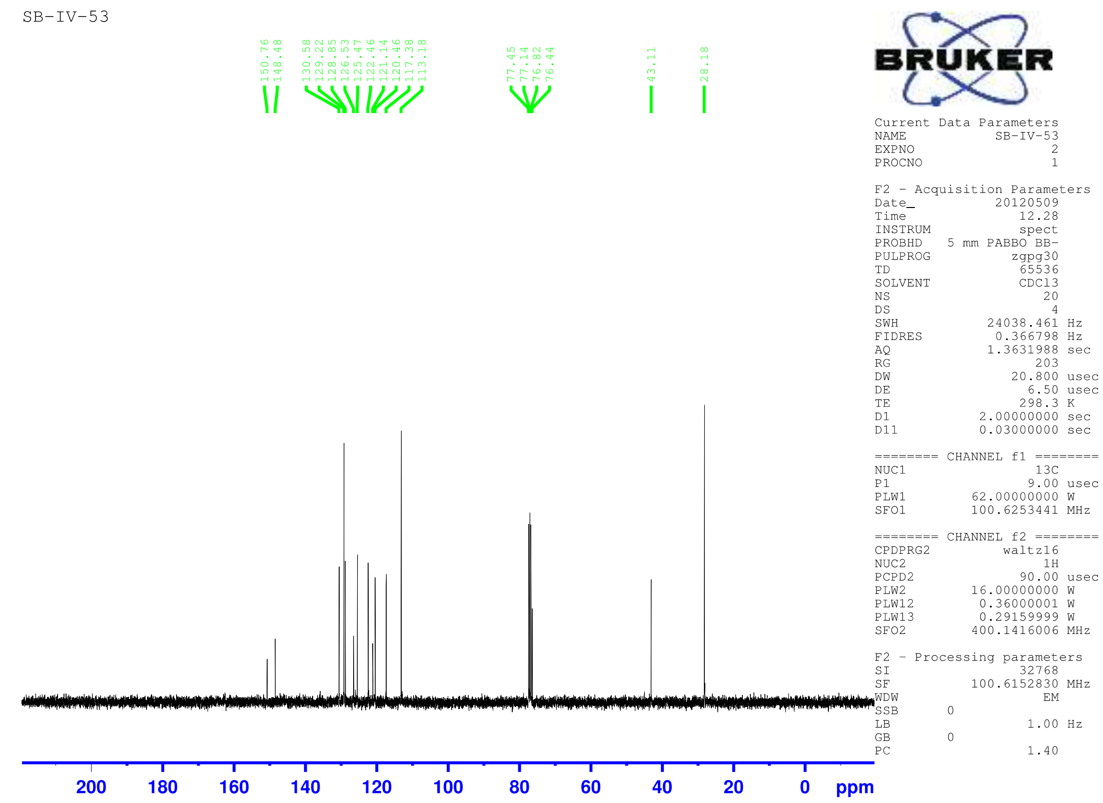

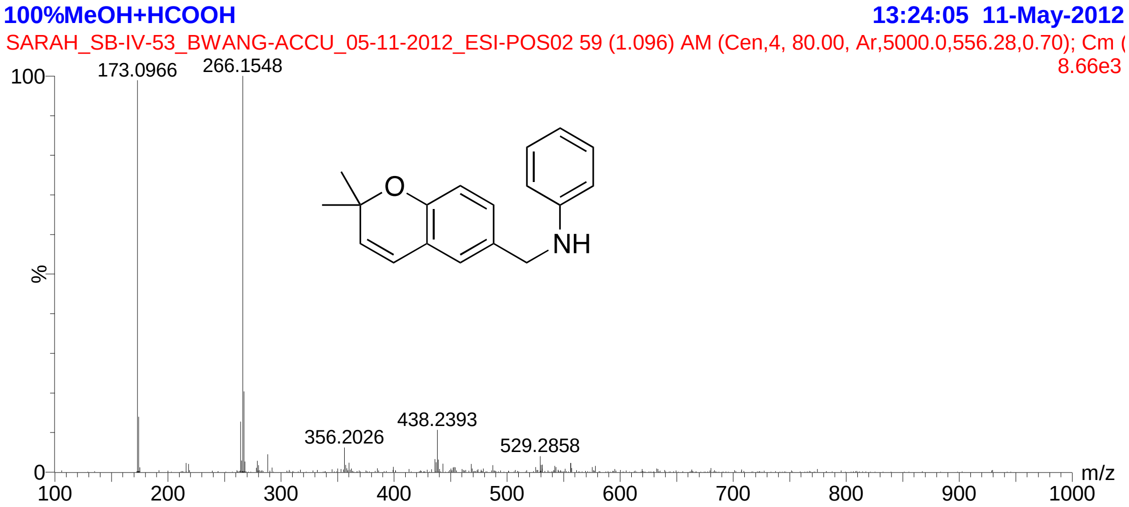

Elemental Composition Report

Single Mass Analysis
Tolerance = 5.0 PPM / DBE: min = -1.5, max = 50.0
Element prediction: Off
Number of isotope peaks used for i-FIT = 3

Monoisotopic Mass, Even Electron Ions
293 formula(e) evaluated with 2 results within limits (all results (up to 1000) for each mass)
Elements Used:
C: 1-100 H: 1-100 N: 1-15 O: 1-100

Minimum:				-1.5		
Maximum:		5.0	5.0	50.0		
Mass	Calc. Mass	mDa	PPM	DBE	i-FIT	Formula
266.1548	266.1545	0.3	1.1	9.5	3.1	C18 H20 N O

N-((2,2-Dimethyl-2*H*-chromen-6-yl)methyl)cyclobutanamine (14b)

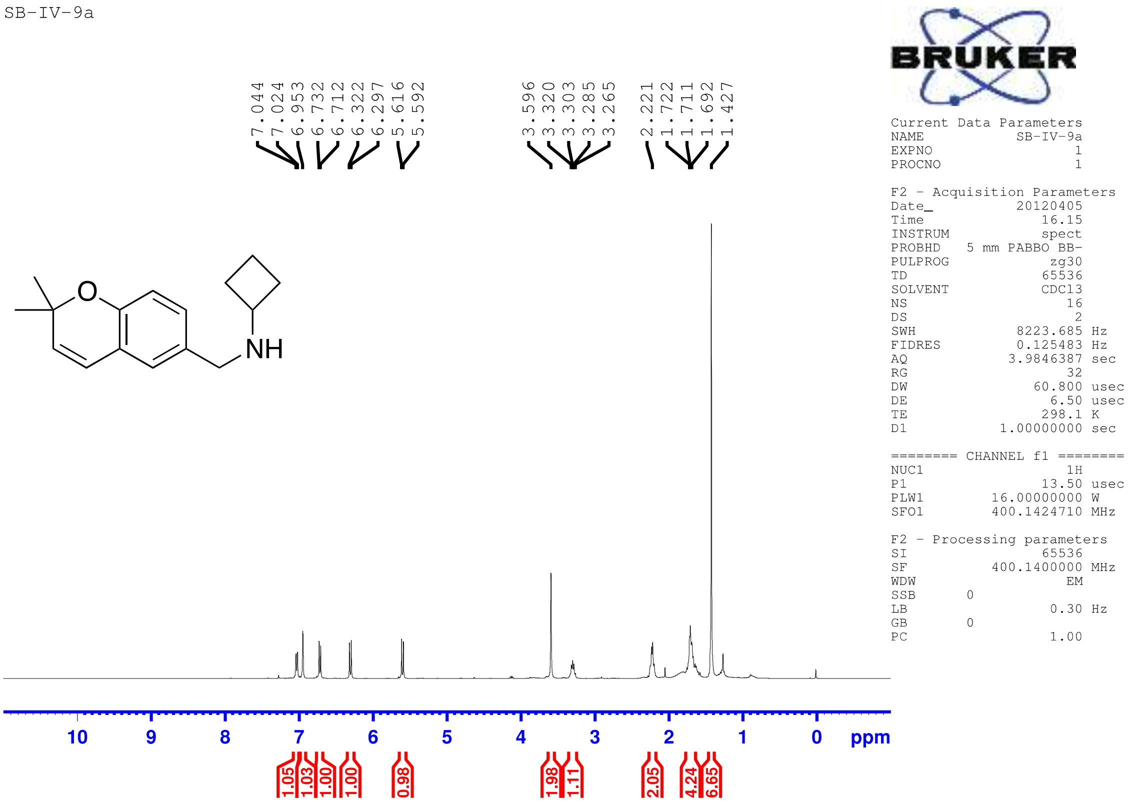

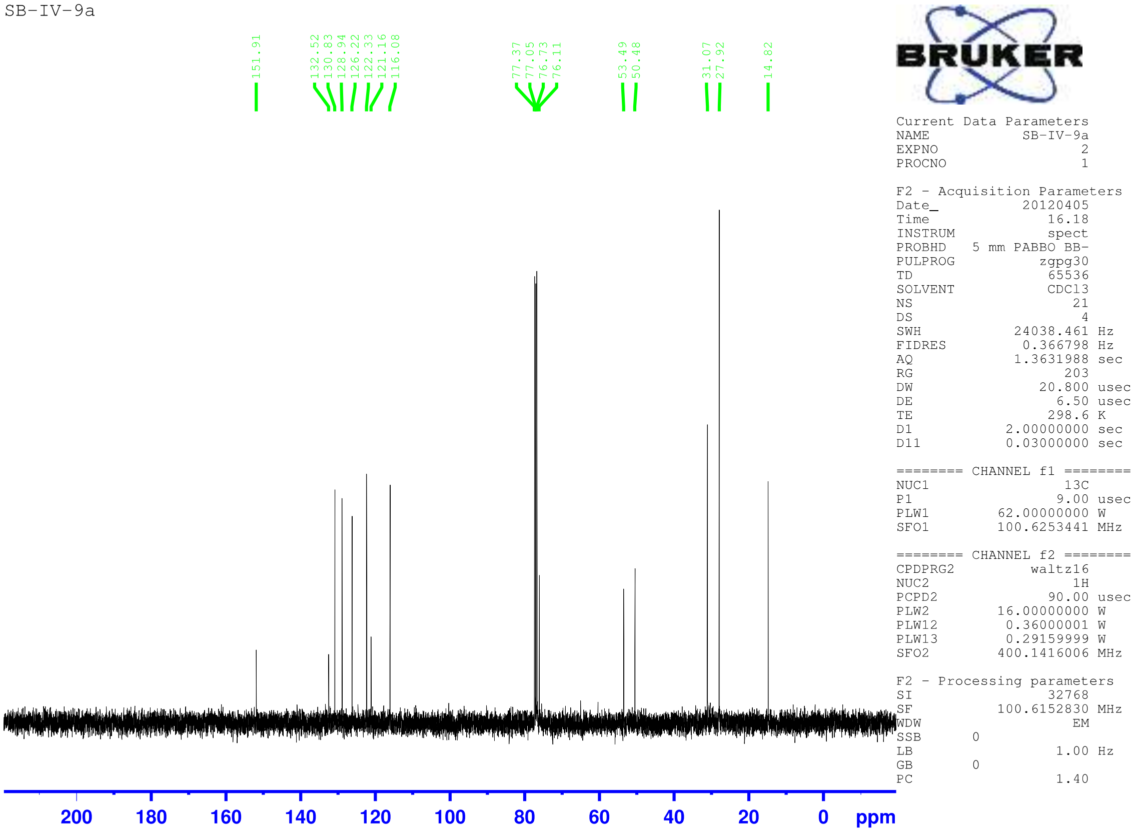

100%MeOH+0.1%HCOOH 15:32:23 13-Apr-2012
SARAH_SB-IV-9a_BWANG-ACCU_04-13-2012_ESI-POS03 120 (2.224) AM (Cen,4, 80.00, Ar,5000.0,556.28,0.70)

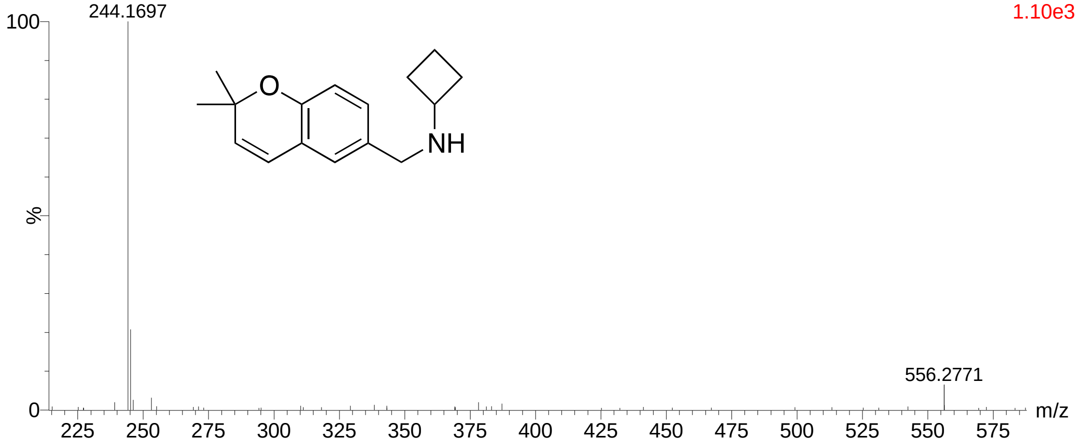

Elemental Composition Report

Single Mass Analysis
Tolerance = 5.0 PPM / DBE: min = -1.5, max = 50.0
Element prediction: Off
Number of isotope peaks used for i-FIT = 3

Monoisotopic Mass, Even Electron Ions
227 formula(e) evaluated with 2 results within limits (all results (up to 1000) for each mass)
Elements Used:
C: 1-100 H: 1-100 N: 1-15 O: 1-20

Minimum:				-1.5		
Maximum:		5.0	5.0	50.0		
Mass	Calc. Mass	mDa	PPM	DBE	i-FIT	Formula
244.1697	244.1701	-0.4	-1.6	6.5	2.7	C16 H22 N O

Graphical Representations of calculated log*D* and log*S* values for **3a-6b**

3a

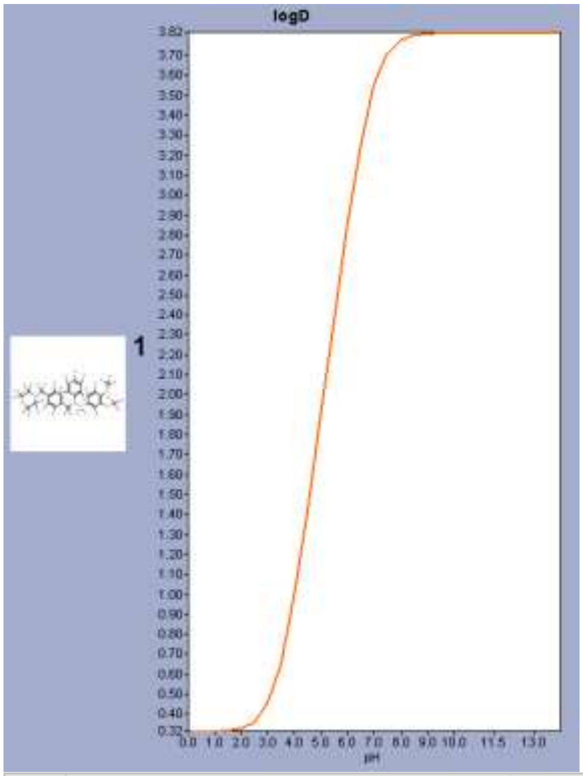

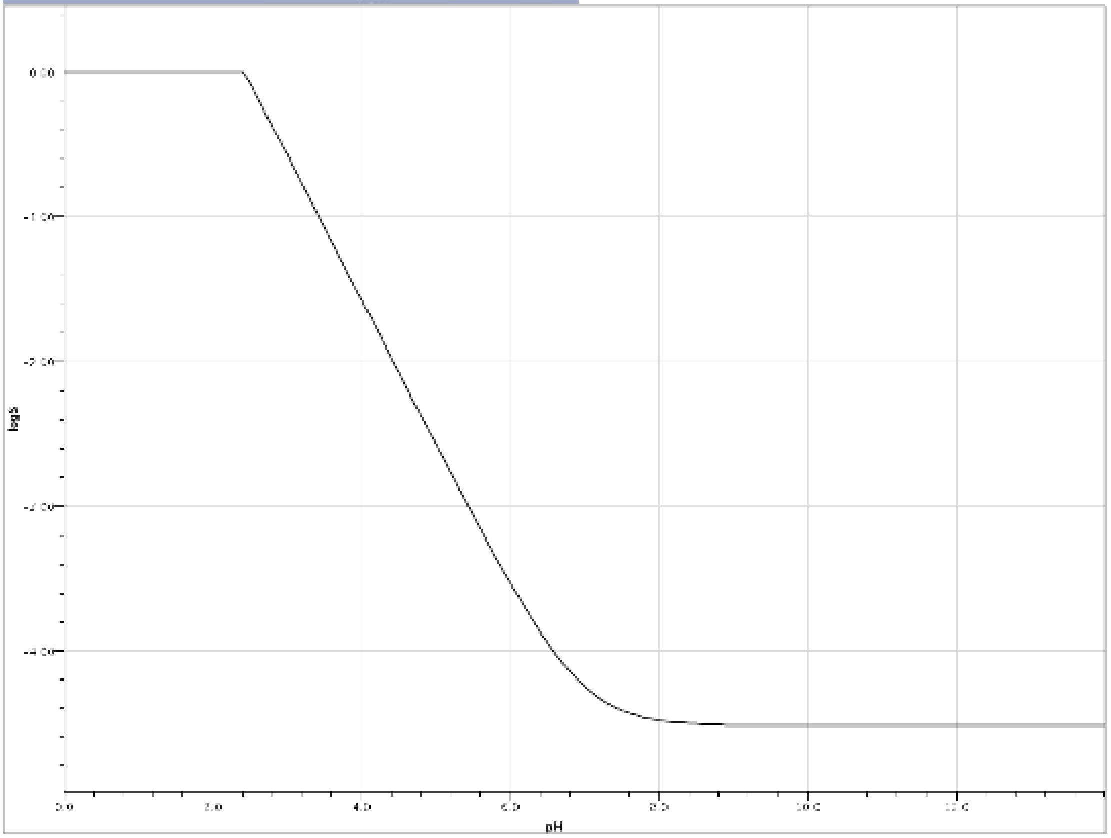

3b

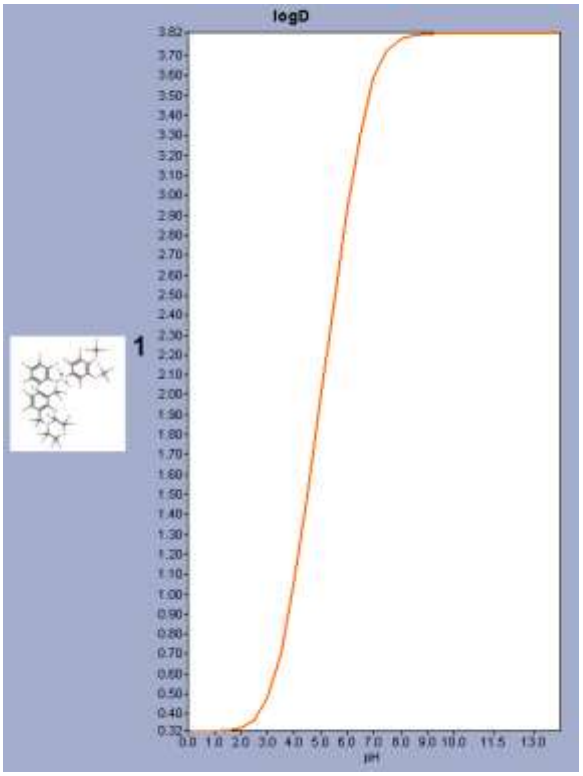

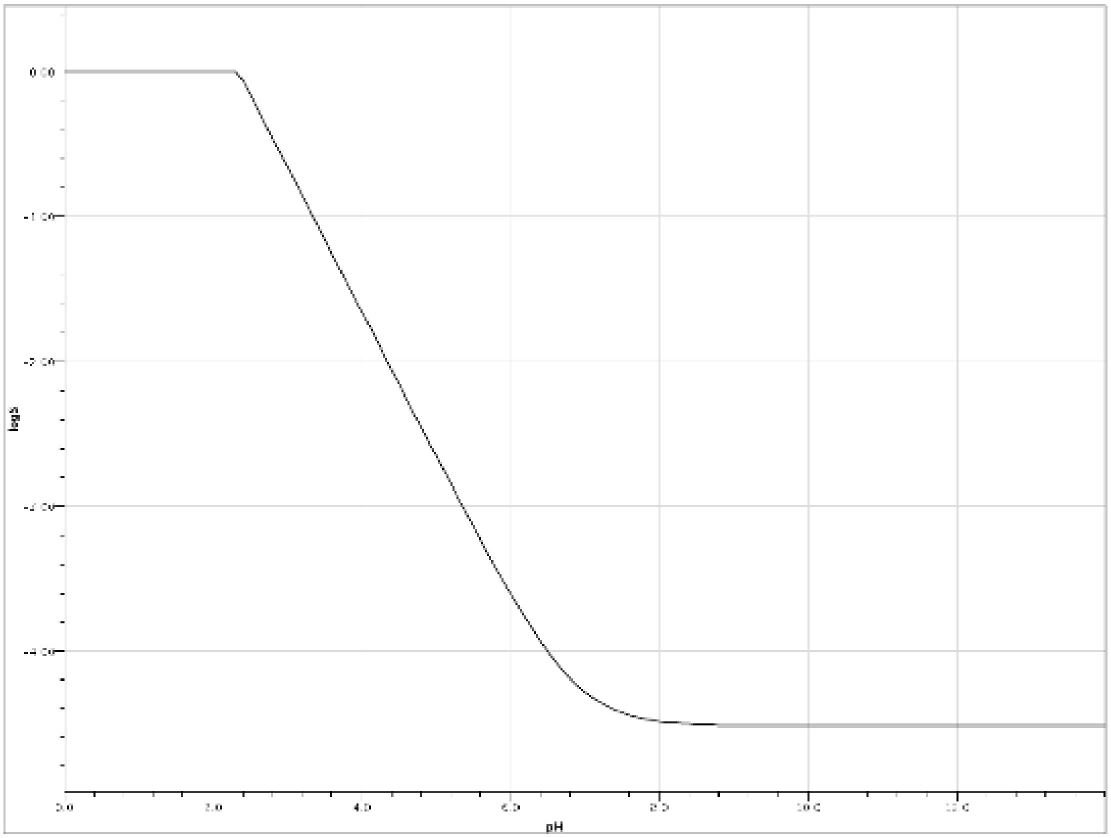

3c

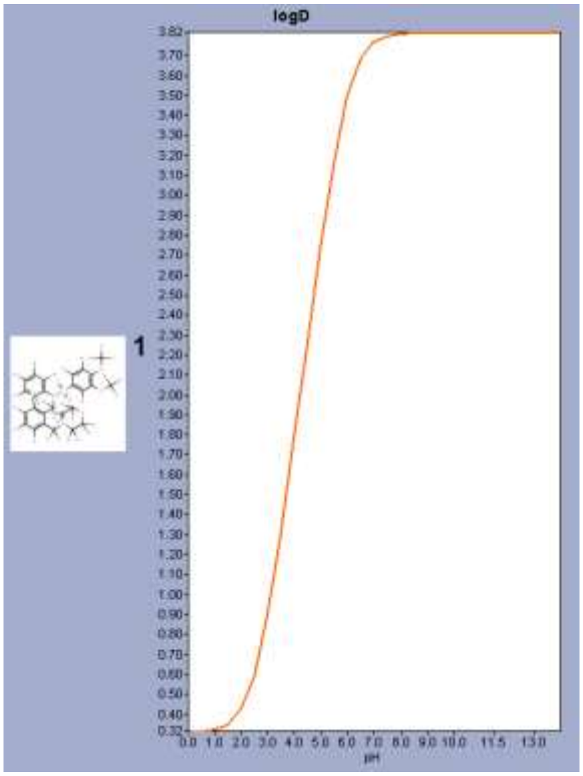

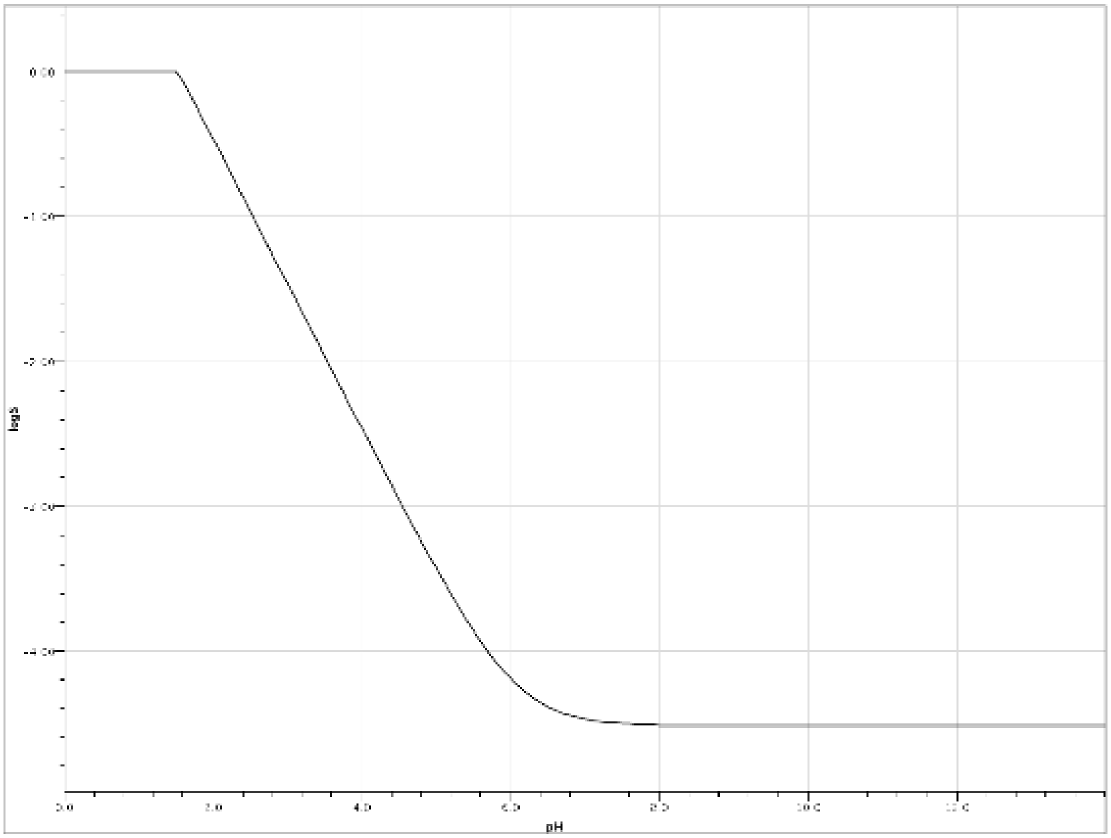

3d

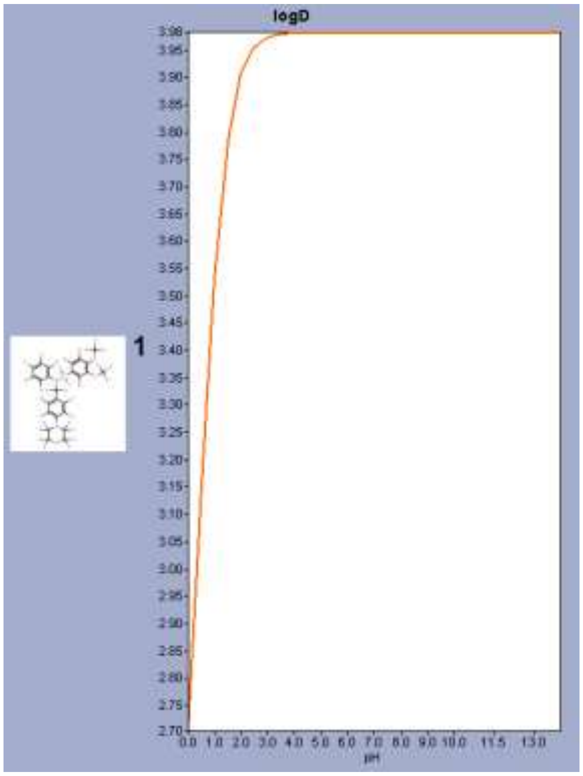

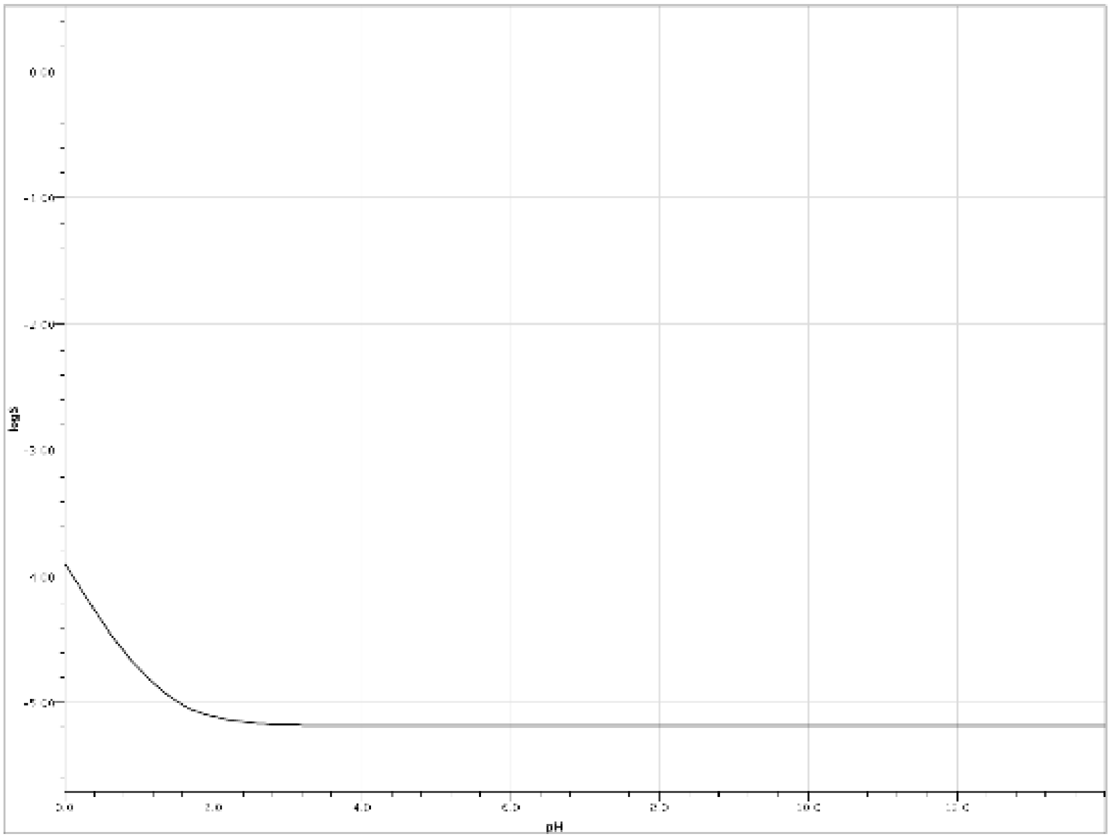

4a

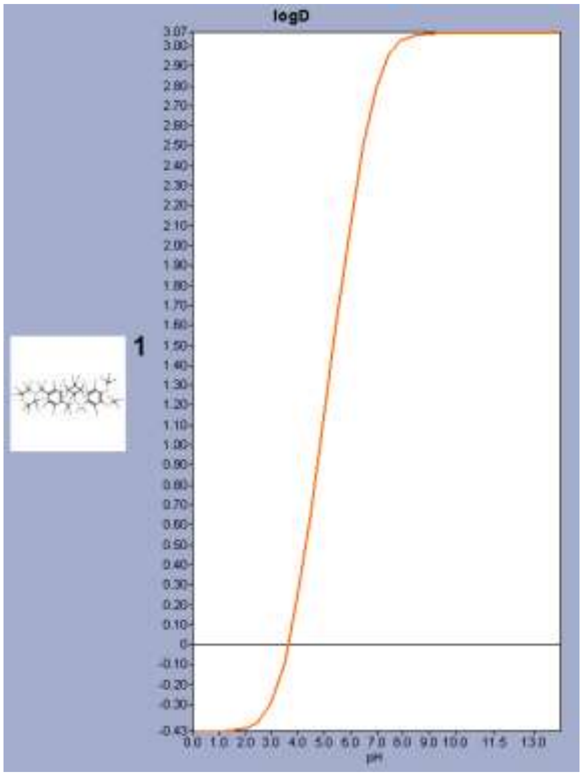

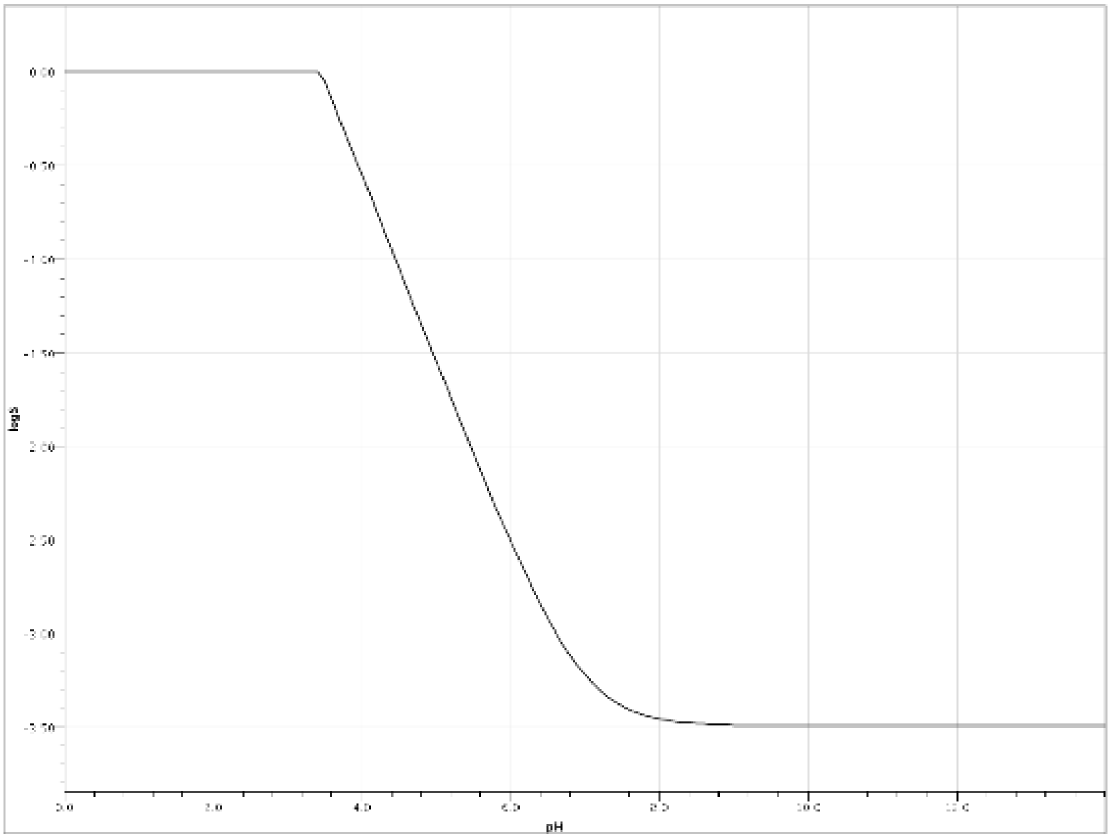

4b

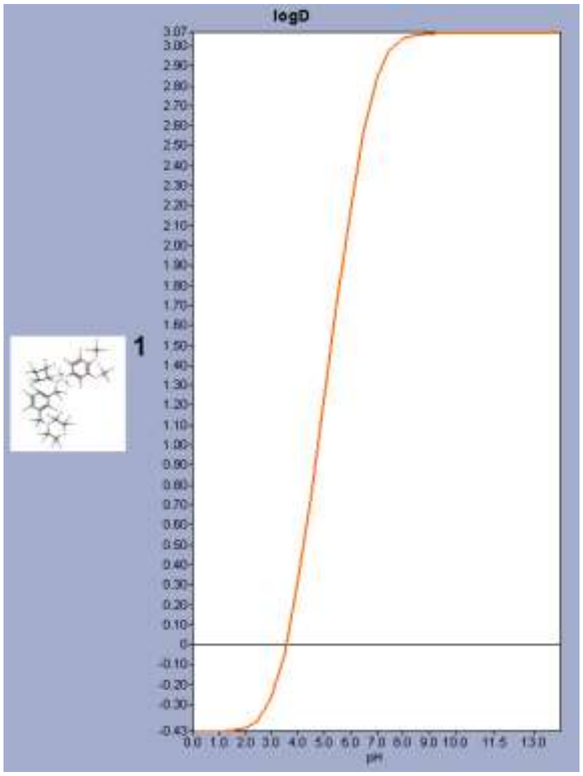

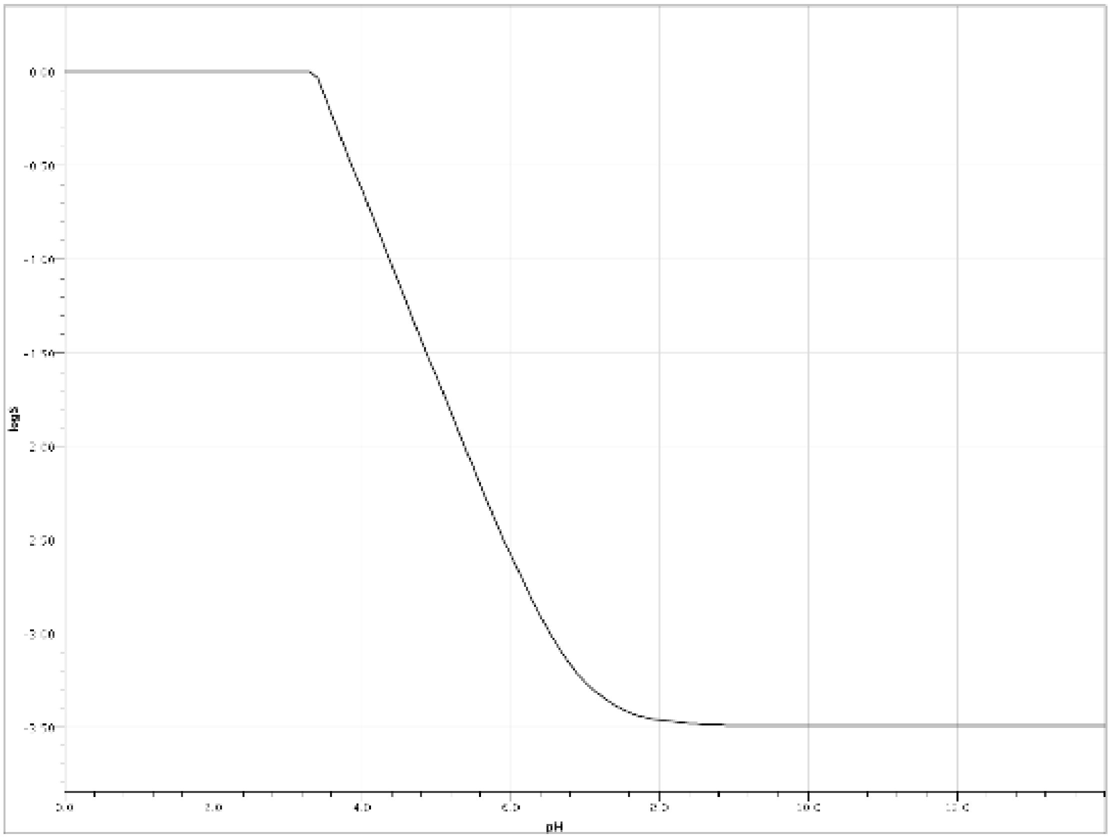

4c

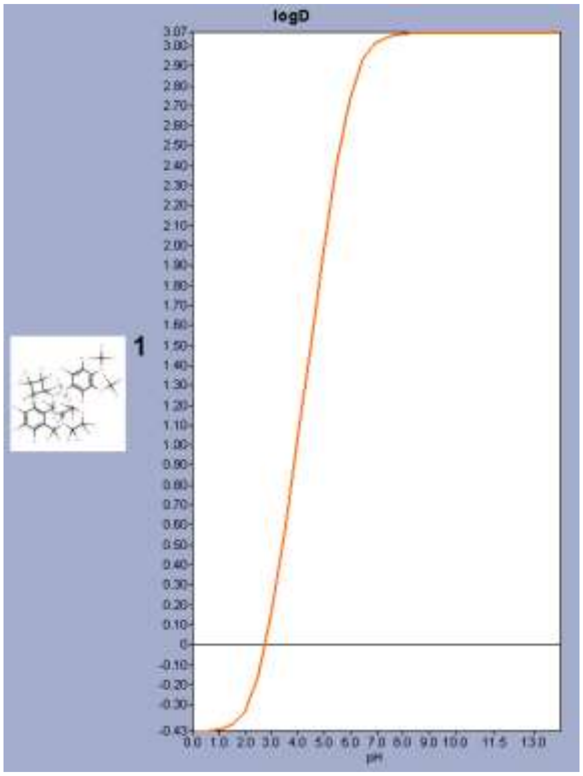

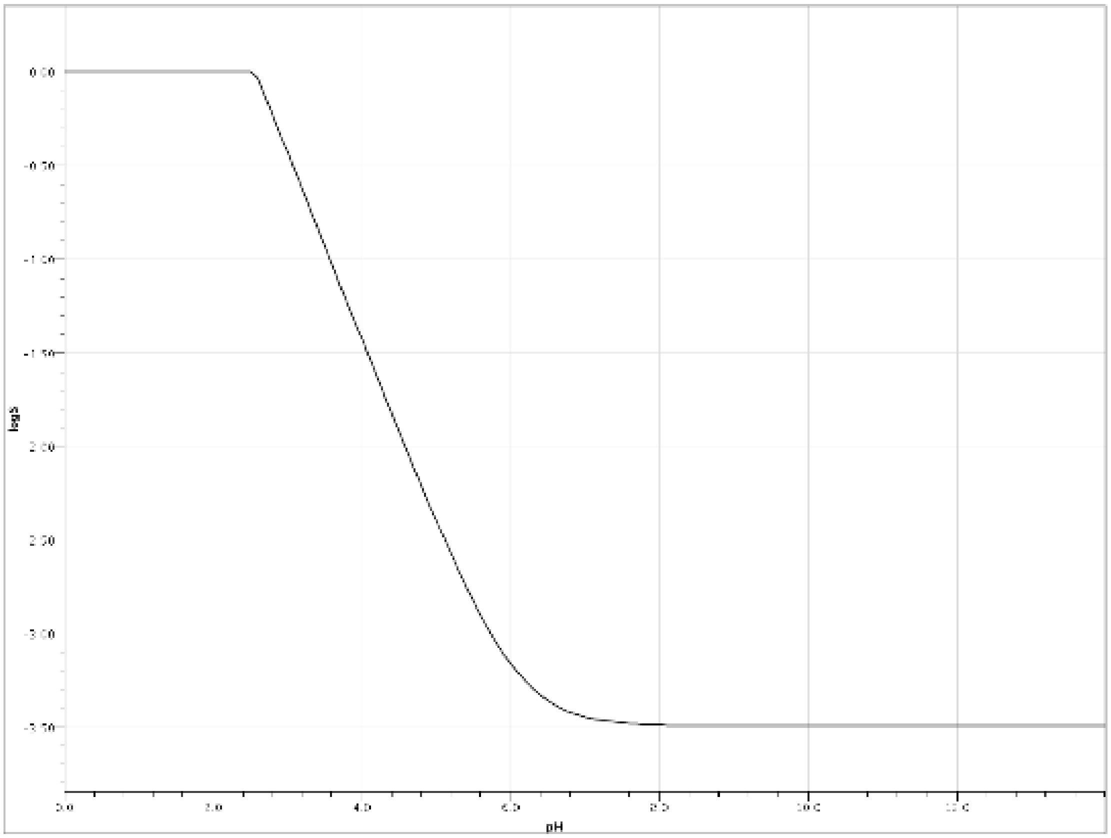

4d

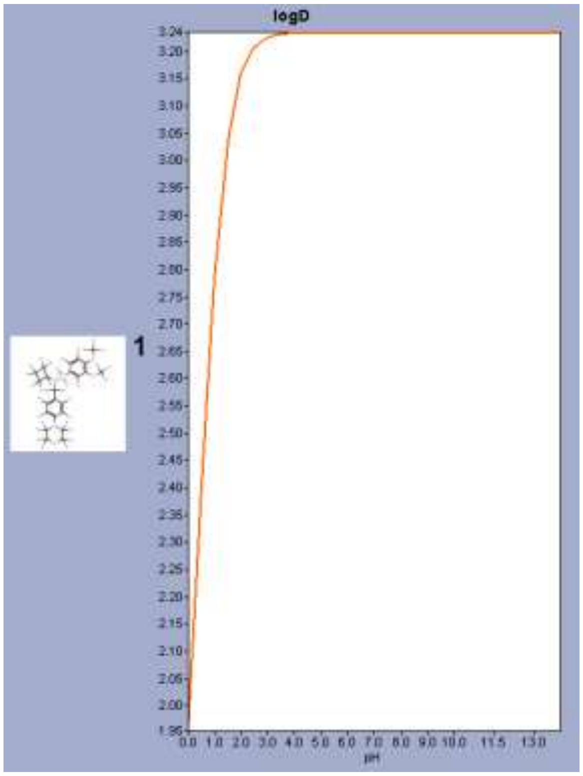

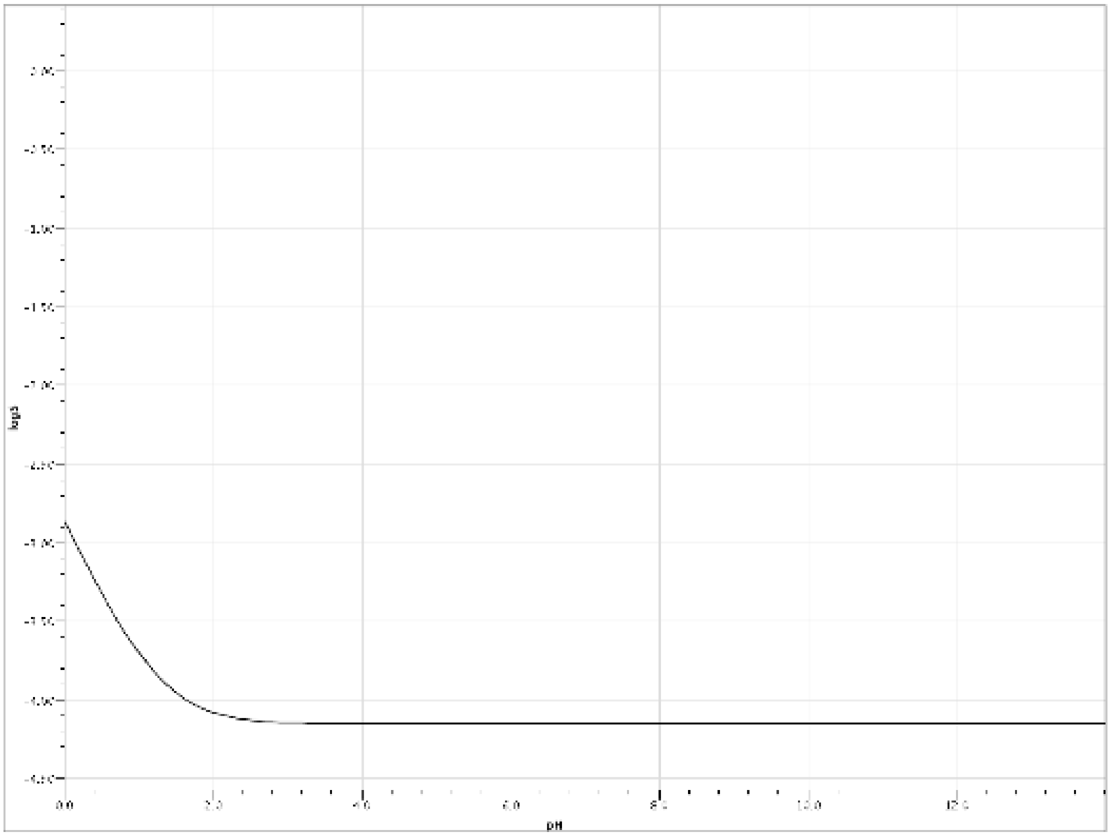

5a

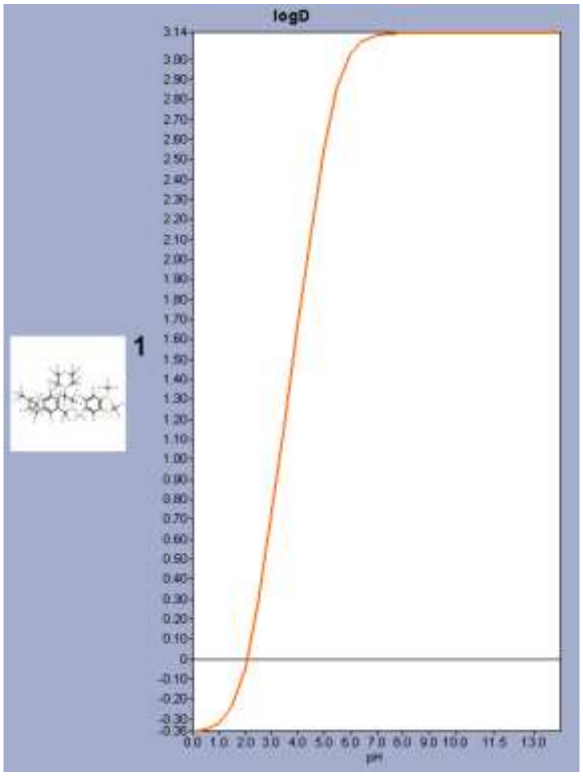

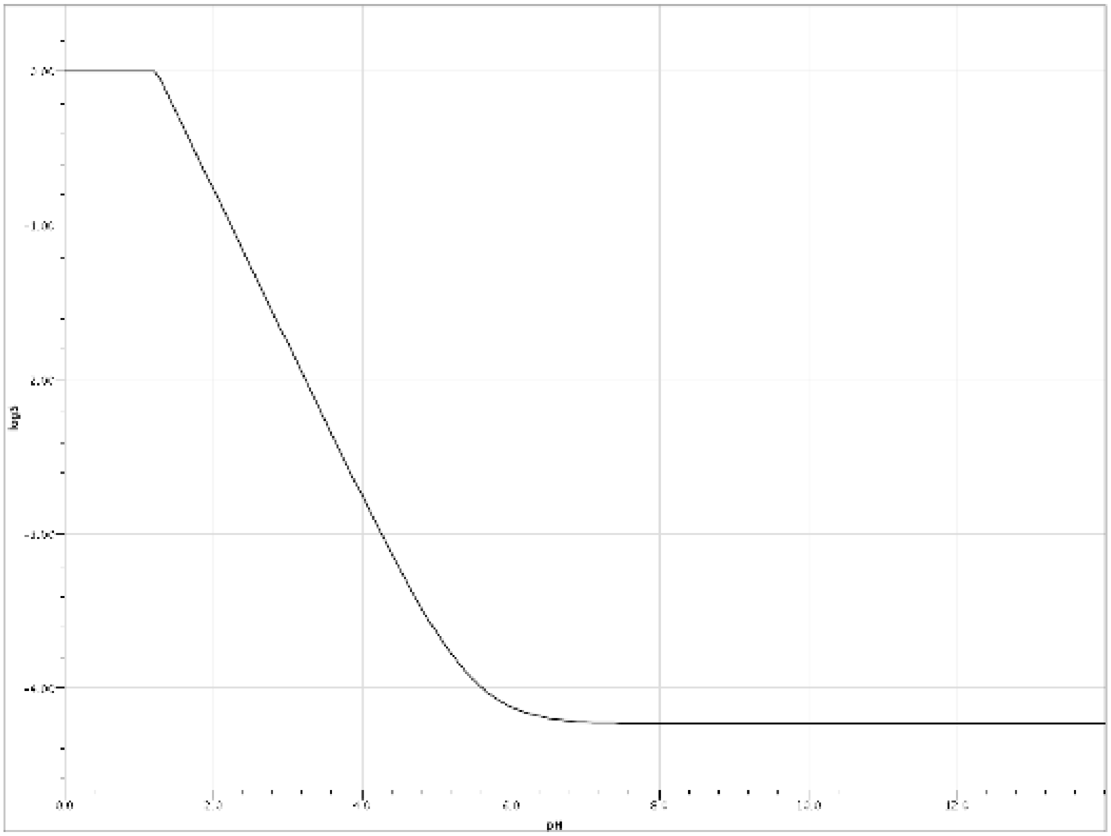

5b

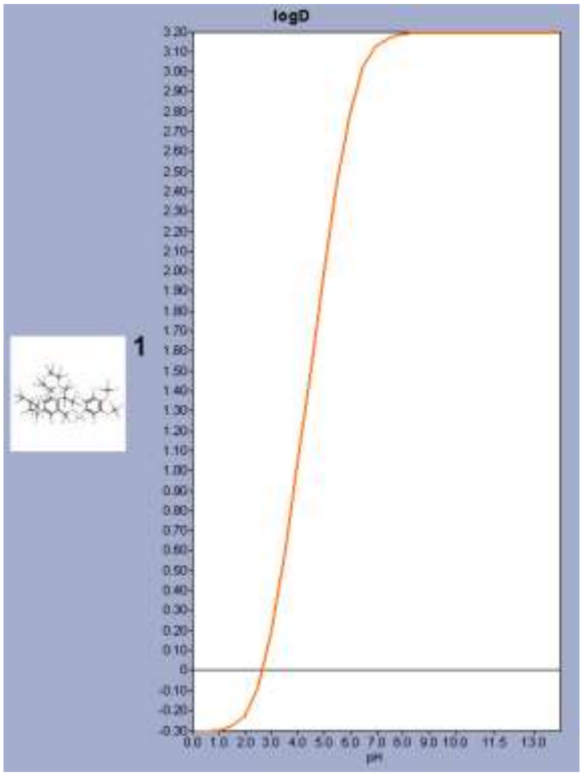

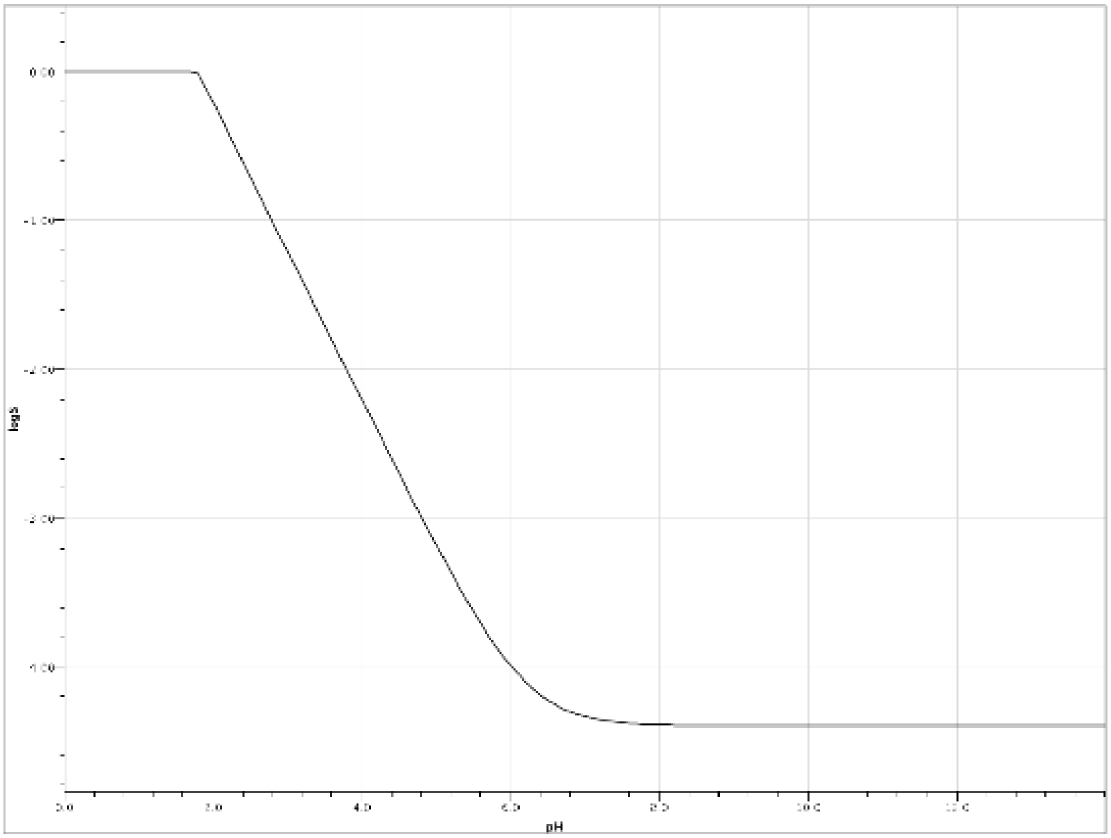

6a

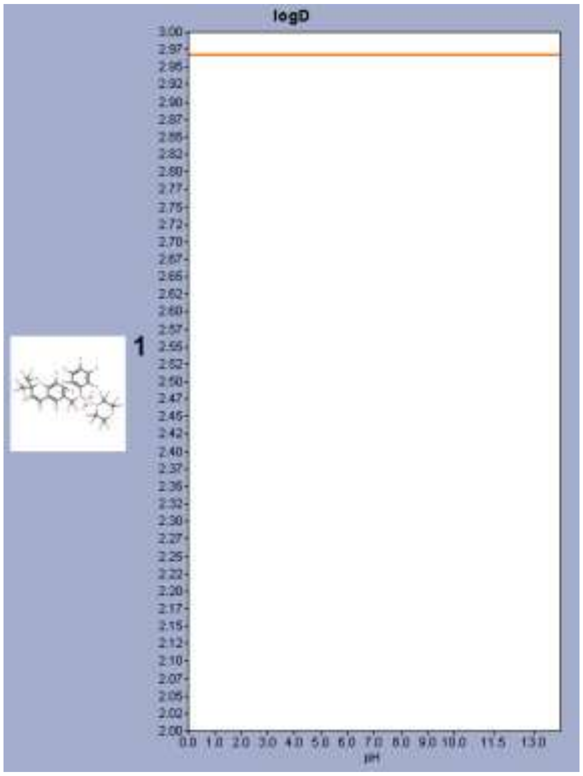

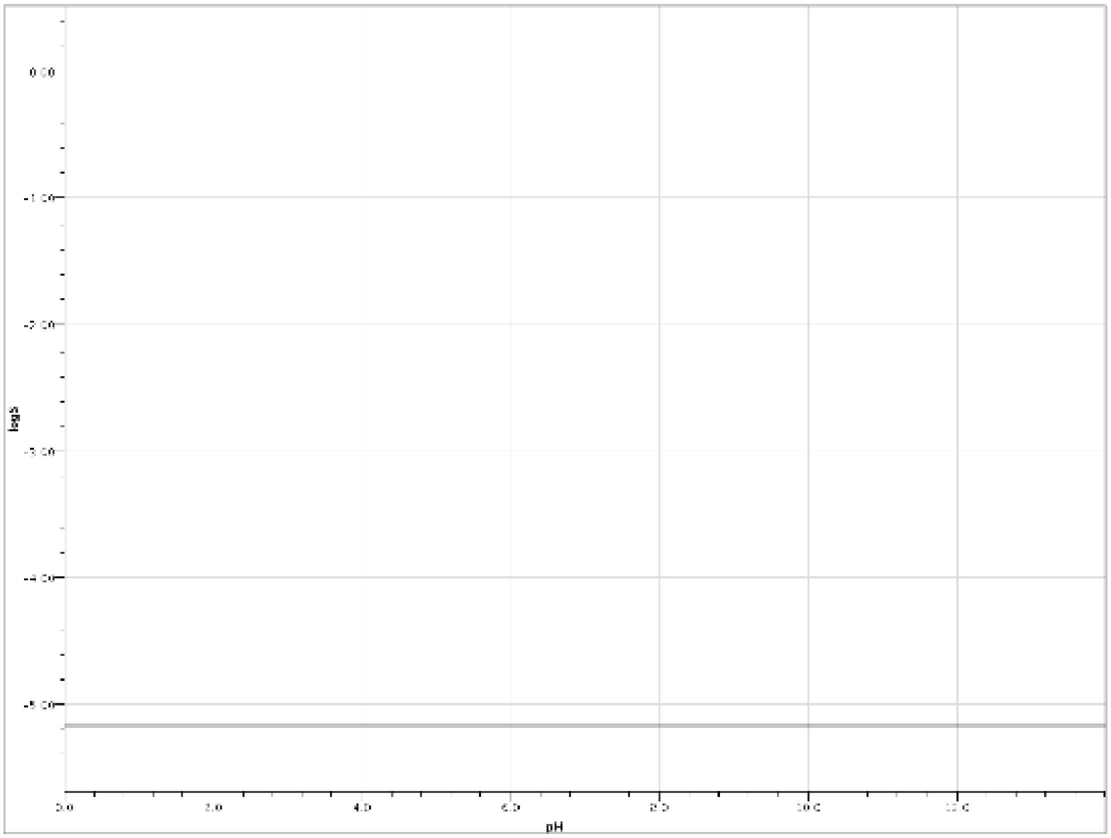

6b

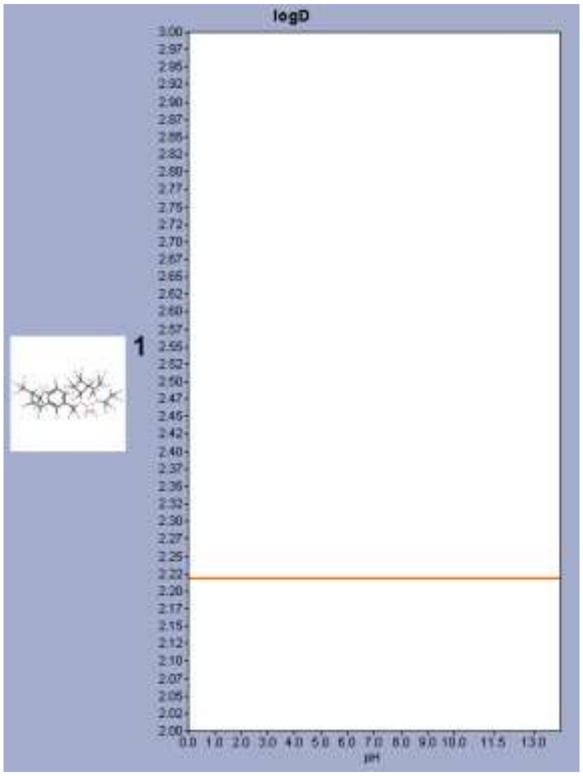

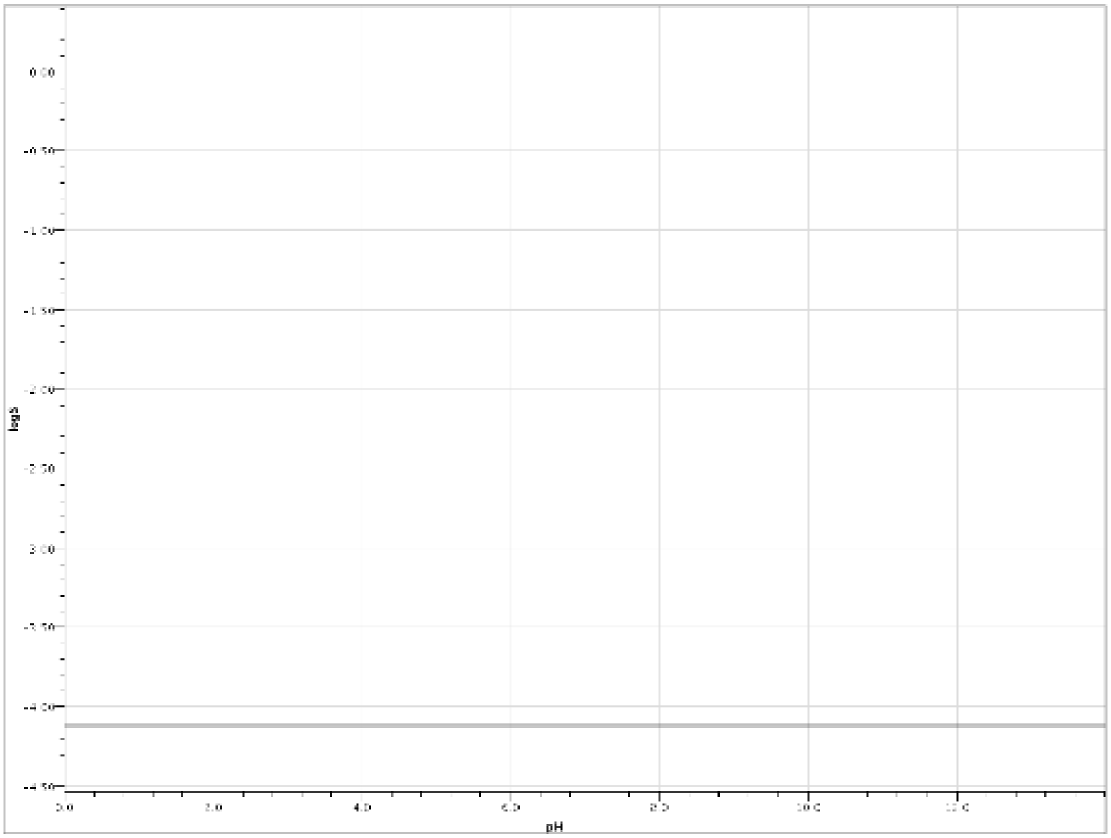

Supplement: IENZ_1347784_Supplementary_Material.pdf [file IENZ_A_1347784_SM3267.pdf]
